# Analysis of the impact of naturally-acquired immunity on *Shigella* vaccine performance to inform clinical trials: A simulation-based modeling study

**DOI:** 10.1371/journal.pmed.1004878

**Published:** 2026-10-21

**Authors:** Avnika B. Amin, Maria Garcia Quesada, Daniel Trudell, Natalie E. Dean, Benjamin A. Lopman, James A. Platts-Mills, Elizabeth T. Rogawski McQuade

**Affiliations:** 1 Department of Biostatistics and Bioinformatics, Rollins School of Public Health, Emory University, Atlanta, Georgia, United States of America; 2 Department of Epidemiology, Rollins School of Public Health, Emory University, Atlanta, Georgia, United States of America; 3 Division of Infectious Diseases & International Health, University of Virginia, Charlottesville, Virginia, United States of America; Institut Pasteur, FRANCE

## Abstract

**Background:**

Effective vaccines against *Shigella*, the leading bacterial cause of diarrheal disease globally, could prevent morbidity and mortality from shigellosis as well as limit antibiotic resistance. Several *Shigella* vaccine candidates are in late stages of development, and the design of large Phase 3 trials in target populations is underway. In high incidence settings, children are expected to rapidly accrue natural immunity such that after repeated pathogen exposure, unvaccinated infants may have similar levels of immunity as vaccinated infants. For this reason, in these settings estimates of vaccine efficacy (VE) may underestimate expected efficacy in a fully naïve population. To set expectations and support optimal planning of future *Shigella* vaccine trials, we aimed to quantify the potential impact of this phenomenon on VE estimates under different surveillance strategies and assumptions about *Shigella* immunity.

**Methods and findings:**

We simulated *Shigella* vaccine trials in low-, medium-, and high-burden settings under different assumptions about vaccine- and infection-conferred immunity. We estimated VE against severe, *Shigella*-attributable disease and any *Shigella*-attributable diarrhea under six trial designs: active surveillance for *Shigella* infection or symptom-based reporting paired with single-outcome, stratified recurrent outcome, or crude recurrent-outcome Cox proportional hazards models. In each scenario, we evaluated bias, 95% confidence interval (CI) coverage, and the false negative rate. Only the trial design using active surveillance for infection and single outcome models was consistently unbiased across all examined scenarios. The other five designs underestimated VE by up to an absolute 20%, with a single outcome model paired with symptom-based reporting performing least poorly. Bias generally was worse at higher-burden sites. For realistically sized trials, the 95% CI coverage and false negative rate were also worse at higher-burden sites. The results were sensitive to input parameters which depended on assumptions about *Shigella* natural history based on limited published data.

**Conclusions:**

The observed VE in *Shigella* vaccine trials is likely to underestimate true VE, though the magnitude of underestimation described here is conditional on the simulation’s inputs and assumptions. Careful trial design is needed to minimize this underestimation.

## Author summary

**Why was this study done?:**

- *Shigella* vaccines are soon to be evaluated in large field trials.
- However, evidence of effectiveness from such studies may be affected by natural exposure to *Shigella*.
- Quantification of this impact will set expectations and support optimal planning of future *Shigella* vaccine trials.

**What did the researchers do and find?:**

- We conducted a simulation study of *Shigella* vaccine trials in low-resource settings and evaluated the impact of alternative design and analysis strategies on vaccine efficacy (VE) estimates.
- We found that to recover unbiased estimates of VE in a fully susceptible population, ascertainment of all infections and accounting for them in analysis is necessary. This includes ascertainment of asymptomatic infections, which is rarely conducted in trials that surveil diarrhea symptoms only.
- Otherwise, VE may be underestimated, particularly so at higher-burden sites.

**What do these findings mean?:**

- The magnitude of underestimation of VE can be considerable, but the estimates depend on assumptions about *Shigella* biology and transmission, which are uncertain.
- Consideration of the effect of naturally acquired immunity on VE estimates should be a key part of trial planning.
- Heightened infection ascertainment and specific analysis strategies can be used to minimize impact.

## Abstract 3

Avnika Amin and colleagues use mathematical modelling to assess the effect of acquired immunity by natural Shigella infection on trial-derived estimates of vaccine efficacy to help inform trial design.

## Introduction

*Shigella* is the leading bacterial cause of diarrheal disease globally [1,2]. Children aged <5 years experience the most *Shigella*-related morbidity and mortality [3], and children in low-resource settings, particularly in South Asia and sub-Saharan Africa, are most affected [1,4]. Beyond the immediate morbidity and mortality from disease, *Shigella* can also have longer-term effects like linear growth faltering [5,6], which has been associated with increased susceptibility to infection, delays in development, and worsening of undernutrition [7,8]. While *Shigella* is treatable with antibiotics, diagnostic testing is largely unavailable in low-resource settings [9], and antimicrobial resistance is increasing [10]. Effective *Shigella* vaccines are poised to prevent poor outcomes from shigellosis and potentially reduce further antibiotic resistance. Several *Shigella* vaccine candidates are currently being evaluated, with Phase 3 studies in target populations in the planning phase [9,11].

Robust planning of Phase 3 *Shigella* trials requires both accurate expectations for vaccine efficacy (VE) and an understanding of how immunity mechanisms affect appropriate trial design and analysis. Lessons from rotavirus vaccines provide a cautionary tale. Rotavirus vaccines have lower VE in high-burden compared to low-burden settings [12,13]. Explanations like nutritional deficiencies, co-administered oral poliovirus vaccine, and passively-acquired antibody interference were initially proposed, but interventions to address these factors only modestly improved rotavirus vaccine immunogenicity [14]. At least some of the discrepancy in VE between high- and low-resource settings may be due to how quickly unvaccinated infants accrue infection-acquired immunity [15]. The faster they immunologically catch up to vaccinated infants due to higher force of infection in high-burden settings, the more that VE estimated in a trial underestimates VE in a fully susceptible population [16]. This phenomenon will also likely affect the evaluation of *Shigella* vaccines and pose a key challenge for planning clinical trials and interpreting trial results.

To support the optimal evaluation of *Shigella* vaccines, we conducted a simulation study to quantify the potential impact of accrued infection-acquired immunity on VE estimates from a hypothetical phase III efficacy trial. We evaluated the impact of varying the force of infection, the strength of infection-acquired immunity compared to vaccine-conferred immunity, post-vaccination immunity gains, and vaccine effects on infection risk. We then evaluated whether different trial designs and analytical strategies may improve the ability of the trial to estimate a VE that approximates the targeted VE in a fully susceptible population, i.e., the VE that is most relevant in a regulatory context. These findings can inform trial site selection, priority gaps in our understanding of immunity, and protocols for data collection and analysis.

## Methods

### Simulation structure

We simulated a Phase 3 vaccine trial where healthy, immune-naïve infants were enrolled and 1:1 randomized to two doses of either a placebo or a hypothetical *Shigella* vaccine. Enrollment of healthy, immune-naïve infants is expected for *Shigella* trials, as was done for rotavirus vaccine trials among infants [16]. Doses were administered at 6.5 and 9.5 months of age to be compatible with the Expanded Programme on Immunization (EPI) schedule. After a 2-week immune ramp-up period, follow-up to ascertain primary outcomes began at 10 months of age. To characterize the broader relationships between the parameters varied in each simulation scenario while minimizing variability due to small sample size, we first simulated an unrealistically large trial cohort. We assumed that enrolled infants remained infection-naïve between enrollment and the start of follow-up. We simulated recurrent *Shigella* infections between 10 and 36 months of age, when follow-up ended, and assumed no loss to follow-up. The recurrent infection process is conceptually represented and described in Fig A in S1 Appendix.

To simulate recurrent *Shigella* infections, we modeled (Equation A in S1 Appendix) (i) the hazard of infection over time, (ii) the conditional probability of progressing to diarrhea given infection, and (iii) the conditional probability of experiencing severe diarrhea given symptoms. We model each component as a function of age group, vaccination status, and infection-acquired immunity. *Shigella* natural histories that were identified in the Malnutrition and Enteric Diseases (MAL-ED) study, a longitudinal birth cohort study of the first 2 years of life, informed age trends in hazards and severity probabilities for the 10–12, 13–15, 16–18, 19–21, and 22–24 month age groups [17]. Based on studies reporting declines in *Shigella* incidence and severity after the second year of life [18], we assumed that hazards and severity probabilities peaked for the 22–24 month age groups and subsequently declined for the 25–30 and 31–36 month age groups. Modifications by vaccination status and infection-acquired immunity depended on the specific simulation scenario, described below.

### Simulation scenarios

In line with the target product characteristics [18], the vaccine was assumed to have 40% efficacy against Shigella diarrhea and 60% efficacy against severe Shigella diarrhea. For simplicity, we assumed that efficacy did not wane during follow-up, there was no loss to follow-up, and the population was homogeneous with respect to covariates.

We examined 50 different scenarios (summarized in Fig B in S1 Appendix) where four factors were varied: 1) the vaccine effect on infection hazard, 2) hybrid immunity (i.e., whether natural infection provided additional protection beyond vaccination), 3) the strength of infection-acquired immunity, and 4) the force of infection (i.e., hazard of infection among susceptible individuals in the population). VE against infection (factor 1) was either 0% (i.e., no effect on the infection hazard) or 20%. For hybrid immunity (factor 2), vaccinated infants either did not acquire additional immunity from natural infections or acquired additional immunity from, at most, one infection. The degree of protection after an infection, or infection-acquired immunity (factor 3) was equivalent whether the infection was asymptomatic or symptomatic and was either comparable, inferior, or superior to vaccination. If inferior, infection reduced the probability of subsequent diarrhea by 10% and the conditional probability of a severe outcome by 20%. If superior, infection reduced the infection hazard by 25%, the conditional probability of subsequent diarrhea by 35%, and the conditional probability of a severe outcome by 45%. Superior infection-conferred immunity was only evaluated when hybrid immunity was possible, since otherwise vaccinated infants would never achieve the maximum possible immunity. In scenarios with inferior or similar infection-conferred immunity, unvaccinated infants could accrue immunity until they reached the maximum possible immunity for a vaccinated infant. Conversely, in scenarios with superior infection-conferred immunity, vaccinated infants could accrue immunity until they reached the maximum possible immunity reachable by unvaccinated infants.

We evaluated five forces of infection (factor 4) to reflect *Shigella* burdens at different trial sites in low-resource settings: very low burden (based on incidence at the Enterics for Global Health [EFGH] Malawi site), low burden (based on incidence in the MAL-ED Brazil site), medium burden (based on incidence at the EFGH Peru site), high burden (midpoint between low and very high burden sites), and very high burden (based on incidence in the MAL-ED Bangladesh site) [17,19]. Infection incidence rates, as well as progression to expected diarrhea and severe diarrhea incidence rates, are in Table A in S1 Appendix.

### Trial structure for analysis

Before data analysis, we selected data from the simulations to be consistent with two types of data collection protocols: active infection surveillance and passive symptom-based reporting. For the (unrealistic) trial setting of active infection surveillance, we assumed all diarrhea episodes and subclinical infections would be identified in the trial, which allows for subclinical infections that elicit immunity to be incorporated into analysis. In the symptom-based reporting setting, we assumed all diarrhea episodes would be identified in the trial, but not subclinical infections. This is more consistent with common and feasible approaches to outcome ascertainment that are based on participants presenting to care when ill or by continuously monitoring participants for symptoms. In this setting, subclinical infections are not observed and cannot be considered in analysis.

### Data analysis

All analyses used a Cox proportional hazards model with time since 10 days after the second dose (e.g., at the start of expected full vaccine protection) as the underlying time scale and trial arm (placebo or vaccine) as the only covariate. We estimated VE against both any and severe *Shigella* diarrhea. VE was calculated as (1-hazard ratio) * 100% over the full 27 month follow-up period (10 days after second dose to 36 months of age) and stratified by the approximate first (1–15 months of follow-up; age 10–24 months) and second years (16–27 months of follow-up; age 25–36 months) of follow-up. 95% confidence intervals (CIs) for the VE estimates were calculated similarly with the CI bounds for the hazard ratio.

We evaluated three methods to estimate VE: a single outcome model, a stratified recurrent outcome model, and a crude recurrent outcome model. The single outcome model censored individuals after the first observed *Shigella* episode, which was either *Shigella* infection (regardless of symptoms) when active infection surveillance was used or *Shigella* diarrhea when symptom-based reporting was used. The recurrent outcome models did not censor individuals and included multiple endpoints for individuals if they occurred. The stratified model allowed the baseline hazard to differ for individuals with different observed *Shigella* episode histories. The crude model assumed a constant baseline hazard unaffected by event history. For recurrent outcome models, robust variances were used to account for correlated outcomes from the same individuals and the first 2 days after a *Shigella* episode were excluded from analysis time to reflect that a new episode could not be defined until after at least two diarrhea-free days.

We evaluated a total of six approaches based on the structure for data collection and analytic approach: active and passive surveillance paired with all three models (summarized in Table B in S1 Appendix). All six approaches were applied to the output data from each simulation run.

To compare VE results, we examined bias and mean squared error (MSE). Bias was calculated as the average VE estimate for a set of simulations minus the true VE in a fully susceptible population, while MSE was calculated as the average of the square of the estimate minus the true VE parameter [20]. We evaluated the percent of 95% CIs that contained the true VE parameter (coverage) and the percent that incorrectly indicated a nonsignificant effect (false negative rate).

### Trial simulations

To establish relationships between the four factors, we simulated 500 large *Shigella* vaccine trials with high statistical power for each scenario. In these large trials, 5,000 infants were enrolled in each trial arm (i.e., more than double the size of several rotavirus vaccine trials in low- and middle-income countries (LMICs) [21,22]. To then evaluate results that might be observed in realistically sized *Shigella* vaccine trials, we used data from the first 400 infant IDs in each trial arm to represent pilot data for sample size calculations (Table BB in S1 Appendix). We determined the minimum size *n* required in each trial arm for 80% power to detect 60% VE against severe *Shigella* diarrhea. We calculated *n* with the powerSurvEpi R package [23] based on the single outcome model. For each scenario, we used the median *n* for trial arm enrollment to simulate 2,500 trials, then repeated the six previously-described analytic approaches on each run’s output data. The same random seed was used across scenarios for the same numbered run, and calculations for the total number of runs needed are described in Equation B in S1 Appendix.

### Sensitivity analyses

For simplicity, we only evaluated one scenario: one where vaccination did not affect the infection hazard, infection-acquired immunity was comparable to vaccination, hybrid immunity does not occur, and the trial was conducted at a medium-burden site. First, to understand how bias would change with different true VEs in a fully susceptible population, we varied VE against any *Shigella* diarrhea between 10% and 90%. We set VE against severe *Shigella* diarrhea to the same value as VE against any *Shigella* diarrhea to avoid a scenario where vaccination would make severe *Shigella* diarrhea more likely, given symptomatic *Shigella* infection. Second, to examine what VE estimates during the first and second year of trial follow-up might look like in the presence of biologic waning, we reduced the vaccine effect on conditional severity probabilities by 25% during the last 12 months of trial follow-up. We then analyzed VE during the approximate first (follow-up months 1–15) and second (months 16–27) years of follow-up, which mirrored when waning first occurred. All simulations and analyses were conducted in R v4.0.6, with code available on GitHub.

Given the number of scenarios and performance metrics examined, we parse out key findings that are consistent when only one aspect of a scenario is changed. We focus the results on VE against severe *Shigella* diarrhea, as that is the expected primary endpoint for candidate *Shigella* vaccine trials. The full results for VE estimates against any and severe *Shigella* diarrhea across all simulation scenarios and analytic approaches are presented in Tables C-AA in S1 Appendix.

### Use of AI Disclosure

AI tools and technologies were not used in the conduct of this study. All authors are responsible for the final manuscript.

## Results

### Comparison of approaches in large trials

When trials had highly statistical power (i.e., 5,000 infants in each trial arm) and true VE against severe *Shigella* diarrhea in a fully susceptible population was 60%, only the trial design using active surveillance and a single outcome model yielded unbiased results (Fig 1, Table C in S1 Appendix). Under this trial design, the mean estimate of VE against severe *Shigella* diarrhea was 59.5% from trials conducted at a high-burden site when infection- and vaccine-conferred immunity were comparable, vaccination did not reduce the infection hazard, and hybrid immunity did not occur (Table C in S1 Appendix). However, mean VE estimates ranged from 47.3% to 51.7% (bias of an absolute −8.3% to −12.7%) when using the other five trial designs in the same scenario (Tables D-H in S1 Appendix). For most trial designs (excluding single outcome active surveillance), the underestimation of VE in a fully susceptible population worsened as the force of infection increased (Fig 1, Tables D-H in S1 Appendix). The following quantitative results continue to focus on the example scenario of comparable infection- and vaccine-acquired immunity, no vaccine-conferred protection against infection, and no hybrid immunity, though the trends are seen across scenarios.

**Fig 1:**
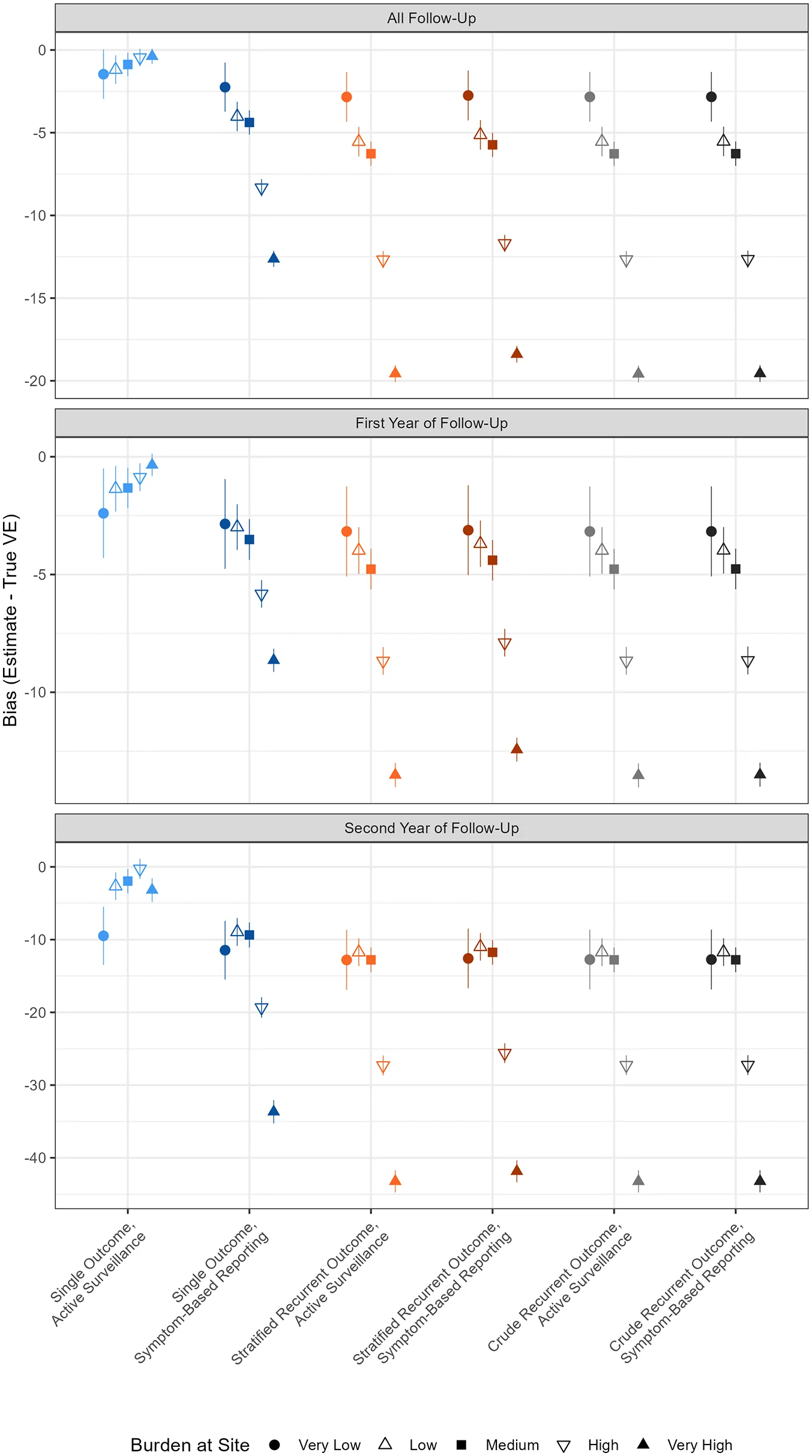
Bias of the estimated vaccine efficacy (VE) against severe, *Shigella*-attributable disease and 95% confidence intervals for well-powered trials with 5,000 infants in each trial arm when different approaches to data collection and analysis are used. **A:** VE is estimated for the full follow-up period (10–36 months); **B:** VE is estimated for the first year of follow-up (10–22 months); **C:** VE is estimated for the second year of follow-up (23–34 months). True VE in a fully susceptible population against severe *Shigella* diarrhea is 60%. A negative bias value corresponds with an underestimate of VE. These results derive from scenarios when infection- and vaccine-conferred immunity are comparable, vaccination does not reduce the infection hazard, and no hybrid immunity is possible.

At very low, low, and medium burden sites, bias did not substantially differ between the biased analysis approaches, with mean estimates of VE against severe *Shigella* diarrhea ranging from 57.2% to 57.8%, 54.5% to 56%, and 53.7% to 55.6% versus the input 60% VE (Tables D-H in S1 Appendix). However, as the force of infection increased, results from symptom-based reporting paired with a single outcome model were less biased than approaches using recurrent outcome models. Mean VE estimates from symptom-based reporting with a single outcome model were 51.7% and 47.4% at high and very high burden sites, respectively (corresponding to bias of −8.3% and −12.6%; Table D in S1 Appendix). In contrast, bias from the recurrent outcome model approaches neared −15% and −20% for the respective sites. The magnitudes of bias for the VE estimates from the first year of follow-up were slightly smaller than those from the full follow-up time (Fig 1, Tables C and I in S1 Appendix). However, bias was substantially larger when estimating VE during the second year of follow-up, in some cases exceeding −40% at the very high burden site.

As expected for trials with high statistical power, VE results were generally very precise. Coverage of 95% CIs was approximately 95% for most scenarios involving very low, low, or medium burden sites but much lower for those involving high or very high burden sites (Tables C-H in S1 Appendix). False negatives were rare for all but the scenarios involving very low burden sites, where outcomes were rarer and results were less precise.

### Comparison of approaches in realistic trial sizes

When more realistic sample sizes were used for trials (Tables J-O in S1 Appendix), bias was comparable to that seen with well-powered trials for each approach. 95% CI coverage was >90% at very low, low, and medium burden sites regardless of the trial design approach used. However, the 95% CI coverage and the false negative rate worsened as the force of infection increased for all approaches except when active surveillance was paired with a single outcome model (Fig 2, Tables I-N in S1 Appendix). A single outcome model with symptom-based reporting was the next best option, as it had a lower false negative rate and better 95% CI coverage than designs using recurrent outcome models. For example, at a very high burden site, pairing a single outcome model with symptom-based reporting meant that 78.8% of 95% CIs contained the true VE and a false conclusion of no vaccine effect was drawn in 21.4% of simulation runs (Fig 2, Table K in S1 Appendix), while 95% CIs from recurrent model approaches had 56.5% to 60.4% coverage and 29.4% to 31.5% would result in a false conclusion of no vaccine effect (Fig 2, Tables L-O in S1 Appendix).

**Fig 2:**
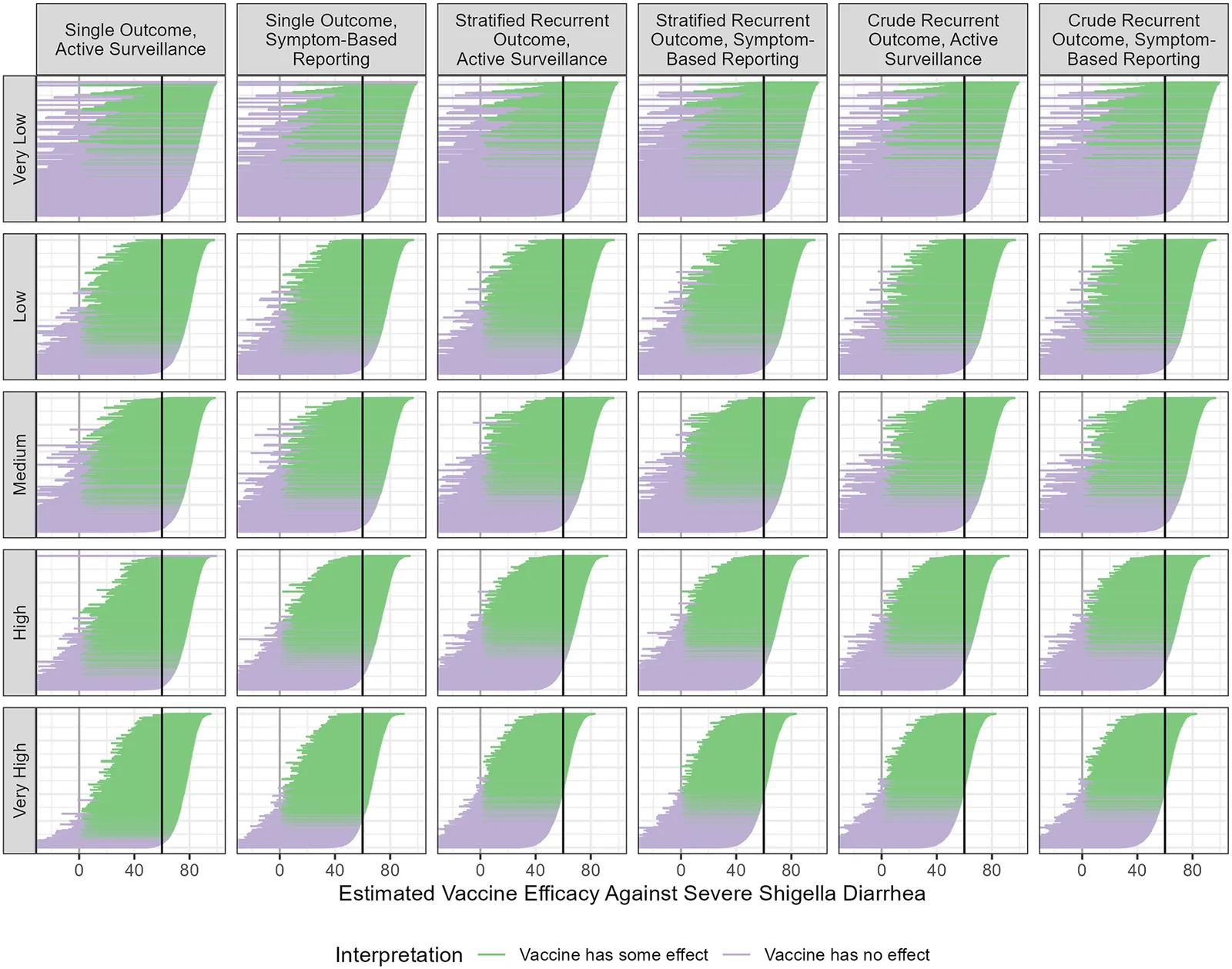
Estimated vaccine efficacy (VE) against severe *Shigella* diarrhea and 95% confidence intervals for trials with realistic sample sizes under different *Shigella* forces of infection and trial designs. The thick gray vertical rule indicates the null (0% efficacy), while the thick black vertical rule indicates the truth (60% efficacy). Purple indicates false negatives and green indicates detection of a statistically significant effect in the correct direction. These results reflect scenarios when infection- and vaccine-conferred immunity are comparable, vaccination does not reduce the infection hazard, and hybrid immunity does not occur.

### Alternative assumptions about immunity

The bias of VE estimates differed depending on the assumptions made about infection- and vaccine-conferred immunity. Pairing active surveillance with a single outcome model continued to yield unbiased results regardless of the immunity scenario (Tables C and I in S1 Appendix). For the remainder of this section, we will focus on describing results for the five designs with the most bias (symptom-based reporting with a single outcome model and active surveillance and symptom-based reporting paired with stratified and crude recurrent outcome models) in the large trial (5,000 infants per arm).

Assumptions around hybrid immunity appeared most influential. Estimates from scenarios where hybrid immunity was possible were only slightly biased regardless of the other scenario conditions or trial design used (Tables D-H in S1 Appendix). Scenarios where hybrid immunity was not possible resulted in more extreme underestimation of VE in a fully susceptible population. For example, at a high burden site when infection-conferred immunity was similar to or worse than vaccine-conferred immunity, mean bias ranged from −4.1% to −12.9% when hybrid immunity was not possible but only from −0.3% to −3.4% when hybrid immunity was possible, regardless of the vaccine’s effect on infection hazard (Fig 3, Tables D-H in S1 Appendix).

**Fig 3:**
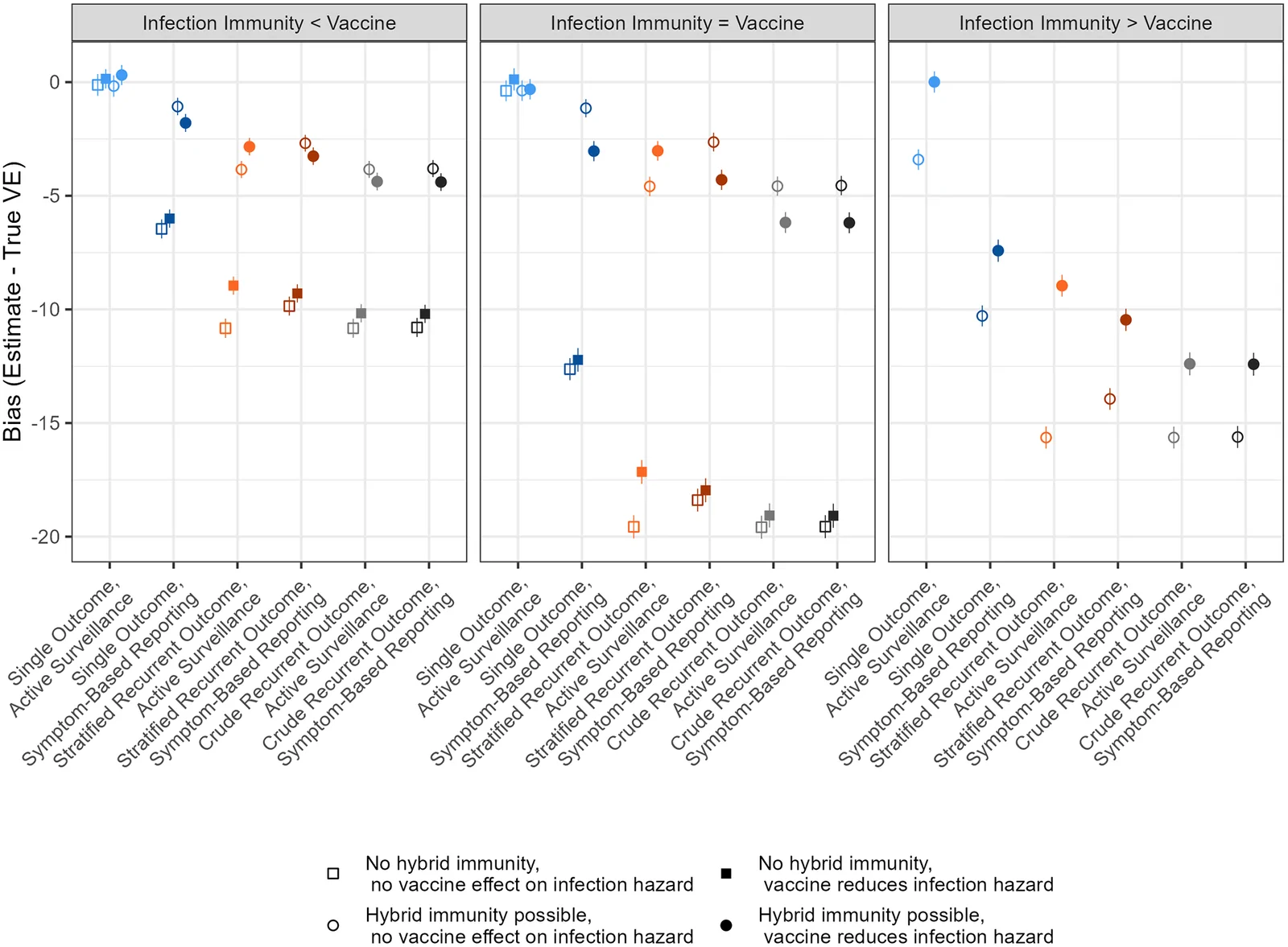
Bias of the estimated vaccine efficacy (VE) against severe, *Shigella*-attributable disease and 95% confidence intervals for well-powered trials under different scenarios of vaccine- and infection-conferred immunity. True VE is 60%. A negative bias value corresponds with an underestimate of VE. These results reflect trials conducted at a high burden site. For scenarios where immunity conferred per infection was greater than vaccine-conferred protection, a lack of hybrid immunity was not evaluated.

Bias worsened as the immunity conferred per infection increased relative to the vaccine’s effect, with this most noticeable when infection-conferred immunity was greater than vaccine-conferred immunity (Fig 3, Tables D-H in S1 Appendix). This factor mattered more when hybrid immunity was not possible, with worse bias when infection- and vaccine-conferred immunity were comparable. When hybrid immunity was not possible and a high burden site was used, mean bias ranged from −4.1% to −6.7% if infection-conferred immunity was less protective than vaccination (Fig 3, Tables D-H in S1 Appendix). However, when infection- and vaccine-conferred immunity were similar, mean bias ranged from −9.3% to −12.9% (Fig 3, Tables D-H in S1 Appendix). No clear trends emerged when comparing scenarios where vaccination did and did not affect the hazard of infection.

### Sensitivity analyses

The true VE in a fully susceptible population also affected the magnitude of bias (Fig 4). Pairing active surveillance with a single outcome model resulted in no bias for any VE against any *Shigella* diarrhea. Bias for the other designs showed a U-shaped relationship, with small bias for small or large true VE, and larger bias as VE approached 50%. Consistent with prior results, designs that used recurrent outcome models were more biased than the design pairing symptom-based reporting with a single outcome model.

**Fig 4:**
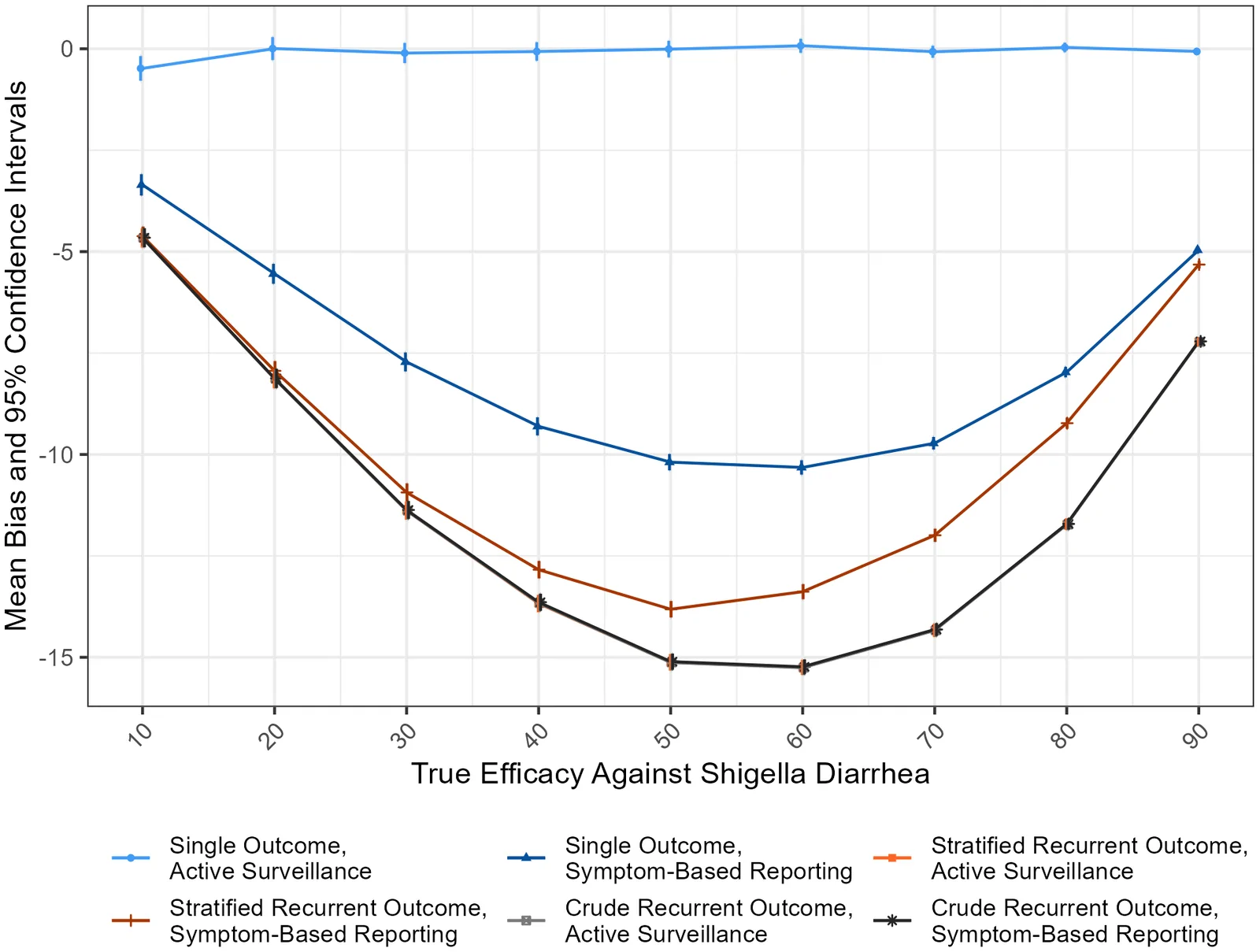
Mean bias of the estimated vaccine efficacy (VE) against any *Shigella* diarrhea with varying true efficacy in a fully susceptible population and 95% confidence intervals for well-powered trials conducted at a high burden site. A negative bias value corresponds with an underestimate of VE. These results reflect scenarios when infection- and vaccine-conferred immunity are comparable, vaccination does not reduce the infection hazard, and no hybrid immunity is possible. Note: estimates from the stratified recurrent outcome, active surveillance, and the two crude recurrent outcome models are nearly overlapping.

The presence of biologic waning of vaccine protection did not meaningfully affect the magnitude of bias when evaluating VE during follow-up months 1–15 and 16–27 (Fig 5). Bias was minimal using the single outcome model paired with active surveillance during both follow-up periods, between −5.6% and −8.4% when using other approaches during the 1–15 month follow-up, and between −15% and −22% when using other approaches during the 16–27 month follow-up period. This is comparable to the bias seen in the absence of biologic waning, which was minimal using the single outcome model paired with active surveillance during both the first and second years of follow-up, between −5% and −7.3% when using other approaches during the first year of follow-up, and between −16.3% and −23.3% when using other approaches during the second year of follow-up (Fig 1, Table I in S1 Appendix).

**Fig 5:**
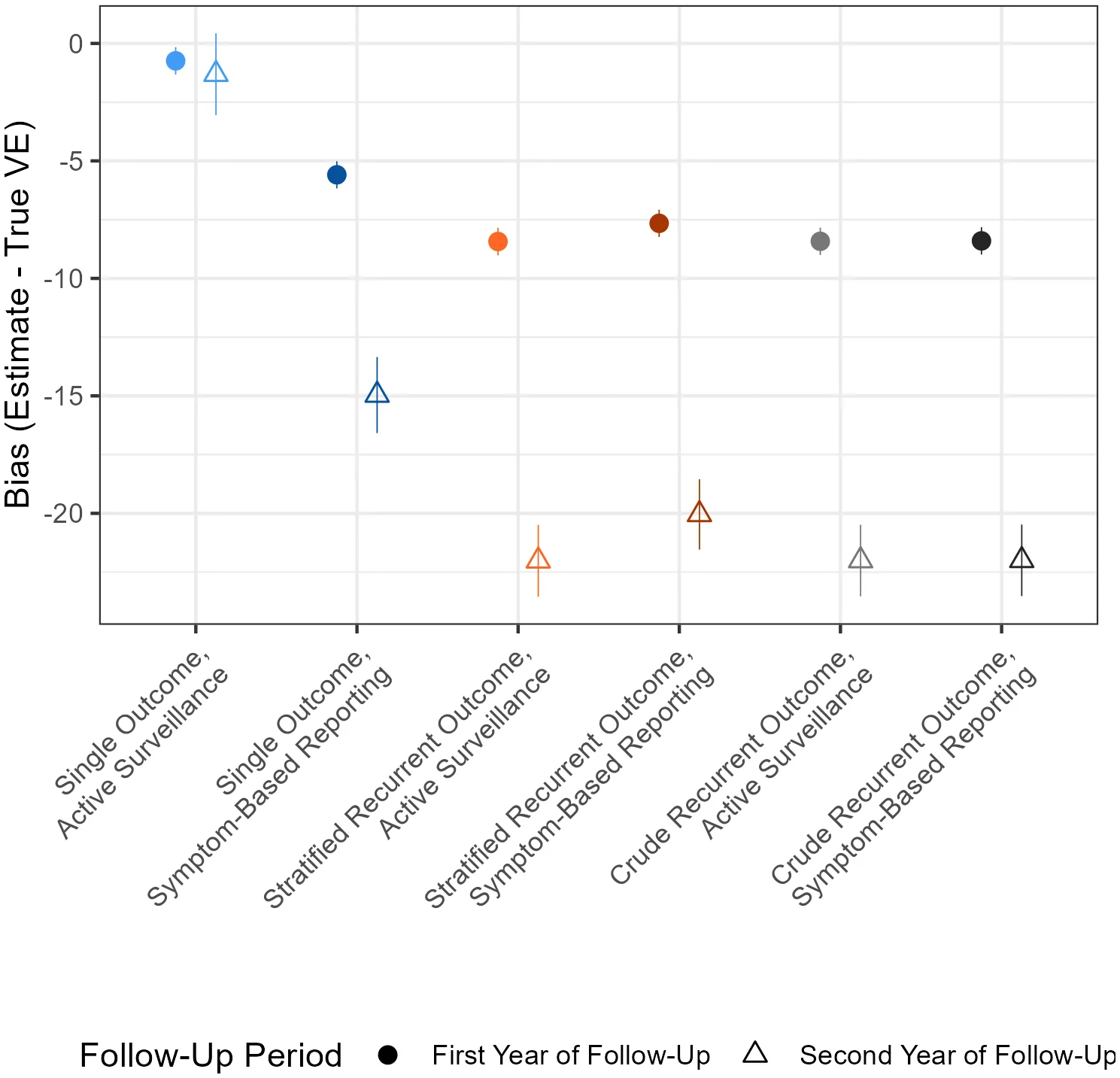
Mean bias of the estimated vaccine efficacy (VE) against severe, *Shigella*-attributable disease and 95% confidence intervals for well-powered trials with 5,000 infants in each trial arm when there is waning vaccine protection and different approaches to data collection and analysis are used. True VE against severe *Shigella* diarrhea is 60%. A negative bias value corresponds with an underestimate of VE. These results derive from the scenario when infection- and vaccine-conferred immunity are comparable, vaccination does not reduce the infection hazard, no hybrid immunity is possible, the trial takes place at a high burden site, and vaccine protection wanes.

## Discussion

We demonstrate that the VE observed in future *Shigella* vaccine trials is likely to underestimate true VE in a fully susceptible population because of the accrual of natural immunity. While the observed VE will faithfully capture VE specific to the setting of the trial, which is a distinct pragmatic setting-specific effectiveness estimand for which natural immunity will be a relevant factor, we argue that the target VE of interest in a regulatory context (i.e., in Phase III trials) is VE in a fully susceptible population, which is likely to be more generalizable across settings and better captures the “biological” vaccine effect. The impact of natural immunity on VE estimates is most extreme when this true VE is close to 50%.

The magnitude of underestimation of the target VE depends on several factors, with the force of infection being the most influential. We estimate that VE underestimation worsens as the force of infection increases, with the worst bias—VE estimates an absolute 20% too low—at high-burden sites. This is especially important as high-burden sites are often favored for clinical trials, since the larger number of expected events means that fewer infants need to be enrolled to obtain the desired statistical power. Our results suggest that trials at high-burden sites may lead to the incorrect conclusion about a null *Shigella* VE as often as 50%–60% of the time, and the lower sample size required due to high incidence will be offset by needing a larger sample size to detect a smaller VE. Importantly, because the impact of natural immunity increases with longer duration of follow-up, and is most striking for estimates specific to later periods of follow-up, studies in high-burden settings may incorrectly conclude that vaccine-induced immunity wanes rapidly, when the truth is rather that natural immunity was accruing over follow-up. While we characterize the impact of this phenomenon for *Shigella* specifically, the concept is likely equally relevant for the evaluation of vaccines against other pathogens in populations for which the force of infection is high and asymptomatic infections are common. We previously evaluated the impact on rotavirus VE [15] and suggest similar effects might affect malaria VE in high burden settings. Most enteric infections in low-resource settings are frequently detected in the absence of diarrhea [6], such that immunologic catch up is likely to affect vaccines in development for other enteric pathogens as well.

To some extent, decisions around a trial’s data collection protocol and analysis can mitigate the magnitude of underestimation at high-burden sites. Active surveillance for *Shigella* infection paired with a single outcome regression model, where individuals are censored at their first infection, was consistently unbiased no matter the site burden or other assumptions around immunity. However, active infection surveillance is unlikely to be realistic as it would require regular testing of asymptomatic individuals. Even symptom-based reporting (with either active or passive surveillance) is unlikely to detect all symptomatic cases, especially when detection of cases at presentation to care is the norm. Our results indicate that bias is most mitigated when a single outcome model is used, although VE will be underestimated by an average of an absolute 10% in most scenarios. In considering trial design, the value of a higher and more accurate estimate of VE should be weighed against the potential high cost of more complete outcome ascertainment. Intensified surveillance, such as using app-based self-reporting methods, or routine serology collection in a subset of participants for the full trial duration may provide a reasonable compromise. Additional surveillance at the community level for infection burden could also provide context for the trial results. In some settings, vaccine introduction may be justified by high disease burden even if the efficacy is underestimated.

Other aspects of infection- and vaccine-conferred immunity also affect expected bias, with the general principle being that bias is worse when unvaccinated infants can immunologically “catch-up” to vaccinated infants. For example, when hybrid immunity does not occur, vaccinated infants cannot gain additional immunity, which allows unvaccinated infants to more quickly catch up to them immunologically. A similar principle applies to our findings around the similarity between infection- and vaccine-conferred immunity. When the two are equal, unvaccinated infants only need one infection to catch up to a vaccinated infant; however, when infection-conferred immunity is inferior, unvaccinated infants must experience multiple infections to catch up to the vaccinated infants.

This study is subject to a few limitations. The primary limitation is that our results, particularly the magnitude of bias, will be sensitive to our input parameters. The numeric magnitudes of bias described may not exactly translate to the degree of VE underestimation from eventual Phase 3 trials. Very little is known about *Shigella* natural histories beyond data from the few longitudinal birth cohort studies [2,19,24], two of which informed our study. Relatedly, because of this lack of information, we did not explicitly model different *Shigella* serotypes and how VE for a vaccine that only covers some serotypes might be mismeasured, particularly if nonvaccine serotypes provide some protection against vaccine serotypes. The effect is likely similar to that of unobserved infections that confer immunity. We assumed that vaccine immunity did not wane during the follow-up period in the main simulation set, but waning is certainly possible. Depending on how quickly *Shigella* vaccine immunity wanes, bias may worsen since waning would allow unvaccinated infants to catch up to vaccinated infants more quickly. In our waning sensitivity analysis, this did not seem to be the case; however, it may be a more impactful factor when the force of infection is lower. We also assumed that all infants were homogeneous with respect to susceptibility and pre-existing immunity. Realistically, it may be difficult to ascertain an infant’s infection history, and trials may have to contend with unobserved differences in baseline infection-conferred immunity. The assumption of proportional hazards for the Cox models used in our analyses may not hold in every scenario and trial design approach, though this is unlikely to be the driving factor for observed trends. Finally, while death is an important secondary endpoint, we did not consider effects on VE estimates for that outcome since the risks of death are small enough to only have a minor impact on any Phase III trial.

Overall, we demonstrate how observed VE from clinical trials will underestimate VE in a fully susceptible population depending on the force of infection and mechanisms of immunity. These results help to set expectations for future *Shigella* vaccine trials and inform the selection of trial sites. Furthermore, our findings shed light both on key considerations for trial and analysis design and on gaps in knowledge that may affect *Shigella* vaccine trial results.

## Supporting information

S1 Appendix Supplementary Tables A–BB, Figures A and B, and Equations A and B. **Fig A.** Simulation structure and process for generating recurrent infections. **Equation A.** Multiplier approach to generate an individual’s infection hazard and disease progression probabilities based on their age, vaccination status, and infection history. **Fig B.** Full set of simulation scenarios with different assumptions around vaccine effects, infection-acquired immunity, hybrid immunity, and the force of infection. **Table A.** *Shigella* natural history parameters and infection incidence rates at low-, medium-, and high-burden sites. **Table B.** Summary of analytic approaches to evaluating ideal and realistic data generated from simulated clinical *Shigella* vaccine trials. **Equation B.** Calculating the number of simulations needed for target Monte Carlo standard errors. **Table C.** Mean estimate, bias, mean squared error (MSE), coverage, and false negative rate for vaccine efficacy (VE) estimates against severe *Shigella* diarrhea from highly powered trials for all 50 simulation scenarios when using active surveillance for infection and a single outcome regression model. **Table D.** Mean estimate, bias, mean squared error (MSE), coverage, and false negative rate for vaccine efficacy (VE) estimates against severe *Shigella* diarrhea from highly powered trials for all 50 simulation scenarios when using symptom-based reporting and a single outcome regression model. **Table E.** Mean estimate, bias, mean squared error (MSE), coverage, and false negative rate for vaccine efficacy (VE) estimates against severe *Shigella* diarrhea from highly powered trials for all 50 simulation scenarios when using active surveillance for infection and a stratified recurrent outcome regression model. **Table F.** Mean estimate, bias, mean squared error (MSE), coverage, and false negative rate for vaccine efficacy (VE) estimates against severe *Shigella* diarrhea from highly powered trials for all 50 simulation scenarios when using symptom-based reporting and a stratified recurrent outcome regression model. **Table G.** Mean estimate, bias, mean squared error (MSE), coverage, and false negative rate for vaccine efficacy (VE) estimates against severe *Shigella* diarrhea from highly powered trials for all 50 simulation scenarios when using active surveillance for infection and a crude recurrent outcome regression model. **Table H.** Mean estimate, bias, mean squared error (MSE), coverage, and false negative rate for vaccine efficacy (VE) estimates against severe *Shigella* diarrhea from highly powered trials for all 50 simulation scenarios when using symptom-based reporting and a crude recurrent outcome regression model. **Table I.** Mean estimate, bias, mean squared error (MSE), coverage, and false negative rate for vaccine efficacy (VE) estimates against severe *Shigella* diarrhea from highly powered trials for all 6 design approaches applied to stratified datasets for the first and second year of follow-up. **Table J.** Mean estimate, bias, mean squared error (MSE), coverage, and false negative rate for vaccine efficacy (VE) estimates against severe *Shigella* diarrhea from realistically sized trials for all 50 simulation scenarios when using active surveillance for infection and a single outcome regression model. **Table K.** Mean estimate, bias, mean squared error (MSE), coverage, and false negative rate for vaccine efficacy (VE) estimates against severe *Shigella* diarrhea from realistically sized trials for all 50 simulation scenarios when using symptom-based reporting and a single outcome regression model. **Table L.** Mean estimate, bias, mean squared error (MSE), coverage, and false negative rate for vaccine efficacy (VE) estimates against severe *Shigella* diarrhea from realistically sized trials for all 50 simulation scenarios when using active surveillance for infection and a stratified recurrent outcome regression model. **Table M.** Mean estimate, bias, mean squared error (MSE), coverage, and false negative rate for vaccine efficacy (VE) estimates against severe *Shigella* diarrhea from realistically sized trials for all 50 simulation scenarios when using symptom-based reporting and a stratified recurrent outcome regression model. **Table N.** Mean estimate, bias, mean squared error (MSE), coverage, and false negative rate for vaccine efficacy (VE) estimates against severe *Shigella* diarrhea from realistically sized trials for all 50 simulation scenarios when using active surveillance for infection and a crude recurrent outcome regression model. **Table O.** Mean estimate, bias, mean squared error (MSE), coverage, and false negative rate for vaccine efficacy (VE) estimates against severe *Shigella* diarrhea from realistically sized trials for all 50 simulation scenarios when using symptom-based reporting and a crude recurrent outcome regression model. **Table P.** Mean estimate, bias, mean squared error (MSE), coverage, and false negative rate for vaccine efficacy (VE) estimates against any *Shigella* diarrhea from highly powered trials for all 50 simulation scenarios when using active surveillance for infection and a single outcome regression model. **Table Q.** Mean estimate, bias, mean squared error (MSE), coverage, and false negative rate for vaccine efficacy (VE) estimates against any *Shigella* diarrhea from highly powered trials for all 50 simulation scenarios when using symptom-based reporting and a single outcome regression model. **Table R.** Mean estimate, bias, mean squared error (MSE), coverage, and false negative rate for vaccine efficacy (VE) estimates against any *Shigella* diarrhea from highly powered trials for all 50 simulation scenarios when using active surveillance for infection and a stratified recurrent outcome regression model. **Table S.** Mean estimate, bias, mean squared error (MSE), coverage, and false negative rate for vaccine efficacy (VE) estimates against any *Shigella* diarrhea from highly powered trials for all 50 simulation scenarios when using symptom-based reporting and a stratified recurrent outcome regression model. **Table T.** Mean estimate, bias, mean squared error (MSE), coverage, and false negative rate for vaccine efficacy (VE) estimates against any *Shigella* diarrhea from highly powered trials for all 50 simulation scenarios when using active surveillance for infection and a crude recurrent outcome regression model. **Table U.** Mean estimate, bias, mean squared error (MSE), coverage, and false negative rate for vaccine efficacy (VE) estimates against any *Shigella* diarrhea from highly powered trials for all 50 simulation scenarios when using symptom-based reporting and a crude recurrent outcome regression model. **Table V.** Mean estimate, bias, mean squared error (MSE), coverage, and false negative rate for vaccine efficacy (VE) estimates against any *Shigella* diarrhea from realistically sized trials for all 50 simulation scenarios when using active surveillance for infection and a single outcome regression model. **Table W.** Mean estimate, bias, mean squared error (MSE), coverage, and false negative rate for vaccine efficacy (VE) estimates against any *Shigella* diarrhea from realistically sized trials for all 50 simulation scenarios when using symptom-based reporting and a single outcome regression model. **Table X.** Mean estimate, bias, mean squared error (MSE), coverage, and false negative rate for vaccine efficacy (VE) estimates against any *Shigella* diarrhea from realistically sized trials for all 50 simulation scenarios when using active surveillance for infection and a stratified recurrent outcome regression model. **Table Y.** Mean estimate, bias, mean squared error (MSE), coverage, and false negative rate for vaccine efficacy (VE) estimates against any *Shigella* diarrhea from realistically sized trials for all 50 simulation scenarios when using symptom-based reporting and a stratified recurrent outcome regression model. **Table Z.** Mean estimate, bias, mean squared error (MSE), coverage, and false negative rate for vaccine efficacy (VE) estimates against any *Shigella* diarrhea from realistically sized trials for all 50 simulation scenarios when using active surveillance for infection and a crude recurrent outcome regression model. **Table AA.** Mean estimate, bias, mean squared error (MSE), coverage, and false negative rate for vaccine efficacy (VE) estimates against any *Shigella* diarrhea from realistically sized trials for all 50 simulation scenarios when using symptom-based reporting and a crude recurrent outcome regression model. **Table BB.** Number of participants in realistically sized trials for all 50 simulation scenarios based on sample size calculations.
(PDF)

## Abbreviations

CIs: confidence intervals
EFGH: Enterics for Global Health
EPI: Expanded Programme on Immunization
LMICs: low and middle-income countries
MSE: mean squared error
VE: vaccine efficacy.
