# Supplementary material for: Analysis of the impact of naturally-acquired immunity on Shigella vaccine performance to inform clinical trials: A simulation-based modeling study
Source: PLoS Med. 2026 Oct 21;23(10):e1004878. doi: 10.1371/journal.pmed.1004878 (PMC13719040; doi:10.1371/journal.pmed.1004878)
Supplement: Supplementary file 1 [file pmed.1004878.s001.pdf]

Planning robust clinical trials for *Shigella* vaccines: A simulation-based evaluation of the impact of naturally-acquired immunity on vaccine performance

Avnika B. Amin, Maria Garcia Quesada, Daniel Trudell, Natalie E. Dean, Benjamin A. Lopman, James A. Platts-Mills, Elizabeth T. Rogawski McQuade

S1 Appendix

Figure A. Simulation structure and process for generating recurrent infections.

Equation A. Multiplier approach to generate an individual's infection hazard and disease progression probabilities based on their age, vaccination status, and infection history.

Figure B. Full set of simulation scenarios with different assumptions around vaccine effects, infection-acquired immunity, hybrid immunity, and the force of infection.

Table A. *Shigella* natural history parameters and infection incidence rates at low-, medium-, and high-burden sites.

Table B. Summary of analytic approaches to evaluating ideal and realistic data generated from simulated clinical *Shigella* vaccine trials.

Equation B. Calculating the number of simulations needed for target Monte Carlo standard errors.

Table C. Mean estimate, bias, mean squared error (MSE), coverage, and false negative rate for vaccine efficacy (VE) estimates against severe *Shigella* diarrhea from highly powered trials for all 50 simulation scenarios when using active surveillance for infection and a single outcome regression model.

Table D. Mean estimate, bias, mean squared error (MSE), coverage, and false negative rate for vaccine efficacy (VE) estimates against severe *Shigella* diarrhea from highly powered trials for all 50 simulation scenarios when using symptom-based reporting and a single outcome regression model.

Table E. Mean estimate, bias, mean squared error (MSE), coverage, and false negative rate for vaccine efficacy (VE) estimates against severe *Shigella* diarrhea from highly powered trials for all 50 simulation scenarios when using active surveillance for infection and a stratified recurrent outcome regression model.

Table F. Mean estimate, bias, mean squared error (MSE), coverage, and false negative rate for vaccine efficacy (VE) estimates against severe *Shigella* diarrhea from highly powered trials for all 50 simulation scenarios when using symptom-based reporting and a stratified recurrent outcome regression model.

Table G. Mean estimate, bias, mean squared error (MSE), coverage, and false negative rate for vaccine efficacy (VE) estimates against severe *Shigella* diarrhea from highly powered trials for all 50 simulation scenarios when using active surveillance for infection and a crude recurrent outcome regression model.

Table H. Mean estimate, bias, mean squared error (MSE), coverage, and false negative rate for vaccine efficacy (VE) estimates against severe *Shigella* diarrhea from highly powered trials for all 50 simulation scenarios when using symptom-based reporting and a crude recurrent outcome regression model.

Table I. Mean estimate, bias, mean squared error (MSE), coverage, and false negative rate for vaccine efficacy (VE) estimates against severe *Shigella* diarrhea from highly powered trials for all 6 design approaches applied to stratified datasets for the first and second year of follow-up.

Table J. Mean estimate, bias, mean squared error (MSE), coverage, and false negative rate for vaccine efficacy (VE) estimates against severe *Shigella* diarrhea from realistically sized trials for all 50 simulation scenarios when using active surveillance for infection and a single outcome regression model.

Table K. Mean estimate, bias, mean squared error (MSE), coverage, and false negative rate for vaccine efficacy (VE) estimates against severe *Shigella* diarrhea from realistically sized trials for all 50 simulation scenarios when using symptom-based reporting and a single outcome regression model.

Table L. Mean estimate, bias, mean squared error (MSE), coverage, and false negative rate for vaccine efficacy (VE) estimates against severe *Shigella* diarrhea from realistically sized trials for all 50 simulation scenarios when using active surveillance for infection and a stratified recurrent outcome regression model.

Table M. Mean estimate, bias, mean squared error (MSE), coverage, and false negative rate for vaccine efficacy (VE) estimates against severe *Shigella* diarrhea from realistically sized trials for all 50 simulation scenarios when using symptom-based reporting and a stratified recurrent outcome regression model.

Table N. Mean estimate, bias, mean squared error (MSE), coverage, and false negative rate for vaccine efficacy (VE) estimates against severe *Shigella* diarrhea from realistically sized trials for all 50 simulation scenarios when using active surveillance for infection and a crude recurrent outcome regression model.

Table O. Mean estimate, bias, mean squared error (MSE), coverage, and false negative rate for vaccine efficacy (VE) estimates against severe *Shigella* diarrhea from realistically sized trials for all 50 simulation scenarios when using symptom-based reporting and a crude recurrent outcome regression model.

Table P. Mean estimate, bias, mean squared error (MSE), coverage, and false negative rate for vaccine efficacy (VE) estimates against any *Shigella* diarrhea from highly powered trials for all 50 simulation scenarios when using active surveillance for infection and a single outcome regression model.

Table Q. Mean estimate, bias, mean squared error (MSE), coverage, and false negative rate for vaccine efficacy (VE) estimates against any *Shigella* diarrhea from highly powered trials for all 50 simulation scenarios when using symptom-based reporting and a single outcome regression model.

Table R. Mean estimate, bias, mean squared error (MSE), coverage, and false negative rate for vaccine efficacy (VE) estimates against any *Shigella* diarrhea from highly powered trials for all 50 simulation scenarios when using active surveillance for infection and a stratified recurrent outcome regression model.

Table S. Mean estimate, bias, mean squared error (MSE), coverage, and false negative rate for vaccine efficacy (VE) estimates against any *Shigella* diarrhea from highly powered trials for all 50 simulation scenarios when using symptom-based reporting and a stratified recurrent outcome regression model.

Table T. Mean estimate, bias, mean squared error (MSE), coverage, and false negative rate for vaccine efficacy (VE) estimates against any *Shigella* diarrhea from highly powered trials for all 50 simulation scenarios when using active surveillance for infection and a crude recurrent outcome regression model.

Table U. Mean estimate, bias, mean squared error (MSE), coverage, and false negative rate for vaccine efficacy (VE) estimates against any *Shigella* diarrhea from highly powered trials for all 50 simulation scenarios when using symptom-based reporting and a crude recurrent outcome regression model.

Table V. Mean estimate, bias, mean squared error (MSE), coverage, and false negative rate for vaccine efficacy (VE) estimates against any *Shigella* diarrhea from realistically sized trials for all 50 simulation scenarios when using active surveillance for infection and a single outcome regression model.

Table W. Mean estimate, bias, mean squared error (MSE), coverage, and false negative rate for vaccine efficacy (VE) estimates against any *Shigella* diarrhea from realistically sized trials for all 50 simulation scenarios when using symptom-based reporting and a single outcome regression model.

Table X. Mean estimate, bias, mean squared error (MSE), coverage, and false negative rate for vaccine efficacy (VE) estimates against any *Shigella* diarrhea from realistically sized trials for all 50 simulation scenarios when using active surveillance for infection and a stratified recurrent outcome regression model.

Table Y. Mean estimate, bias, mean squared error (MSE), coverage, and false negative rate for vaccine efficacy (VE) estimates against any *Shigella* diarrhea from realistically sized trials for all 50 simulation scenarios when using symptom-based reporting and a stratified recurrent outcome regression model.

Table Z. Mean estimate, bias, mean squared error (MSE), coverage, and false negative rate for vaccine efficacy (VE) estimates against any *Shigella* diarrhea from realistically sized trials for all 50 simulation scenarios when using active surveillance for infection and a crude recurrent outcome regression model.

Table AA. Mean estimate, bias, mean squared error (MSE), coverage, and false negative rate for vaccine efficacy (VE) estimates against any *Shigella* diarrhea from realistically sized trials for all 50 simulation scenarios when using symptom-based reporting and a crude recurrent outcome regression model.

Table BB. Number of participants in realistically sized trials for all 50 simulation scenarios based on sample size calculations.

Figure A. Simulation structure and process for generating recurrent infections. Infections are simulated for one trial enrollee at a time. An enrollee's baseline infection hazard changes as they move through different age groups during the course of the trial, though this baseline infection hazard is constant for the entire time spent in a given age group. Baseline infection hazard is further modified by vaccine-conferred immunity (when applicable) and immunity accrued from infections that occur during follow-up.

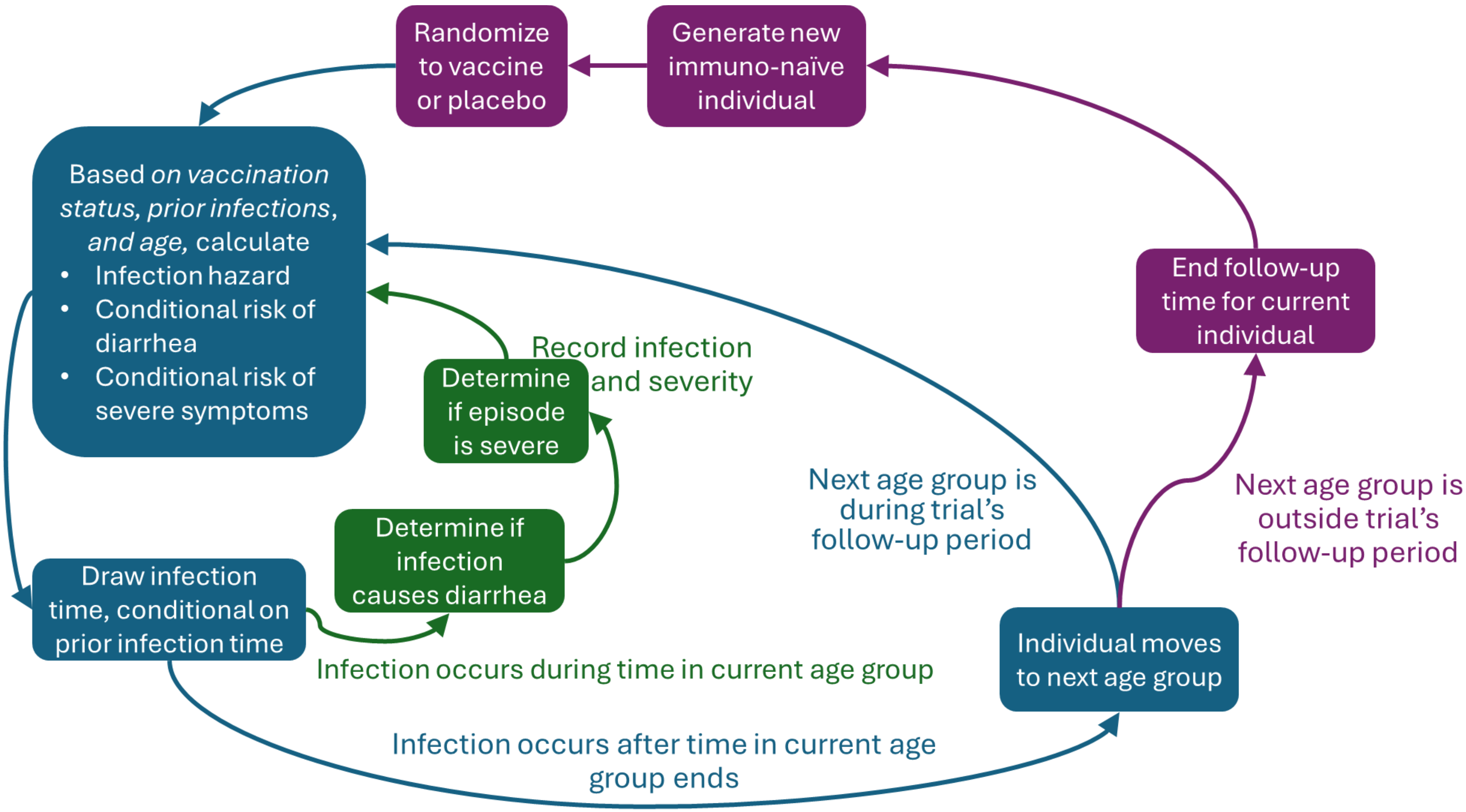

Each infant began the recurrent infection process at 10 months of age with no infection-acquired immunity. An infection hazard reflecting their vaccination status and age was calculated. This hazard was used to generate an infection day T_i from an exponential distribution conditioned on the starting age (in days) for the 10-12 month age group. If T_i occurred while the infant was still in the 10-12 month age group (i.e., through 365 days of age), they were infected. If not infected between 10 and 12 months of age, the hazard was updated to reflect the next age group and the infection generation process repeated.

Upon infection, the severity experienced was then determined with binomial draws using probabilities again calculated based on vaccination status and age. The first determined if they developed diarrhea; if so, a second draw determined if they had a severe outcome. The infection, age at infection (in days), and severity were recorded. For second and later infections, infection hazard and disease probabilities were modified to reflect infection-acquired immunity, the exponential distribution now conditioned on the age of the last infection, and another T_i generated. Once the generated T_i exceeded the end of the age group, the individual then entered the next age group with infection hazard and severity probabilities updated to reflect age and all relevant sources of immunity. This recurrent process continued through the last age group.

Equation A. Multiplier approach to generate an individual's infection hazard and disease progression probabilities based on their age, vaccination status, and infection history.

Among the larger population that the trial is being conducted in, let

h be the *Shigella* infection rate
d be the probability of developing symptoms, given a *Shigella* infection
s be the probability of experiencing severe disease, given a symptomatic *Shigella* infection

To allow for age-based trends in infection hazard, symptom development, and severe disease, let

$a = 1, 2, \dots, k$ be all the age groups that occur between the start and end ages for trial follow-up
rh_a be the hazard of *Shigella* infection for the kth age group within the larger population relative to the hazard in the referent age group
rd_a be the probability of developing symptoms, given a *Shigella* infection, for the kth age group within the larger population relative to the probability in the referent age group
rs_a be the probability of experiencing severe disease, given a symptomatic *Shigella* infection, for the kth age group within the larger population relative to the probability in the referent age group

Other factors that impact infection hazard and symptom and severity probabilities include

$i = 0, 1, 2, \dots$, the number of prior infections an individual has had that have conferred immunity
rh_i, the hazard of another *Shigella* infection after i previous infections relative to $i - 1$ previous infections
rd_i, the probability of developing symptoms, given a *Shigella* infection, after i previous infections relative to $i - 1$ previous infections
rs_i, the relative probability of experiencing severe disease, given a symptomatic *Shigella* infection, after i previous infections
$v = 0,1$, where 1 indicates the individual received the experimental *Shigella* vaccine and 0 indicates they did not
$1 - VE_h$, the relative hazard of another *Shigella* infection after vaccination compared to not being vaccinated
$1 - VE_d$, the relative probability of developing symptoms, given a *Shigella* infection, after vaccination compared to not being vaccinated
$1 - VE_s$, the relative probability of experiencing severe disease, given a symptomatic *Shigella* infection, after vaccination compared to not being vaccinated

An individual's infection hazard at a given time during trial follow-up is calculated as

$$h \times [(1 - VE_h)^v \times rh_i \times rh_a]$$

For example, in scenarios with hybrid immunity, similar infection- and vaccine-conferred immunity, and some vaccine-conferred protection against infection, a vaccinated and infected infant would have an infection hazard of $0.64(rh_a)h$. Probability of developing symptoms given infection and probability of experiencing severe disease, given symptomatic infection, are analogously calculated using the corresponding d- and s-based notation, respectively.

Figure B. Full set of simulation scenarios with different assumptions around vaccine effects, infection-acquired immunity, hybrid immunity, and the force of infection. When considering the maximum possible reductions to infection hazard and disease progression probabilities, unvaccinated infants can reach, but not exceed, the maximum possible immunity that vaccinated infants may accrue.

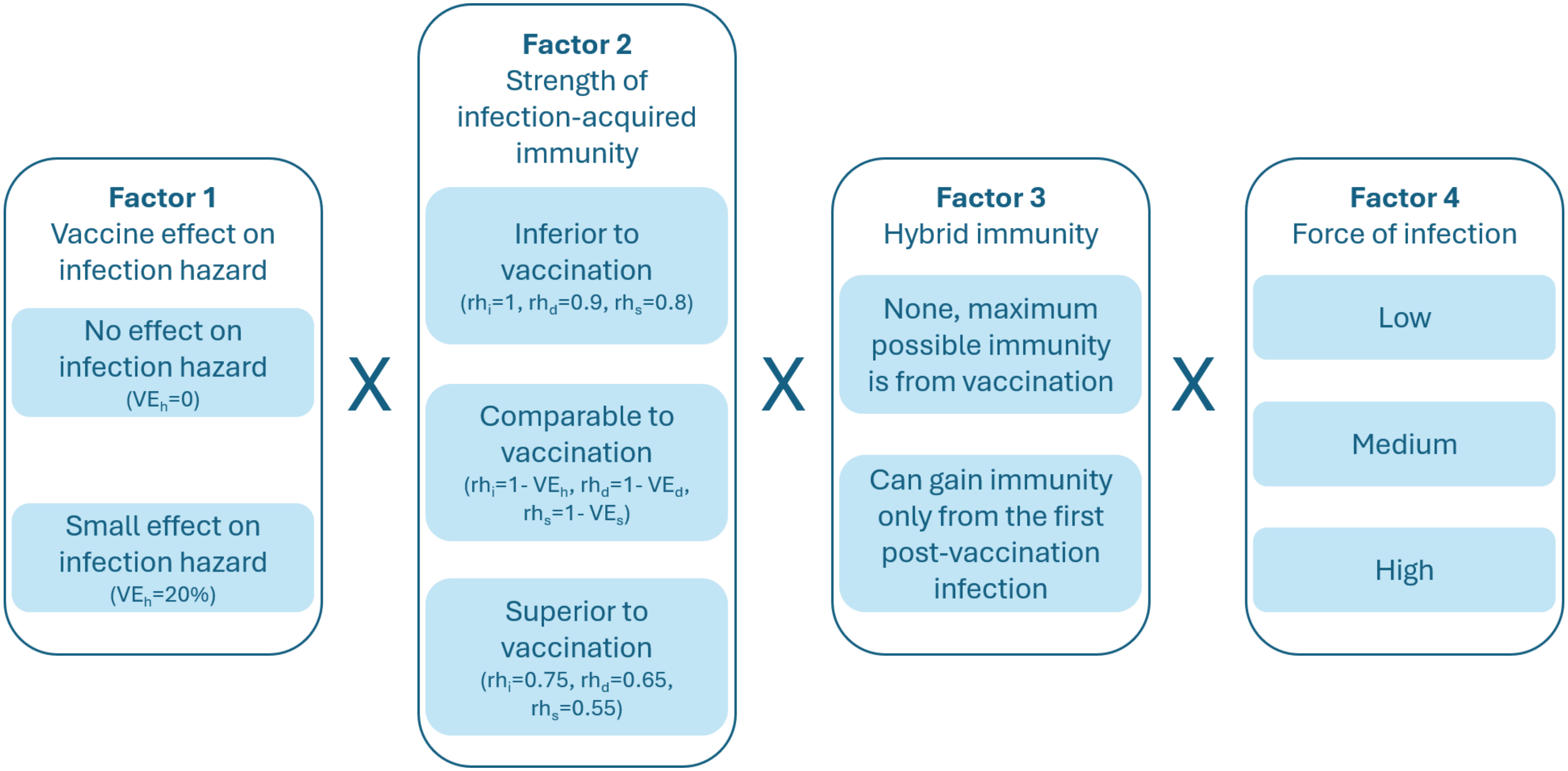

Table A. *Shigella* natural history parameters and infection incidence rates at low-, medium-, and high-burden sites. rh_a is the *Shigella* infection hazard for a given age group relative to the hazard in the referent age group. rd_a is the probability of developing symptoms, given a *Shigella* infection, for a given age group relative to the probability in the referent age group. rs_a is the probability of experiencing severe disease, given a symptomatic *Shigella* infection, for a given age group relative to the probability in the referent age group. Absolute hazards were 0.0007147541 infections/day for the low-burden site, 0.002254098 infections/day for the medium-burden site, and 0.003793443 infections/day for the high-burden site. Disease progression probabilities did not vary by site, with the probability of symptoms given infection equal to 0.35 and the probability of severe disease given symptoms equal to 0.084.

Age (months)	rh_a	rd_a	rs_a
10-12	0.539	1.029	1.488
13-15	0.669	1.114	1.845
16-18	0.860	1.114	1.845
19-21	1.000	1.000	1.000
22-24	0.920	1.000	1.000
25-30*	0.900	0.900	0.900
31-36*	0.800	0.800	0.800
*These ages were not part of the original study used to inform parameters. Based on the available evidence suggesting *Shigella* burden peaks in the 2nd year of life, we specified these parameters to represent decreases in hazard and disease progression probabilities.			

Table B. Summary of analytic approaches to evaluating ideal and realistic data generated from simulated clinical *Shigella* vaccine trials. Cox proportion hazards models were used for all regression models, with age as the underlying time scale and trial arm (placebo or vaccine) as a model covariate.

Analytic approach	Regression model type	Data collection approach
1	Single outcome<br>(censored after first infection)	Active surveillance for infection<br>(all infections ascertained)
2	Single outcome<br>(censored after first diarrheal episode)	Symptom-based reporting<br>(only diarrhea episodes ascertained)
3	Recurrent outcome<br>(strata based on prior infections)	Active surveillance for infection<br>(all infections ascertained)
4	Recurrent outcome<br>(strata based on prior diarrheal episodes)	Symptom-based reporting<br>(only diarrhea episodes ascertained)
5	Crude<br>(event history not considered)	Active surveillance for infection<br>(all infections ascertained)
6	Crude<br>(event history not considered)	Symptom-based reporting<br>(only diarrhea episodes ascertained)

Equation B. Calculating the number of simulations needed for target Monte Carlo standard errors.

Let

$MCSE$ be the desired maximum Monte Carlo standard error for a given performance measure

n_{sim} be the minimum number of simulations needed to achieve $MCSE$

$Var(\hat{\theta})$ be the variance of the vaccine efficacy (VE) estimate of primary interest (i.e., efficacy against severe *Shigella* diarrhea

$\mathrm{E}(Cov)$ be the expected 95% confidence interval coverage

For large trials, the primary performance measure of interest is bias. Our target $MCSE$ was 0.01 for VE prior to its transformation into a percentage (i.e., 0.6). Based on preliminary simulation runs with 10,000 participants, $Var(\hat{\theta})$ was ≤ 0.05. We determined that 500 runs would be sufficient using

$$n_{sim} = \frac{Var(\hat{\theta})}{MCSE^2}$$

After the initial 500 runs, we verified that $MCSE \leq 0.01$ for each main scenario.

For realistically sized trials, the primary performance measures of interest are coverage and false negative rate. Because both are percentages, the same formula is used to determine the number of simulation runs needed for a target $MCSE$ of 1% (coverage is used as an example here)

$$n_{sim} = \frac{\mathrm{E}(Cov) \times \left(1 - \mathrm{E}(Cov)\right)}{MCSE^2}$$

Under this formula, the number of simulations is maximized when $\mathrm{E}(Cov)$ is 50%, requiring 2,500 runs to achieve the desired $MCSE$.

Table C. Mean estimate, bias, mean squared error (MSE), coverage, and false negative rate for vaccine efficacy (VE) estimates against severe *Shigella* diarrhea from highly powered trials for all 50 simulation scenarios when using active surveillance for infection and a single outcome regression model. Infection-acquired immunity was either inferior or similar to vaccine-acquired immunity. Hybrid immunity was either not possible (no) or possible such that the first post-vaccination infection conferred additional immunity (yes). Bias was calculated as the average VE estimate for a set of simulations minus the true VE (60%). MSE was calculated as the average of the square of the estimate minus the true parameter. Coverage represents the percent of 95% confidence intervals (CIs) that contained the true VE (60%), and the false negative rate indicates the percent of 95% CIs that incorrectly indicated a non-significant effect.

VE against infection	Strength of infection-acquired immunity	Hybrid immunity	Force of infection	Mean VE estimate (range)	Bias (95% CI)	MSE (95% CI)	Coverage (95% CI)	False negatives (95% CI)
0%	Superior	Yes	Very Low	55.1% (-27.3%, 90.6%)	-4.9 (-6.5, -3.3)	360.1 (299.6, 420.5)	94.4% (92.4%, 96.4%)	41% (36.7%, 45.3%)
0%	Superior	Yes	Low	56.9% (15.3%, 82%)	-3.1 (-4, -2.3)	113.2 (95.8, 130.7)	92.2% (89.8%, 94.6%)	2.4% (1.1%, 3.7%)
0%	Superior	Yes	Medium	55.6% (22.8%, 73.8%)	-4.4 (-5.2, -3.6)	98 (83.4, 112.5)	92.6% (90.3%, 94.9%)	1.4% (0.4%, 2.4%)
0%	Superior	Yes	High	56.6% (35.9%, 75.8%)	-3.4 (-4, -2.9)	53.4 (46.1, 60.7)	92.2% (89.8%, 94.6%)	0% (0%, 0%)
0%	Superior	Yes	Very High	56.6% (41.2%, 68.1%)	-3.4 (-3.9, -3)	37.8 (33.2, 42.5)	93.2% (91%, 95.4%)	0% (0%, 0%)
0%	Similar	No	Very Low	58.5% (-25.9%, 93.6%)	-1.5 (-3, 0)	289.9 (236.6, 343.3)	96.4% (94.8%, 98%)	32.4% (28.3%, 36.5%)
0%	Similar	No	Low	58.8% (15%, 79.2%)	-1.2 (-2.1, -0.3)	98.8 (83.9, 113.7)	94.2% (92.2%, 96.2%)	1.4% (0.4%, 2.4%)
0%	Similar	No	Medium	59.1% (29.7%, 78.3%)	-0.9 (-1.6, -0.2)	65.7 (56.6, 74.9)	96.2% (94.5%, 97.9%)	0.2% (-0.2%, 0.6%)
0%	Similar	No	High	59.5% (43.7%, 74.9%)	-0.5 (-1, 0.1)	35.9 (31.7, 40.2)	95.4% (93.6%, 97.2%)	0% (0%, 0%)
0%	Similar	No	Very High	59.6% (43.3%, 73.2%)	-0.4 (-0.8, 0.1)	27.6 (24.2, 31)	95.4% (93.6%, 97.2%)	0% (0%, 0%)
0%	Similar	Yes	Very Low	58.8% (-25.8%, 93.3%)	-1.2 (-2.7, 0.2)	288.2 (238.9, 337.4)	96% (94.3%, 97.7%)	31.6% (27.5%, 35.7%)
0%	Similar	Yes	Low	59.2% (27.3%, 79.8%)	-0.8 (-1.6, 0)	89.8 (77.5, 102.1)	95.6% (93.8%, 97.4%)	1.6% (0.5%, 2.7%)
0%	Similar	Yes	Medium	58.5% (32.9%, 79.9%)	-1.5 (-2.2, -0.8)	70.4 (61, 79.8)	95% (93.1%, 96.9%)	0.2% (-0.2%, 0.6%)
0%	Similar	Yes	High	59.2% (37.2%, 73.4%)	-0.8 (-1.3, -0.2)	36.9 (32, 41.9)	95.8% (94%, 97.6%)	0% (0%, 0%)
0%	Similar	Yes	Very High	59.6% (38.7%, 73.7%)	-0.4 (-0.8, 0.1)	26.5 (22.9, 30.2)	95.8% (94%, 97.6%)	0% (0%, 0%)

	Inferior	No	Very Low	58.2% (-28.7%, 93.5%)	-1.8 (-3.3, -0.2)	307.2 (247.1, 367.3)	96.4% (94.8%, 98%)	31% (26.9%, 35.1%)
			Low	59.2% (27%, 81.2%)	-0.8 (-1.7, 0.1)	100.1 (85.9, 114.4)	92.4% (90.1%, 94.7%)	2.6% (1.2%, 4%)
			Medium	59.2% (16%, 82.2%)	-0.8 (-1.5, -0.1)	65.2 (54.2, 76.1)	96.4% (94.8%, 98%)	0.2% (-0.2%, 0.6%)
			High	59.5% (38.1%, 75.2%)	-0.5 (-1, 0.1)	38.7 (33.5, 43.9)	95.6% (93.8%, 97.4%)	0% (0%, 0%)
			Very High	59.9% (40%, 74.1%)	-0.1 (-0.6, 0.4)	29.7 (25.8, 33.6)	95.8% (94%, 97.6%)	0% (0%, 0%)
		Yes	Very Low	58.3% (-28.7%, 92.8%)	-1.7 (-3.3, -0.2)	300.1 (241.9, 358.3)	96.4% (94.8%, 98%)	32% (27.9%, 36.1%)
			Low	59.4% (25.2%, 82.4%)	-0.6 (-1.5, 0.2)	94.3 (80.5, 108.1)	93.4% (91.2%, 95.6%)	1.6% (0.5%, 2.7%)
			Medium	59.1% (14.5%, 80.8%)	-0.9 (-1.6, -0.1)	67.4 (55.7, 79.1)	95.6% (93.8%, 97.4%)	0.4% (-0.2%, 1%)
			High	59.4% (38.1%, 73.5%)	-0.6 (-1.2, -0.1)	38.5 (33.5, 43.5)	95.4% (93.6%, 97.2%)	0% (0%, 0%)
			Very High	59.8% (38.3%, 74.1%)	-0.2 (-0.6, 0.3)	28.8 (24.9, 32.7)	96% (94.3%, 97.7%)	0% (0%, 0%)
20%	Superior	Yes	Very Low	57.9% (-22.2%, 93.4%)	-2.1 (-3.6, -0.6)	299.5 (254, 344.9)	97.4% (96%, 98.8%)	35.2% (31%, 39.4%)
			Low	58.9% (31.1%, 83.7%)	-1.1 (-1.9, -0.3)	80.8 (70.2, 91.3)	95.6% (93.8%, 97.4%)	1% (0.1%, 1.9%)
			Medium	59.7% (35.9%, 79.8%)	-0.3 (-1, 0.4)	63.9 (56.8, 71)	97.2% (95.8%, 98.6%)	0% (0%, 0%)
			High	59.7% (41.8%, 73.3%)	-0.3 (-0.8, 0.2)	28.4 (24.8, 32.1)	97% (95.5%, 98.5%)	0% (0%, 0%)
			Very High	60% (40.8%, 73.3%)	0 (-0.5, 0.5)	28.2 (24.7, 31.6)	95.6% (93.8%, 97.4%)	0% (0%, 0%)
	Similar	No	Very Low	57.7% (-22.3%, 91.4%)	-2.3 (-3.9, -0.7)	329 (267.6, 390.5)	95.2% (93.3%, 97.1%)	34.8% (30.6%, 39%)
			Low	59.2% (9.5%, 82.3%)	-0.8 (-1.6, 0)	86 (71.7, 100.4)	94.6% (92.6%, 96.6%)	0.6% (-0.1%, 1.3%)
			Medium	59.7% (20.5%, 81.6%)	-0.3 (-1, 0.4)	66.1 (56.2, 76.1)	96% (94.3%, 97.7%)	0.2% (-0.2%, 0.6%)
			High	59.5% (41.7%, 75.9%)	-0.5 (-1, 0)	35.8 (31.5, 40.1)	95.2% (93.3%, 97.1%)	0% (0%, 0%)

			Very High	60.1%<br>(37.1%, 73.4%)	0.1<br>(-0.4, 0.6)	30.7<br>(26.4, 35)	92.4%<br>(90.1%, 94.7%)	0%<br>(0%, 0%)
		Yes	Very Low	58.3%<br>(-32.4%, 90.4%)	-1.7<br>(-3.3, -0.2)	312.1<br>(254.7, 369.4)	96.6%<br>(95%, 98.2%)	33.2%<br>(29.1%, 37.3%)
			Low	59.1%<br>(23.9%, 85.3%)	-0.9<br>(-1.7, -0.1)	88.6<br>(75.6, 101.5)	95.2%<br>(93.3%, 97.1%)	1.6%<br>(0.5%, 2.7%)
			Medium	59.9%<br>(24.1%, 82.7%)	-0.1<br>(-0.8, 0.7)	72.6<br>(61.7, 83.4)	94.6%<br>(92.6%, 96.6%)	0.4%<br>(-0.2%, 1%)
			High	59.8%<br>(43.4%, 74.9%)	-0.2<br>(-0.7, 0.2)	27.6<br>(24.1, 31.1)	97.4%<br>(96%, 98.8%)	0%<br>(0%, 0%)
			Very High	59.7%<br>(38.2%, 74.7%)	-0.3<br>(-0.8, 0.1)	26.2<br>(22.3, 30)	96.4%<br>(94.8%, 98%)	0%<br>(0%, 0%)
	Inferior	No	Very Low	57.8%<br>(-28.4%, 91.4%)	-2.2<br>(-3.8, -0.7)	301.9<br>(245.1, 358.7)	95.8%<br>(94%, 97.6%)	32%<br>(27.9%, 36.1%)
			Low	58.9%<br>(21.6%, 82.3%)	-1.1<br>(-2, -0.3)	91.2<br>(78.1, 104.3)	94%<br>(91.9%, 96.1%)	1.2%<br>(0.2%, 2.2%)
			Medium	59.3%<br>(27.4%, 80.8%)	-0.7<br>(-1.4, 0.1)	71.5<br>(61.6, 81.3)	94%<br>(91.9%, 96.1%)	0.2%<br>(-0.2%, 0.6%)
			High	59.6%<br>(40.8%, 73.5%)	-0.4<br>(-0.9, 0.1)	32.9<br>(28.5, 37.3)	95.2%<br>(93.3%, 97.1%)	0%<br>(0%, 0%)
			Very High	60.1%<br>(45%, 75.7%)	0.1<br>(-0.3, 0.6)	23.7<br>(20.7, 26.8)	96.2%<br>(94.5%, 97.9%)	0%<br>(0%, 0%)
		Yes	Very Low	57.7%<br>(-28.4%, 92.4%)	-2.3<br>(-3.8, -0.8)	305.9<br>(249, 362.7)	95.2%<br>(93.3%, 97.1%)	32%<br>(27.9%, 36.1%)
			Low	58.9%<br>(25.7%, 86.3%)	-1.1<br>(-1.9, -0.2)	92.2<br>(79.1, 105.3)	94.2%<br>(92.2%, 96.2%)	1.2%<br>(0.2%, 2.2%)
			Medium	59.4%<br>(25.4%, 81%)	-0.6<br>(-1.3, 0.1)	71.4<br>(60.4, 82.3)	93.2%<br>(91%, 95.4%)	0.4%<br>(-0.2%, 1%)
			High	59.5%<br>(39.1%, 73.3%)	-0.5<br>(-1, 0)	33.2<br>(28.7, 37.8)	96.2%<br>(94.5%, 97.9%)	0%<br>(0%, 0%)
			Very High	60.3%<br>(42.8%, 74%)	0.3<br>(-0.1, 0.8)	25.4<br>(22.3, 28.5)	94.8%<br>(92.9%, 96.7%)	0%<br>(0%, 0%)

Table D. Mean estimate, bias, mean squared error (MSE), coverage, and false negative rate for vaccine efficacy (VE) estimates against severe *Shigella* diarrhea from highly powered trials for all 50 simulation scenarios when using symptom-based reporting and a single outcome regression model. Infection-acquired immunity was either inferior or similar to vaccine-acquired immunity. Hybrid immunity was either not possible (no) or possible such that the first post-vaccination infection conferred additional immunity (yes). Bias was calculated as the average VE estimate for a set of simulations minus the true VE (60%). MSE was calculated as the average of the square of the estimate minus the true parameter. Coverage represents the percent of 95% confidence intervals (CIs) that contained the true VE (60%), and the false negative rate indicates the percent of 95% CIs that incorrectly indicated a non-significant effect.

VE against infection	Strength of infection-acquired immunity	Hybrid immunity	Force of infection	Mean VE estimate (range)	Bias (95% CI)	MSE (95% CI)	Coverage (95% CI)	False negatives (95% CI)
0%	Superior	Yes	Very Low	54.8% (-26.9%, 88.1%)	-5.2 (-6.8, -3.6)	359.3 (299.1, 419.5)	94.4% (92.4%, 96.4%)	42% (37.7%, 46.3%)
			Low	55.3% (17.8%, 81.2%)	-4.7 (-5.6, -3.8)	129 (110.1, 147.9)	89.8% (87.1%, 92.5%)	2.4% (1.1%, 3.7%)
			Medium	53.9% (21.8%, 72.3%)	-6.1 (-6.9, -5.3)	119.4 (101.8, 137)	90.4% (87.8%, 93%)	1.6% (0.5%, 2.7%)
			High	52.2% (30.9%, 71%)	-7.8 (-8.4, -7.3)	103.2 (92.2, 114.3)	72.8% (68.9%, 76.7%)	0% (0%, 0%)
			Very High	49.7% (34.9%, 62.5%)	-10.3 (-10.7, -9.8)	133.4 (123.2, 143.6)	45.4% (41%, 49.8%)	0% (0%, 0%)
	Similar	No	Very Low	57.8% (-26%, 93.6%)	-2.2 (-3.7, -0.8)	291.3 (238.7, 343.8)	96% (94.3%, 97.7%)	34.4% (30.2%, 38.6%)
			Low	56% (21.4%, 76.4%)	-4 (-4.9, -3.1)	119.5 (102.2, 136.9)	92.6% (90.3%, 94.9%)	2.6% (1.2%, 4%)
			Medium	55.6% (29.3%, 77.1%)	-4.4 (-5.1, -3.6)	89.3 (76.2, 102.4)	93.4% (91.2%, 95.6%)	0.4% (-0.2%, 1%)
			High	51.7% (32.8%, 68.2%)	-8.3 (-8.8, -7.8)	104.4 (94.3, 114.6)	70.2% (66.2%, 74.2%)	0% (0%, 0%)
			Very High	47.4% (29%, 63.2%)	-12.6 (-13.1, -12.1)	190.2 (177.2, 203.2)	28.6% (24.6%, 32.6%)	0% (0%, 0%)
		Yes	Very Low	59% (-26%, 93.4%)	-1 (-2.5, 0.5)	280.1 (231.7, 328.5)	95.8% (94%, 97.6%)	31.2% (27.1%, 35.3%)
			Low	59.6% (23.3%, 80.8%)	-0.4 (-1.2, 0.4)	84.8 (73.2, 96.4)	94.8% (92.9%, 96.7%)	0.8% (0%, 1.6%)
			Medium	59.2% (35.8%, 80.4%)	-0.8 (-1.5, -0.1)	63.9 (55.6, 72.3)	94.8% (92.9%, 96.7%)	0% (0%, 0%)
			High	59.7% (41.4%, 72.5%)	-0.3 (-0.8, 0.2)	30.3 (26.5, 34)	95.8% (94%, 97.6%)	0% (0%, 0%)
			Very High	58.9% (44.4%, 71.7%)	-1.1 (-1.5, -0.7)	21.8 (18.8, 24.8)	94.4% (92.4%, 96.4%)	0% (0%, 0%)

	Inferior	No	Very Low	57.9% (-29.3%, 93.8%)	-2.1 (-3.6, -0.5)	304.4 (244.3, 364.6)	95.6% (93.8%, 97.4%)	30.8% (26.8%, 34.8%)
			Low	58% (22.8%, 80.2%)	-2 (-2.8, -1.1)	100.5 (85.5, 115.4)	91.6% (89.2%, 94%)	2% (0.8%, 3.2%)
			Medium	57.5% (19.8%, 78.9%)	-2.5 (-3.2, -1.8)	68.6 (57.8, 79.4)	94.8% (92.9%, 96.7%)	0.2% (-0.2%, 0.6%)
			High	55.8% (37.9%, 71.9%)	-4.2 (-4.7, -3.7)	47.8 (42.1, 53.5)	89% (86.3%, 91.7%)	0% (0%, 0%)
			Very High	53.5% (37.3%, 65.9%)	-6.5 (-6.9, -6)	64.5 (57.7, 71.2)	73% (69.1%, 76.9%)	0% (0%, 0%)
		Yes	Very Low	58.5% (-26%, 93.2%)	-1.5 (-3, 0)	287.6 (234.1, 341)	96% (94.3%, 97.7%)	31.4% (27.3%, 35.5%)
			Low	59.6% (23%, 80.9%)	-0.4 (-1.2, 0.4)	87.1 (74.6, 99.5)	92.6% (90.3%, 94.9%)	1% (0.1%, 1.9%)
			Medium	59.4% (19.2%, 80.2%)	-0.6 (-1.3, 0)	61.5 (51.5, 71.6)	94.4% (92.4%, 96.4%)	0.2% (-0.2%, 0.6%)
			High	59.5% (43.3%, 73.3%)	-0.5 (-0.9, 0)	27.2 (23.9, 30.5)	96.4% (94.8%, 98%)	0% (0%, 0%)
			Very High	58.9% (40.4%, 70.2%)	-1.1 (-1.5, -0.7)	20.2 (17.4, 23.1)	96.2% (94.5%, 97.9%)	0% (0%, 0%)
20%	Superior	Yes	Very Low	58.9% (40.4%, 70.2%)	-1.1 (-1.5, -0.7)	20.2 (17.4, 23.1)	96.2% (94.5%, 97.9%)	0% (0%, 0%)
			Low	57.4% (-22.6%, 93.4%)	-2.6 (-4.1, -1.1)	297.9 (251.7, 344.2)	97.4% (96%, 98.8%)	35.4% (31.2%, 39.6%)
			Medium	57.4% (28.6%, 79.8%)	-2.6 (-3.4, -1.8)	88.1 (76.7, 99.5)	95.8% (94%, 97.6%)	0.8% (0%, 1.6%)
			High	57.8% (34.6%, 78.2%)	-2.2 (-2.9, -1.5)	70.2 (61.7, 78.8)	95.4% (93.6%, 97.2%)	0% (0%, 0%)
			Very High	55.1% (36.4%, 70.2%)	-4.9 (-5.4, -4.4)	52.9 (46.2, 59.6)	90.6% (88%, 93.2%)	0% (0%, 0%)
	Similar	No	Very Low	52.6% (29.5%, 67.8%)	-7.4 (-7.9, -6.9)	86 (77, 95)	68.2% (64.1%, 72.3%)	0% (0%, 0%)
			Low	56.6% (16.2%, 80.2%)	-3.4 (-4.2, -2.6)	98.7 (84, 113.3)	93.6% (91.5%, 95.7%)	1.4% (0.4%, 2.4%)
			Medium	56.2% (19.9%, 82.4%)	-3.8 (-4.5, -3)	83.9 (72.3, 95.6)	93.6% (91.5%, 95.7%)	0.2% (-0.2%, 0.6%)
			High	51.6% (27.1%, 67.6%)	-8.4 (-9, -7.9)	112.5 (100.5, 124.5)	69.6% (65.6%, 73.6%)	0% (0%, 0%)

			Very High	47.8% (30.9%, 62.1%)	-12.2 (-12.7, -11.7)	184.4 (170.6, 198.2)	31.2% (27.1%, 35.3%)	0% (0%, 0%)
		Yes	Very Low	58.1% (-32.8%, 90.4%)	-1.9 (-3.4, -0.3)	312.7 (254.8, 370.7)	96.2% (94.5%, 97.9%)	33.4% (29.3%, 37.5%)
			Low	58.8% (23%, 84.2%)	-1.2 (-2, -0.4)	85.2 (73, 97.5)	95.2% (93.3%, 97.1%)	1.2% (0.2%, 2.2%)
			Medium	59.5% (27.7%, 80.3%)	-0.5 (-1.2, 0.2)	68.3 (58.7, 77.9)	95.6% (93.8%, 97.4%)	0.2% (-0.2%, 0.6%)
			High	58.5% (41.6%, 73.7%)	-1.5 (-2, -1.1)	29.3 (25.4, 33.1)	95.8% (94%, 97.6%)	0% (0%, 0%)
			Very High	57% (38.2%, 70.3%)	-3 (-3.5, -2.6)	34.8 (29.9, 39.7)	90% (87.4%, 92.6%)	0% (0%, 0%)
	Inferior	No	Very Low	57.3% (-15.8%, 92.1%)	-2.7 (-4.2, -1.2)	295.5 (242.2, 348.8)	95.6% (93.8%, 97.4%)	31.4% (27.3%, 35.5%)
			Low	57.6% (23.5%, 80.1%)	-2.4 (-3.2, -1.5)	92.2 (79.2, 105.3)	94.4% (92.4%, 96.4%)	1% (0.1%, 1.9%)
			Medium	57.7% (24.5%, 79.7%)	-2.3 (-3, -1.5)	75.1 (64.1, 86)	94.4% (92.4%, 96.4%)	0.4% (-0.2%, 1%)
			High	55.9% (37%, 71.4%)	-4.1 (-4.6, -3.6)	46.4 (40.4, 52.3)	89% (86.3%, 91.7%)	0% (0%, 0%)
			Very High	54% (39.5%, 69.5%)	-6 (-6.4, -5.6)	56.8 (51.2, 62.5)	75.4% (71.6%, 79.2%)	0% (0%, 0%)
		Yes	Very Low	57.6% (-13.4%, 92.6%)	-2.4 (-3.9, -0.9)	295.6 (244.7, 346.6)	95.4% (93.6%, 97.2%)	31.6% (27.5%, 35.7%)
			Low	58.9% (22.7%, 81.9%)	-1.1 (-1.9, -0.3)	85.8 (73.6, 97.9)	94.2% (92.2%, 96.2%)	0.8% (0%, 1.6%)
			Medium	59.1% (29.1%, 80.1%)	-0.9 (-1.6, -0.2)	67.5 (57.7, 77.3)	93.4% (91.2%, 95.6%)	0.4% (-0.2%, 1%)
			High	58.9% (42%, 73.1%)	-1.1 (-1.5, -0.6)	28.3 (24.6, 32)	95.4% (93.6%, 97.2%)	0% (0%, 0%)
			Very High	58.2% (40.4%, 70.1%)	-1.8 (-2.2, -1.4)	23.5 (20.2, 26.8)	93.4% (91.2%, 95.6%)	0% (0%, 0%)

Table E. Mean estimate, bias, mean squared error (MSE), coverage, and false negative rate for vaccine efficacy (VE) estimates against severe *Shigella* diarrhea from highly powered trials for all 50 simulation scenarios when using active surveillance for infection and a stratified recurrent outcome regression model. Hybrid immunity was either not possible (no) or possible such that the first post-vaccination infection conferred additional immunity (yes). Bias was calculated as the average VE estimate for a set of simulations minus the true VE (60%). MSE was calculated as the average of the square of the estimate minus the true parameter. Coverage represents the percent of 95% confidence intervals (CIs) that contained the true VE (60%), and the false negative rate indicates the percent of 95% CIs that incorrectly indicated a non-significant effect.

VE against infection	Strength of infection-acquired immunity	Hybrid immunity	Force of infection	Mean VE estimate (range)	Bias (95% CI)	MSE (95% CI)	Coverage (95% CI)	False negatives (95% CI)
0%	Superior	Yes	Very Low	54.5% (-27.3%, 88%)	-5.5 (-7.2, -3.9)	366.6 (305.4, 427.8)	93.8% (91.7%, 95.9%)	42.4% (38.1%, 46.7%)
			Low	54% (20.5%, 81.1%)	-6 (-6.9, -5)	146.2 (125.4, 167)	88% (85.2%, 90.8%)	3.4% (1.8%, 5%)
			Medium	52.4% (18.2%, 71.5%)	-7.6 (-8.4, -6.8)	139.6 (119.8, 159.4)	86.4% (83.4%, 89.4%)	1.8% (0.6%, 3%)
			High	48.5% (27.2%, 66.3%)	-11.5 (-12.1, -10.9)	176.8 (161.4, 192.3)	52.2% (47.8%, 56.6%)	0% (0%, 0%)
			Very High	44.4% (26%, 58.6%)	-15.6 (-16.1, -15.2)	275.5 (259.3, 291.7)	14% (11%, 17%)	0% (0%, 0%)
	Similar	No	Very Low	57.2% (-25.9%, 93.7%)	-2.8 (-4.3, -1.3)	301.9 (247.5, 356.2)	96% (94.3%, 97.7%)	35% (30.8%, 39.2%)
			Low	54.5% (18.9%, 75.9%)	-5.5 (-6.4, -4.6)	134.5 (115.2, 153.8)	90.4% (87.8%, 93%)	2.8% (1.4%, 4.2%)
			Medium	53.7% (25.7%, 75.8%)	-6.3 (-7, -5.5)	110.7 (95.5, 125.9)	89.4% (86.7%, 92.1%)	1% (0.1%, 1.9%)
			High	47.3% (30.1%, 63.3%)	-12.7 (-13.2, -12.2)	196.1 (182, 210.2)	38.6% (34.3%, 42.9%)	0% (0%, 0%)
			Very High	40.4% (18.2%, 57.2%)	-19.6 (-20.1, -19.1)	416.4 (395.7, 437.2)	2.4% (1.1%, 3.7%)	0% (0%, 0%)
		Yes	Very Low	58.9% (-25.8%, 93.3%)	-1.1 (-2.6, 0.3)	280 (231.6, 328.5)	96% (94.3%, 97.7%)	31.8% (27.7%, 35.9%)
			Low	59% (25.6%, 79.7%)	-1 (-1.8, -0.2)	83.3 (72.2, 94.5)	96% (94.3%, 97.7%)	0.8% (0%, 1.6%)
			Medium	58.4% (33.6%, 79.5%)	-1.6 (-2.3, -0.9)	65.1 (56.3, 74)	94.8% (92.9%, 96.7%)	0% (0%, 0%)
			High	57.7% (39.7%, 70.8%)	-2.3 (-2.8, -1.8)	35.2 (30.7, 39.7)	94.2% (92.2%, 96.2%)	0% (0%, 0%)
			Very High	55.4% (38.4%, 66.7%)	-4.6 (-5, -4.2)	44.6 (39, 50.3)	84.4% (81.2%, 87.6%)	0% (0%, 0%)

	Inferior	No	Very Low	57.7% (-27.3%, 93.9%)	-2.3 (-3.8, -0.8)	306.4 (244.5, 368.3)	95.8% (94%, 97.6%)	30.8% (26.8%, 34.8%)
			Low	57.2% (25.3%, 80.5%)	-2.8 (-3.6, -1.9)	106 (90.2, 121.8)	91.4% (88.9%, 93.9%)	2.2% (0.9%, 3.5%)
			Medium	56.5% (13.2%, 77.5%)	-3.5 (-4.2, -2.9)	74.2 (61.6, 86.8)	93.8% (91.7%, 95.9%)	0.2% (-0.2%, 0.6%)
			High	53.3% (36%, 66.8%)	-6.7 (-7.1, -6.2)	73.2 (65.7, 80.8)	77.4% (73.7%, 81.1%)	0% (0%, 0%)
			Very High	49.2% (32.8%, 60.7%)	-10.8 (-11.2, -10.4)	140.2 (130, 150.4)	28.8% (24.8%, 32.8%)	0% (0%, 0%)
		Yes	Very Low	58.4% (-26.8%, 93.3%)	-1.6 (-3.1, -0.1)	285.1 (230.4, 339.9)	96% (94.3%, 97.7%)	30.8% (26.8%, 34.8%)
			Low	59.2% (23.9%, 80.8%)	-0.8 (-1.7, 0)	88.1 (75.6, 100.6)	93.2% (91%, 95.4%)	0.8% (0%, 1.6%)
			Medium	58.8% (15.1%, 78%)	-1.2 (-1.9, -0.5)	61.2 (50.1, 72.3)	95.2% (93.3%, 97.1%)	0.2% (-0.2%, 0.6%)
			High	58.1% (41.9%, 72.3%)	-1.9 (-2.3, -1.5)	28.5 (24.9, 32.2)	94.8% (92.9%, 96.7%)	0% (0%, 0%)
			Very High	56.2% (40.7%, 66.3%)	-3.8 (-4.2, -3.5)	33.2 (28.9, 37.4)	85.8% (82.7%, 88.9%)	0% (0%, 0%)
20%	Superior	Yes	Very Low	57.4% (-22.2%, 93.6%)	-2.6 (-4.1, -1.1)	293.3 (248, 338.6)	97.8% (96.5%, 99.1%)	35% (30.8%, 39.2%)
			Low	57.1% (28.2%, 78.9%)	-2.9 (-3.7, -2.1)	89.1 (77.6, 100.5)	95.2% (93.3%, 97.1%)	0.6% (-0.1%, 1.3%)
			Medium	57.5% (35.4%, 78.6%)	-2.5 (-3.2, -1.7)	72.7 (63.6, 81.9)	94.4% (92.4%, 96.4%)	0% (0%, 0%)
			High	54.3% (33.4%, 70.5%)	-5.7 (-6.1, -5.2)	61.2 (53.8, 68.7)	86.8% (83.8%, 89.8%)	0% (0%, 0%)
			Very High	51% (27.6%, 65.8%)	-9 (-9.4, -8.5)	110.2 (100.1, 120.2)	56% (51.6%, 60.4%)	0% (0%, 0%)
	Similar	No	Very Low	56.5% (-22.3%, 91.7%)	-3.5 (-5.1, -1.9)	345.2 (280.6, 409.8)	94.6% (92.6%, 96.6%)	36.4% (32.2%, 40.6%)
			Low	55.7% (18.4%, 79.8%)	-4.3 (-5.1, -3.5)	104 (89.3, 118.8)	92.6% (90.3%, 94.9%)	1.4% (0.4%, 2.4%)
			Medium	54.9% (19.1%, 82.6%)	-5.1 (-5.8, -4.3)	96.4 (83.6, 109.2)	91% (88.5%, 93.5%)	0.2% (-0.2%, 0.6%)
			High	48.6% (26.4%, 66.8%)	-11.4 (-12, -10.8)	171.9 (156.7, 187.1)	49.6% (45.2%, 54%)	0% (0%, 0%)

			Very High	42.8%<br>(25%, 60.9%)	-17.2<br>(-17.7, -16.6)	329.7<br>(310.8, 348.7)	8.4%<br>(6%, 10.8%)	0%<br>(0%, 0%)
		Yes	Very Low	58.3%<br>(-32.4%, 90.4%)	-1.7<br>(-3.3, -0.2)	310.8<br>(253.2, 368.3)	96.4%<br>(94.8%, 98%)	32.8%<br>(28.7%, 36.9%)
			Low	59%<br>(26.4%, 84.6%)	-1<br>(-1.8, -0.2)	81.6<br>(70.1, 93.2)	95.8%<br>(94%, 97.6%)	0.8%<br>(0%, 1.6%)
			Medium	59.8%<br>(29.8%, 80.7%)	-0.2<br>(-0.9, 0.5)	65.9<br>(56.6, 75.2)	95.2%<br>(93.3%, 97.1%)	0.4%<br>(-0.2%, 1%)
			High	58.8%<br>(41.4%, 73.2%)	-1.2<br>(-1.7, -0.8)	27.1<br>(23.4, 30.9)	96.4%<br>(94.8%, 98%)	0%<br>(0%, 0%)
			Very High	57%<br>(41%, 70.9%)	-3<br>(-3.5, -2.6)	33.4<br>(28.8, 37.9)	89.8%<br>(87.1%, 92.5%)	0%<br>(0%, 0%)
	Inferior	No	Very Low	57.2%<br>(-18.1%, 92.2%)	-2.8<br>(-4.3, -1.3)	297.8<br>(243.5, 352.1)	95%<br>(93.1%, 96.9%)	30%<br>(26%, 34%)
			Low	57.1%<br>(25.2%, 80.8%)	-2.9<br>(-3.7, -2.1)	89.7<br>(77.2, 102.2)	94%<br>(91.9%, 96.1%)	1%<br>(0.1%, 1.9%)
			Medium	57.2%<br>(28.6%, 79%)	-2.8<br>(-3.6, -2.1)	75.3<br>(64.6, 86)	92.8%<br>(90.5%, 95.1%)	0.2%<br>(-0.2%, 0.6%)
			High	54.3%<br>(35.1%, 69.3%)	-5.7<br>(-6.1, -5.2)	60.4<br>(53.4, 67.4)	82.2%<br>(78.8%, 85.6%)	0%<br>(0%, 0%)
			Very High	51%<br>(36.1%, 65.9%)	-9<br>(-9.4, -8.6)	100.8<br>(92.8, 108.8)	46.2%<br>(41.8%, 50.6%)	0%<br>(0%, 0%)
		Yes	Very Low	57.8%<br>(-12.9%, 92.6%)	-2.2<br>(-3.7, -0.7)	292.4<br>(241.3, 343.5)	95.2%<br>(93.3%, 97.1%)	30.2%<br>(26.2%, 34.2%)
			Low	58.9%<br>(28.8%, 81.9%)	-1.1<br>(-1.9, -0.3)	79<br>(68.8, 89.3)	95.6%<br>(93.8%, 97.4%)	0.8%<br>(0%, 1.6%)
			Medium	59.2%<br>(32.2%, 79.4%)	-0.8<br>(-1.5, -0.1)	63<br>(54.4, 71.6)	93.6%<br>(91.5%, 95.7%)	0%<br>(0%, 0%)
			High	58.6%<br>(39.1%, 73.3%)	-1.4<br>(-1.9, -1)	27.5<br>(23.8, 31.3)	94.6%<br>(92.6%, 96.6%)	0%<br>(0%, 0%)
			Very High	57.2%<br>(40.4%, 70.2%)	-2.8<br>(-3.2, -2.5)	27<br>(23.3, 30.7)	90%<br>(87.4%, 92.6%)	0%<br>(0%, 0%)

Table F. Mean estimate, bias, mean squared error (MSE), coverage, and false negative rate for vaccine efficacy (VE) estimates against severe *Shigella* diarrhea from highly powered trials for all 50 simulation scenarios when using symptom-based reporting and a stratified recurrent outcome regression model. Hybrid immunity was either not possible (no) or possible such that the first post-vaccination infection conferred additional immunity (yes). Bias was calculated as the average VE estimate for a set of simulations minus the true VE (60%). MSE was calculated as the average of the square of the estimate minus the true parameter. Coverage represents the percent of 95% confidence intervals (CIs) that contained the true VE (60%), and the false negative rate indicates the percent of 95% CIs that incorrectly indicated a non-significant effect.

VE against infection	Strength of infection-acquired immunity	Hybrid immunity	Force of infection	Mean VE estimate (range)	Bias (95% CI)	MSE (95% CI)	Coverage (95% CI)	False negatives (95% CI)
0%	Superior	Yes	Very Low	54.6% (-26.9%, 88.1%)	-5.4 (-7, -3.8)	361.7 (301.7, 421.8)	94% (91.9%, 96.1%)	41.8% (37.5%, 46.1%)
			Low	54.6% (20.4%, 81.4%)	-5.4 (-6.3, -4.5)	137.6 (117.8, 157.4)	88.8% (86%, 91.6%)	3.2% (1.7%, 4.7%)
			Medium	53.1% (19.4%, 72.3%)	-6.9 (-7.6, -6.1)	127.6 (109, 146.2)	87.6% (84.7%, 90.5%)	1.6% (0.5%, 2.7%)
			High	49.9% (28.8%, 67.5%)	-10.1 (-10.7, -9.6)	146.1 (132.6, 159.7)	60.6% (56.3%, 64.9%)	0% (0%, 0%)
			Very High	46.1% (28%, 60%)	-13.9 (-14.4, -13.5)	223.5 (209.3, 237.8)	21.2% (17.6%, 24.8%)	0% (0%, 0%)
	Similar	No	Very Low	57.3% (-26%, 93.7%)	-2.7 (-4.3, -1.2)	301.2 (246.6, 355.8)	96% (94.3%, 97.7%)	35.6% (31.4%, 39.8%)
			Low	54.9% (19.8%, 76%)	-5.1 (-6, -4.2)	129.7 (110.8, 148.5)	91.4% (88.9%, 93.9%)	2.6% (1.2%, 4%)
			Medium	54.3% (25.8%, 76.4%)	-5.7 (-6.5, -5)	103 (88.6, 117.4)	90.8% (88.3%, 93.3%)	1% (0.1%, 1.9%)
			High	48.3% (31.1%, 63.8%)	-11.7 (-12.2, -11.2)	171.2 (158.2, 184.2)	47% (42.6%, 51.4%)	0% (0%, 0%)
			Very High	41.6% (20.5%, 58.4%)	-18.4 (-18.9, -17.9)	371.1 (351.7, 390.5)	4.4% (2.6%, 6.2%)	0% (0%, 0%)
		Yes	Very Low	59% (-26%, 93.4%)	-1 (-2.4, 0.5)	276.8 (228.8, 324.8)	96% (94.3%, 97.7%)	31.6% (27.5%, 35.7%)
			Low	59.6% (26.3%, 79.9%)	-0.4 (-1.2, 0.4)	80.6 (70, 91.1)	95.8% (94%, 97.6%)	0.8% (0%, 1.6%)
			Medium	59.1% (35%, 80.2%)	-0.9 (-1.5, -0.2)	61.3 (53.1, 69.4)	95.2% (93.3%, 97.1%)	0% (0%, 0%)
			High	59.1% (40.7%, 71.9%)	-0.9 (-1.3, -0.4)	29.1 (25.4, 32.8)	96.4% (94.8%, 98%)	0% (0%, 0%)
			Very High	57.4% (41.5%, 68.7%)	-2.6 (-3, -2.2)	28.9 (24.9, 33)	91.2% (88.7%, 93.7%)	0% (0%, 0%)

	Inferior	No	Very Low	57.7% (-30.2%, 93.9%)	-2.3 (-3.8, -0.8)	307.7 (244.6, 370.7)	95.6% (93.8%, 97.4%)	30.6% (26.6%, 34.6%)
			Low	57.4% (25.4%, 80.8%)	-2.6 (-3.4, -1.7)	105.2 (89.5, 120.9)	91% (88.5%, 93.5%)	2.2% (0.9%, 3.5%)
			Medium	56.7% (12.4%, 78.1%)	-3.3 (-3.9, -2.6)	72.2 (59.7, 84.7)	94.6% (92.6%, 96.6%)	0.2% (-0.2%, 0.6%)
			High	54% (37.1%, 67.4%)	-6 (-6.5, -5.5)	64.3 (57.4, 71.1)	81.2% (77.8%, 84.6%)	0% (0%, 0%)
			Very High	50.1% (34.7%, 61.6%)	-9.9 (-10.3, -9.4)	119.7 (110.4, 129)	37.8% (33.5%, 42.1%)	0% (0%, 0%)
		Yes	Very Low	58.4% (-27.1%, 93.3%)	-1.6 (-3, -0.1)	284.4 (229.5, 339.3)	96% (94.3%, 97.7%)	30.6% (26.6%, 34.6%)
			Low	59.4% (25.1%, 80.9%)	-0.6 (-1.4, 0.2)	87.3 (75, 99.5)	93% (90.8%, 95.2%)	0.8% (0%, 1.6%)
			Medium	59.2% (14.5%, 78.5%)	-0.8 (-1.5, -0.1)	59.9 (48.9, 70.9)	95% (93.1%, 96.9%)	0.2% (-0.2%, 0.6%)
			High	58.9% (43.3%, 72.4%)	-1.1 (-1.5, -0.7)	25.1 (21.9, 28.2)	96.6% (95%, 98.2%)	0% (0%, 0%)
			Very High	57.3% (42%, 67.4%)	-2.7 (-3.1, -2.3)	24.9 (21.5, 28.3)	90.2% (87.6%, 92.8%)	0% (0%, 0%)
20%	Superior	Yes	Very Low	57.3% (-22.6%, 93.5%)	-2.7 (-4.2, -1.2)	295.7 (249.8, 341.5)	97.2% (95.8%, 98.6%)	35.6% (31.4%, 39.8%)
			Low	56.8% (27.6%, 78.6%)	-3.2 (-3.9, -2.4)	91.7 (80, 103.4)	95% (93.1%, 96.9%)	0.6% (-0.1%, 1.3%)
			Medium	57.1% (34.4%, 78.4%)	-2.9 (-3.6, -2.1)	76.1 (66.5, 85.8)	94% (91.9%, 96.1%)	0% (0%, 0%)
			High	53.4% (31.4%, 69.5%)	-6.6 (-7.1, -6.1)	74 (65.5, 82.5)	82.4% (79.1%, 85.7%)	0% (0%, 0%)
			Very High	49.5% (27.5%, 63.7%)	-10.5 (-10.9, -10)	140.3 (128.8, 151.7)	45.2% (40.8%, 49.6%)	0% (0%, 0%)
	Similar	No	Very Low	56.4% (-22.5%, 91.7%)	-3.6 (-5.2, -2)	346.8 (281.9, 411.7)	94.6% (92.6%, 96.6%)	37% (32.8%, 41.2%)
			Low	55.5% (18.1%, 79.5%)	-4.5 (-5.3, -3.7)	106.5 (91.4, 121.7)	91.8% (89.4%, 94.2%)	1.4% (0.4%, 2.4%)
			Medium	54.7% (18.4%, 82.6%)	-5.3 (-6.1, -4.6)	99.5 (86.3, 112.6)	91.2% (88.7%, 93.7%)	0.2% (-0.2%, 0.6%)
			High	48% (25.1%, 65.6%)	-12 (-12.5, -11.4)	185.7 (169.8, 201.6)	45.4% (41%, 49.8%)	0% (0%, 0%)

			Very High	42% (24.2%, 60.2%)	-18 (-18.5, -17.4)	358.3 (338.5, 378.2)	6.2% (4.1%, 8.3%)	0% (0%, 0%)
		Yes	Very Low	58.2% (-32.8%, 90.4%)	-1.8 (-3.4, -0.3)	312.6 (254.5, 370.6)	96.4% (94.8%, 98%)	33.2% (29.1%, 37.3%)
			Low	58.8% (25.8%, 84.4%)	-1.2 (-2, -0.5)	83.5 (71.7, 95.3)	95.6% (93.8%, 97.4%)	1% (0.1%, 1.9%)
			Medium	59.4% (28.4%, 80.6%)	-0.6 (-1.3, 0.2)	67.7 (57.9, 77.5)	95% (93.1%, 96.9%)	0.4% (-0.2%, 1%)
			High	58% (40.2%, 72.3%)	-2 (-2.5, -1.6)	30.6 (26.4, 34.8)	94.8% (92.9%, 96.7%)	0% (0%, 0%)
			Very High	55.7% (38.9%, 70.2%)	-4.3 (-4.7, -3.9)	44.2 (38.6, 49.7)	85.6% (82.5%, 88.7%)	0% (0%, 0%)
	Inferior	No	Very Low	57.2% (-15.8%, 92.3%)	-2.8 (-4.3, -1.3)	299.2 (244.8, 353.7)	95% (93.1%, 96.9%)	30% (26%, 34%)
			Low	57.1% (25.1%, 80.7%)	-2.9 (-3.7, -2.1)	91 (78.2, 103.9)	93.4% (91.2%, 95.6%)	1% (0.1%, 1.9%)
			Medium	57% (27.4%, 79.1%)	-3 (-3.7, -2.2)	76.3 (65.3, 87.2)	92.6% (90.3%, 94.9%)	0.2% (-0.2%, 0.6%)
			High	54.1% (35.1%, 69.3%)	-5.9 (-6.4, -5.5)	63.8 (56.5, 71.1)	81.8% (78.4%, 85.2%)	0% (0%, 0%)
			Very High	50.7% (34.9%, 66%)	-9.3 (-9.7, -8.9)	107.3 (99, 115.6)	42.4% (38.1%, 46.7%)	0% (0%, 0%)
		Yes	Very Low	57.7% (-14.1%, 92.6%)	-2.3 (-3.8, -0.8)	293.9 (242.4, 345.4)	95.2% (93.3%, 97.1%)	30.2% (26.2%, 34.2%)
			Low	58.8% (26.8%, 81.8%)	-1.2 (-2, -0.4)	80.4 (69.7, 91.1)	95.4% (93.6%, 97.2%)	0.8% (0%, 1.6%)
			Medium	59% (32.1%, 79.5%)	-1 (-1.7, -0.3)	63.8 (55, 72.6)	94% (91.9%, 96.1%)	0% (0%, 0%)
			High	58.2% (39.2%, 73.1%)	-1.8 (-2.2, -1.3)	28.9 (24.9, 32.9)	94.2% (92.2%, 96.2%)	0% (0%, 0%)
			Very High	56.7% (40%, 69.9%)	-3.3 (-3.6, -2.9)	30 (25.9, 34)	87.8% (84.9%, 90.7%)	0% (0%, 0%)

Table G. Mean estimate, bias, mean squared error (MSE), coverage, and false negative rate for vaccine efficacy (VE) estimates against severe *Shigella* diarrhea from highly powered trials for all 50 simulation scenarios when using active surveillance for infection and a crude recurrent outcome regression model. Hybrid immunity was either not possible (no) or possible such that the first post-vaccination infection conferred additional immunity (yes). Bias was calculated as the average VE estimate for a set of simulations minus the true VE (60%). MSE was calculated as the average of the square of the estimate minus the true parameter. Coverage represents the percent of 95% confidence intervals (CIs) that contained the true VE (60%), and the false negative rate indicates the percent of 95% CIs that incorrectly indicated a non-significant effect.

VE against infection	Strength of infection-acquired immunity	Hybrid immunity	Force of infection	Mean VE estimate (range)	Bias (95% CI)	MSE (95% CI)	Coverage (95% CI)	False negatives (95% CI)
0%	Superior	Yes	Very Low	54.5% (-27.3%, 88%)	-5.5 (-7.1, -3.9)	365.4 (304.5, 426.2)	93.8% (91.7%, 95.9%)	42% (37.7%, 46.3%)
			Low	54% (20.3%, 81.1%)	-6 (-6.9, -5)	145.5 (124.8, 166.3)	87.8% (84.9%, 90.7%)	3.2% (1.7%, 4.7%)
			Medium	52.4% (18.4%, 71.6%)	-7.6 (-8.3, -6.8)	139 (119.3, 158.7)	86.6% (83.6%, 89.6%)	1.8% (0.6%, 3%)
			High	48.5% (27.2%, 66.2%)	-11.5 (-12.1, -10.9)	176.8 (161.4, 192.1)	51.8% (47.4%, 56.2%)	0% (0%, 0%)
			Very High	44.4% (26.2%, 58.5%)	-15.6 (-16.1, -15.2)	274.9 (258.8, 290.9)	12.4% (9.5%, 15.3%)	0% (0%, 0%)
	Similar	No	Very Low	57.2% (-26.7%, 93.7%)	-2.8 (-4.3, -1.3)	301.9 (247.2, 356.5)	96% (94.3%, 97.7%)	35.2% (31%, 39.4%)
			Low	54.5% (19.3%, 75.9%)	-5.5 (-6.4, -4.6)	134.3 (115, 153.6)	90.2% (87.6%, 92.8%)	2.8% (1.4%, 4.2%)
			Medium	53.7% (25.8%, 75.9%)	-6.3 (-7, -5.5)	110.3 (95.2, 125.5)	89.4% (86.7%, 92.1%)	1% (0.1%, 1.9%)
			High	47.3% (30.1%, 63.1%)	-12.7 (-13.2, -12.1)	195.6 (181.6, 209.7)	38% (33.7%, 42.3%)	0% (0%, 0%)
			Very High	40.4% (18.3%, 57.4%)	-19.6 (-20.1, -19.1)	416.9 (396.2, 437.6)	2.6% (1.2%, 4%)	0% (0%, 0%)
		Yes	Very Low	58.9% (-26.7%, 93.3%)	-1.1 (-2.6, 0.3)	279.6 (230.9, 328.4)	96% (94.3%, 97.7%)	31.6% (27.5%, 35.7%)
			Low	59% (25.9%, 79.8%)	-1 (-1.7, -0.2)	82.7 (71.7, 93.8)	96.2% (94.5%, 97.9%)	0.8% (0%, 1.6%)
			Medium	58.4% (33.8%, 79.6%)	-1.6 (-2.3, -0.9)	64.7 (56, 73.4)	95% (93.1%, 96.9%)	0% (0%, 0%)
			High	57.7% (39.3%, 70.6%)	-2.3 (-2.8, -1.8)	34.9 (30.4, 39.5)	93.8% (91.7%, 95.9%)	0% (0%, 0%)
			Very High	55.4% (39%, 66.5%)	-4.6 (-5, -4.2)	44.4 (38.8, 50.1)	84.8% (81.7%, 87.9%)	0% (0%, 0%)

	Inferior	No	Very Low	57.7% (-27.3%, 93.9%)	-2.3 (-3.8, -0.8)	306.2 (244.2, 368.3)	95.8% (94%, 97.6%)	30.6% (26.6%, 34.6%)
			Low	57.2% (25.4%, 80.4%)	-2.8 (-3.6, -1.9)	105.6 (89.9, 121.4)	91.4% (88.9%, 93.9%)	2.2% (0.9%, 3.5%)
			Medium	56.4% (12.9%, 77.5%)	-3.6 (-4.2, -2.9)	74.1 (61.5, 86.8)	93.8% (91.7%, 95.9%)	0.2% (-0.2%, 0.6%)
			High	53.3% (36.2%, 66.8%)	-6.7 (-7.1, -6.2)	72.8 (65.3, 80.3)	77.8% (74.2%, 81.4%)	0% (0%, 0%)
			Very High	49.2% (33%, 60.7%)	-10.8 (-11.2, -10.4)	140.1 (130, 150.3)	28.6% (24.6%, 32.6%)	0% (0%, 0%)
		Yes	Very Low	58.4% (-26.7%, 93.3%)	-1.6 (-3.1, -0.1)	284.7 (229.8, 339.7)	96% (94.3%, 97.7%)	30.6% (26.6%, 34.6%)
			Low	59.2% (24.1%, 80.7%)	-0.8 (-1.7, 0)	87.8 (75.4, 100.3)	93.2% (91%, 95.4%)	0.8% (0%, 1.6%)
			Medium	58.8% (15%, 78.1%)	-1.2 (-1.9, -0.5)	61 (49.9, 72.1)	95.4% (93.6%, 97.2%)	0.2% (-0.2%, 0.6%)
			High	58.1% (41.9%, 72.3%)	-1.9 (-2.3, -1.5)	28.3 (24.7, 31.9)	94.4% (92.4%, 96.4%)	0% (0%, 0%)
			Very High	56.2% (41%, 66.4%)	-3.8 (-4.2, -3.5)	32.9 (28.8, 37.1)	85.2% (82.1%, 88.3%)	0% (0%, 0%)
20%	Superior	Yes	Very Low	57.1% (-23.1%, 93.5%)	-2.9 (-4.4, -1.4)	298.1 (251.7, 344.5)	97.4% (96%, 98.8%)	35% (30.8%, 39.2%)
			Low	56.2% (26.5%, 78.3%)	-3.8 (-4.6, -3)	97.6 (85.2, 110.1)	94.8% (92.9%, 96.7%)	1% (0.1%, 1.9%)
			Medium	56.4% (33.3%, 77.8%)	-3.6 (-4.4, -2.9)	82.5 (72, 93)	92.8% (90.5%, 95.1%)	0% (0%, 0%)
			High	51.9% (29.5%, 68.2%)	-8.1 (-8.6, -7.6)	97.3 (87.1, 107.4)	74.6% (70.8%, 78.4%)	0% (0%, 0%)
			Very High	47.6% (24.7%, 61.7%)	-12.4 (-12.9, -11.9)	186.3 (172.6, 200.1)	32.4% (28.3%, 36.5%)	0% (0%, 0%)
	Similar	No	Very Low	56.3% (-23.1%, 91.7%)	-3.7 (-5.3, -2.1)	349 (283.2, 414.9)	94.6% (92.6%, 96.6%)	36.6% (32.4%, 40.8%)
			Low	55.1% (18%, 79.6%)	-4.9 (-5.7, -4.1)	110.5 (95, 126)	91.8% (89.4%, 94.2%)	1.8% (0.6%, 3%)
			Medium	54.2% (17.4%, 82.2%)	-5.8 (-6.6, -5.1)	106.3 (92.3, 120.2)	90.4% (87.8%, 93%)	0.4% (-0.2%, 1%)
			High	47.1% (23.9%, 65.3%)	-12.9 (-13.5, -12.3)	209.6 (192.6, 226.6)	38.4% (34.1%, 42.7%)	0% (0%, 0%)

			Very High	40.9% (22.9%, 59.3%)	-19.1 (-19.6, -18.5)	400.2 (379.1, 421.4)	3.8% (2.1%, 5.5%)	0% (0%, 0%)
		Yes	Very Low	58% (-33.3%, 90.3%)	-2 (-3.5, -0.4)	315.2 (256.2, 374.1)	96.4% (94.8%, 98%)	32.6% (28.5%, 36.7%)
			Low	58.2% (25.4%, 84.2%)	-1.8 (-2.6, -1)	86.8 (74.4, 99.2)	95.2% (93.3%, 97.1%)	1.4% (0.4%, 2.4%)
			Medium	58.8% (27.6%, 80.2%)	-1.2 (-2, -0.5)	70.3 (60, 80.6)	95.6% (93.8%, 97.4%)	0.4% (-0.2%, 1%)
			High	56.6% (37.9%, 71.5%)	-3.4 (-3.9, -3)	39.6 (34.4, 44.9)	93.2% (91%, 95.4%)	0% (0%, 0%)
			Very High	53.8% (36.6%, 68.7%)	-6.2 (-6.6, -5.7)	65.5 (58.2, 72.8)	73.2% (69.3%, 77.1%)	0% (0%, 0%)
	Inferior	No	Very Low	57.1% (-16.6%, 92.3%)	-2.9 (-4.4, -1.4)	299.3 (244.6, 354.1)	95.2% (93.3%, 97.1%)	30% (26%, 34%)
			Low	56.9% (26.2%, 80.6%)	-3.1 (-3.9, -2.3)	91.6 (79, 104.2)	93.8% (91.7%, 95.9%)	1% (0.1%, 1.9%)
			Medium	56.8% (27.5%, 78.8%)	-3.2 (-3.9, -2.5)	78.2 (67, 89.4)	92.6% (90.3%, 94.9%)	0.4% (-0.2%, 1%)
			High	53.5% (34.5%, 68.7%)	-6.5 (-7, -6.1)	71.6 (63.8, 79.4)	77.6% (73.9%, 81.3%)	0% (0%, 0%)
			Very High	49.8% (34.7%, 64.9%)	-10.2 (-10.6, -9.8)	124.5 (115.5, 133.5)	34.6% (30.4%, 38.8%)	0% (0%, 0%)
		Yes	Very Low	57.6% (-14.3%, 92.6%)	-2.4 (-3.8, -0.9)	294.9 (242.9, 346.8)	95.6% (93.8%, 97.4%)	30.2% (26.2%, 34.2%)
			Low	58.5% (29.3%, 81.8%)	-1.5 (-2.3, -0.7)	80.8 (70.4, 91.2)	95.2% (93.3%, 97.1%)	0.6% (-0.1%, 1.3%)
			Medium	58.7% (30.6%, 79.2%)	-1.3 (-2, -0.6)	65.4 (56.2, 74.5)	93.6% (91.5%, 95.7%)	0% (0%, 0%)
			High	57.5% (38.3%, 72.3%)	-2.5 (-2.9, -2)	32.8 (28.3, 37.2)	93% (90.8%, 95.2%)	0% (0%, 0%)
			Very High	55.6% (38.2%, 69%)	-4.4 (-4.8, -4)	38.9 (34.2, 43.6)	82.4% (79.1%, 85.7%)	0% (0%, 0%)

Table H. Mean estimate, bias, mean squared error (MSE), coverage, and false negative rate for vaccine efficacy (VE) estimates against severe *Shigella* diarrhea from highly powered trials for all 50 simulation scenarios when using symptom-based reporting and a crude recurrent outcome regression model. Hybrid immunity was either not possible (no) or possible such that the first post-vaccination infection conferred additional immunity (yes). Bias was calculated as the average VE estimate for a set of simulations minus the true VE (60%). MSE was calculated as the average of the square of the estimate minus the true parameter. Coverage represents the percent of 95% confidence intervals (CIs) that contained the true VE (60%), and the false negative rate indicates the percent of 95% CIs that incorrectly indicated a non-significant effect.

VE against infection	Strength of infection-acquired immunity	Hybrid immunity	Force of infection	Mean VE estimate (range)	Bias (95% CI)	MSE (95% CI)	Coverage (95% CI)	False negatives (95% CI)
0%	Superior	Yes	Very Low	54.5% (-27.3%, 88%)	-5.5 (-7.1, -3.9)	365.3 (304.5, 426.2)	93.8% (91.7%, 95.9%)	42% (37.7%, 46.3%)
			Low	54% (20.3%, 81.1%)	-6 (-6.9, -5)	145.4 (124.7, 166.2)	87.8% (84.9%, 90.7%)	3.2% (1.7%, 4.7%)
			Medium	52.5% (18.4%, 71.6%)	-7.5 (-8.3, -6.8)	138.9 (119.2, 158.6)	86.6% (83.6%, 89.6%)	1.8% (0.6%, 3%)
			High	48.5% (27.2%, 66.2%)	-11.5 (-12.1, -10.9)	176.4 (161.1, 191.7)	51.8% (47.4%, 56.2%)	0% (0%, 0%)
			Very High	44.4% (26.3%, 58.5%)	-15.6 (-16.1, -15.1)	274.2 (258.1, 290.2)	12.4% (9.5%, 15.3%)	0% (0%, 0%)
	Similar	No	Very Low	57.2% (-26.7%, 93.8%)	-2.8 (-4.3, -1.3)	301.8 (247.2, 356.5)	96% (94.3%, 97.7%)	35.2% (31%, 39.4%)
			Low	54.5% (19.3%, 75.9%)	-5.5 (-6.4, -4.6)	134.2 (114.9, 153.5)	90.2% (87.6%, 92.8%)	2.8% (1.4%, 4.2%)
			Medium	53.7% (25.8%, 75.9%)	-6.3 (-7, -5.5)	110.2 (95, 125.3)	89.4% (86.7%, 92.1%)	1% (0.1%, 1.9%)
			High	47.3% (30.1%, 63.1%)	-12.7 (-13.2, -12.1)	195.2 (181.2, 209.2)	38.2% (33.9%, 42.5%)	0% (0%, 0%)
			Very High	40.4% (18.3%, 57.5%)	-19.6 (-20.1, -19.1)	416 (395.3, 436.6)	2.6% (1.2%, 4%)	0% (0%, 0%)
		Yes	Very Low	58.9% (-26.7%, 93.3%)	-1.1 (-2.6, 0.3)	279.6 (230.9, 328.3)	96% (94.3%, 97.7%)	31.6% (27.5%, 35.7%)
			Low	59.1% (25.9%, 79.8%)	-0.9 (-1.7, -0.2)	82.7 (71.7, 93.7)	96.2% (94.5%, 97.9%)	0.8% (0%, 1.6%)
			Medium	58.4% (33.8%, 79.6%)	-1.6 (-2.3, -0.9)	64.6 (55.9, 73.4)	95% (93.1%, 96.9%)	0% (0%, 0%)
			High	57.7% (39.3%, 70.7%)	-2.3 (-2.8, -1.8)	34.8 (30.3, 39.4)	94% (91.9%, 96.1%)	0% (0%, 0%)
			Very High	55.5% (39.1%, 66.6%)	-4.5 (-5, -4.1)	44.2 (38.6, 49.8)	84.8% (81.7%, 87.9%)	0% (0%, 0%)

	Inferior	No	Very Low	57.7% (-27.3%, 93.9%)	-2.3 (-3.8, -0.8)	306.2 (244.1, 368.2)	95.8% (94%, 97.6%)	30.6% (26.6%, 34.6%)
			Low	57.2% (25.4%, 80.4%)	-2.8 (-3.6, -1.9)	105.6 (89.9, 121.3)	91.4% (88.9%, 93.9%)	2.2% (0.9%, 3.5%)
			Medium	56.5% (12.9%, 77.5%)	-3.5 (-4.2, -2.9)	74 (61.4, 86.7)	93.8% (91.7%, 95.9%)	0.2% (-0.2%, 0.6%)
			High	53.4% (36.2%, 66.8%)	-6.6 (-7.1, -6.2)	72.5 (65, 79.9)	77.8% (74.2%, 81.4%)	0% (0%, 0%)
			Very High	49.2% (33%, 60.8%)	-10.8 (-11.2, -10.4)	139.3 (129.2, 149.4)	28.8% (24.8%, 32.8%)	0% (0%, 0%)
		Yes	Very Low	58.4% (-26.7%, 93.3%)	-1.6 (-3.1, -0.1)	284.7 (229.8, 339.6)	96% (94.3%, 97.7%)	30.6% (26.6%, 34.6%)
			Low	59.2% (24.1%, 80.7%)	-0.8 (-1.7, 0)	87.8 (75.3, 100.2)	93.2% (91%, 95.4%)	0.8% (0%, 1.6%)
			Medium	58.8% (15%, 78.1%)	-1.2 (-1.8, -0.5)	61 (49.9, 72)	95.4% (93.6%, 97.2%)	0.2% (-0.2%, 0.6%)
			High	58.1% (41.9%, 72.3%)	-1.9 (-2.3, -1.4)	28.2 (24.6, 31.8)	94.4% (92.4%, 96.4%)	0% (0%, 0%)
			Very High	56.2% (41%, 66.4%)	-3.8 (-4.2, -3.4)	32.6 (28.5, 36.8)	85.6% (82.5%, 88.7%)	0% (0%, 0%)
20%	Superior	Yes	Very Low	57.1% (-23.1%, 93.5%)	-2.9 (-4.4, -1.4)	298.1 (251.7, 344.5)	97.4% (96%, 98.8%)	35% (30.8%, 39.2%)
			Low	56.2% (26.5%, 78.3%)	-3.8 (-4.6, -3)	97.7 (85.2, 110.2)	94.8% (92.9%, 96.7%)	1% (0.1%, 1.9%)
			Medium	56.4% (33.3%, 77.8%)	-3.6 (-4.4, -2.9)	82.5 (72, 93)	92.8% (90.5%, 95.1%)	0% (0%, 0%)
			High	51.9% (29.5%, 68.2%)	-8.1 (-8.6, -7.6)	97.4 (87.3, 107.6)	74.6% (70.8%, 78.4%)	0% (0%, 0%)
			Very High	47.6% (24.7%, 61.7%)	-12.4 (-12.9, -11.9)	186.8 (173.1, 200.5)	32.2% (28.1%, 36.3%)	0% (0%, 0%)
	Similar	No	Very Low	56.3% (-23.1%, 91.7%)	-3.7 (-5.3, -2.1)	349.1 (283.2, 414.9)	94.6% (92.6%, 96.6%)	36.6% (32.4%, 40.8%)
			Low	55.1% (18%, 79.5%)	-4.9 (-5.7, -4.1)	110.6 (95.1, 126.1)	91.8% (89.4%, 94.2%)	1.8% (0.6%, 3%)
			Medium	54.2% (17.4%, 82.2%)	-5.8 (-6.6, -5.1)	106.3 (92.4, 120.2)	90.4% (87.8%, 93%)	0.4% (-0.2%, 1%)
			High	47.1% (23.9%, 65.3%)	-12.9 (-13.5, -12.3)	209.7 (192.7, 226.8)	38.2% (33.9%, 42.5%)	0% (0%, 0%)

			Very High	40.9% (22.9%, 59.3%)	-19.1 (-19.6, -18.5)	400.6 (379.4, 421.8)	3.8% (2.1%, 5.5%)	0% (0%, 0%)
		Yes	Very Low	58% (-33.3%, 90.3%)	-2 (-3.5, -0.4)	315.2 (256.2, 374.2)	96.4% (94.8%, 98%)	32.6% (28.5%, 36.7%)
			Low	58.2% (25.4%, 84.2%)	-1.8 (-2.6, -1)	86.8 (74.4, 99.3)	95.2% (93.3%, 97.1%)	1.4% (0.4%, 2.4%)
			Medium	58.8% (27.5%, 80.2%)	-1.2 (-2, -0.5)	70.3 (60, 80.6)	95.6% (93.8%, 97.4%)	0.4% (-0.2%, 1%)
			High	56.6% (37.9%, 71.5%)	-3.4 (-3.9, -3)	39.7 (34.5, 44.9)	93.2% (91%, 95.4%)	0% (0%, 0%)
			Very High	53.8% (36.6%, 68.7%)	-6.2 (-6.6, -5.7)	65.7 (58.3, 73)	73.2% (69.3%, 77.1%)	0% (0%, 0%)
	Inferior	No	Very Low	57.1% (-16.7%, 92.3%)	-2.9 (-4.4, -1.4)	299.4 (244.6, 354.1)	95.2% (93.3%, 97.1%)	30% (26%, 34%)
			Low	56.9% (26.2%, 80.6%)	-3.1 (-3.9, -2.4)	91.6 (79, 104.2)	93.8% (91.7%, 95.9%)	1% (0.1%, 1.9%)
			Medium	56.8% (27.5%, 78.8%)	-3.2 (-4, -2.5)	78.3 (67.1, 89.5)	92.6% (90.3%, 94.9%)	0.4% (-0.2%, 1%)
			High	53.5% (34.5%, 68.7%)	-6.5 (-7, -6.1)	71.7 (63.9, 79.6)	77.6% (73.9%, 81.3%)	0% (0%, 0%)
			Very High	49.8% (34.6%, 64.8%)	-10.2 (-10.6, -9.8)	125 (116, 134.1)	34.2% (30%, 38.4%)	0% (0%, 0%)
		Yes	Very Low	57.6% (-14.3%, 92.6%)	-2.4 (-3.8, -0.9)	294.9 (242.9, 346.9)	95.6% (93.8%, 97.4%)	30.2% (26.2%, 34.2%)
			Low	58.5% (29.3%, 81.8%)	-1.5 (-2.3, -0.7)	80.8 (70.4, 91.2)	95.2% (93.3%, 97.1%)	0.6% (-0.1%, 1.3%)
			Medium	58.7% (30.6%, 79.2%)	-1.3 (-2, -0.6)	65.4 (56.2, 74.5)	93.6% (91.5%, 95.7%)	0% (0%, 0%)
			High	57.5% (38.3%, 72.3%)	-2.5 (-2.9, -2)	32.8 (28.4, 37.3)	92.8% (90.5%, 95.1%)	0% (0%, 0%)
			Very High	55.6% (38.2%, 68.9%)	-4.4 (-4.8, -4)	39.1 (34.4, 43.9)	82.2% (78.8%, 85.6%)	0% (0%, 0%)

Table I. Mean estimate, bias, mean squared error (MSE), coverage, and false negative rate for vaccine efficacy (VE) estimates against severe *Shigella* diarrhea from highly powered trials for all 6 design approaches applied to stratified datasets for the first and second year of follow-up. These results are for scenarios where VE against infection was 0%, infection- and vaccine-acquired immunity were comparable, and hybrid immunity was not possible. Bias was calculated as the average VE estimate for a set of simulations minus the true VE (60%). MSE was calculated as the average of the square of the estimate minus the true parameter. Coverage represents the percent of 95% confidence intervals (CIs) that contained the true VE (60%), and the false negative rate indicates the percent of 95% CIs that incorrectly indicated a non-significant effect.

Regression model type	Data collection approach	Force of infection	Year of life	Mean VE estimate (range)	Bias (95% CI)	MSE (95% CI)	Coverage (95% CI)	False negatives (95% CI)
Single outcome	Active surveillance	Very Low	First	57.6% (-88%, 100%)	-2.4 (-4.3, -0.5)	476.1 (367.7, 584.5)	97% (95.5%, 98.5%)	47.8% (43.4%, 52.2%)
			Second	50.5% (-202.2%, 100%)	-9.5 (-13.5, -5.5)	2164.1 (1528.3, 2799.9)	98% (96.8%, 99.2%)	92.4% (90.1%, 94.7%)
		Low	First	58.6% (9.4%, 83.9%)	-1.4 (-2.3, -0.4)	124 (103.8, 144.2)	95.4% (93.6%, 97.2%)	4.6% (2.8%, 6.4%)
			Second	57.3% (-94.6%, 100%)	-2.7 (-4.6, -0.8)	480.5 (351.1, 609.8)	96.4% (94.8%, 98%)	48.4% (44%, 52.8%)
		Medium	First	58.7% (1.6%, 81.7%)	-1.3 (-2.2, -0.5)	96.9 (77.4, 116.4)	95.4% (93.6%, 97.2%)	2.4% (1.1%, 3.7%)
			Second	58% (-47%, 94.9%)	-2 (-3.6, -0.3)	359 (283.5, 434.5)	97.4% (96%, 98.8%)	40.6% (36.3%, 44.9%)
		High	First	59.1% (36.5%, 74.9%)	-0.9 (-1.5, -0.3)	46.4 (40.9, 52)	95.4% (93.6%, 97.2%)	0% (0%, 0%)
			Second	59.7% (-4.6%, 89.2%)	-0.3 (-1.7, 1.1)	247.6 (216.1, 279)	98.4% (97.3%, 99.5%)	29% (25%, 33%)
		Very High	First	59.7% (42.7%, 74.5%)	-0.3 (-0.8, 0.1)	29.4 (25.7, 33.1)	95.8% (94%, 97.6%)	0% (0%, 0%)
			Second	56.8% (-19.4%, 100%)	-3.2 (-4.8, -1.5)	357.6 (298.3, 417)	96.6% (95%, 98.2%)	43% (38.7%, 47.3%)
	Symptom-based reporting	Very Low	First	57.1% (-88.3%, 100%)	-2.9 (-4.8, -0.9)	479.8 (367.2, 592.5)	97% (95.5%, 98.5%)	48% (43.6%, 52.4%)
			Second	48.5% (-196.6%, 100%)	-11.5 (-15.5, -7.4)	2238.2 (1603.8, 2872.6)	96.8% (95.3%, 98.3%)	91% (88.5%, 93.5%)
		Low	First	57% (17.9%, 82.6%)	-3 (-4, -2)	131.3 (111.4, 151.3)	94.4% (92.4%, 96.4%)	4.8% (2.9%, 6.7%)

			Second	51.1% (-49.3%, 95.2%)	-8.9 (-10.9, -7)	557.4 (442.7, 672.1)	92.6% (90.3%, 94.9%)	53.2% (48.8%, 57.6%)
		Medium	First	56.5% (-1%, 78.8%)	-3.5 (-4.4, -2.6)	109.8 (87.4, 132.1)	94.4% (92.4%, 96.4%)	2.2% (0.9%, 3.5%)
			Second	50.6% (-91.4%, 87.5%)	-9.4 (-11.1, -7.6)	470 (357.5, 582.6)	92.2% (89.8%, 94.6%)	46.2% (41.8%, 50.6%)
		High	First	54.2% (32.6%, 69.3%)	-5.8 (-6.4, -5.2)	78.4 (68.9, 87.8)	84.6% (81.4%, 87.8%)	0% (0%, 0%)
			Second	40.7% (-19.4%, 78.9%)	-19.3 (-20.7, -17.9)	632.8 (555.5, 710)	72.4% (68.5%, 76.3%)	47.8% (43.4%, 52.2%)
		Very High	First	51.4% (32.6%, 69.5%)	-8.6 (-9.1, -8.2)	105.8 (96.2, 115.5)	63.8% (59.6%, 68%)	0% (0%, 0%)
			Second	26.3% (-47.1%, 69.8%)	-33.7 (-35.3, -32.1)	1464 (1328.5, 1599.5)	29.4% (25.4%, 33.4%)	71.4% (67.4%, 75.4%)
Stratified recurrent outcome	Active surveillance	Very Low	First	56.8% (-88%, 100%)	-3.2 (-5.1, -1.3)	484.2 (370.2, 598.3)	96.6% (95%, 98.2%)	47.4% (43%, 51.8%)
			Second	47.2% (-202.2%, 100%)	-12.8 (-16.9, -8.7)	2365.8 (1677.1, 3054.6)	90.2% (87.6%, 92.8%)	84.6% (81.4%, 87.8%)
		Low	First	56% (16.1%, 82.5%)	-4 (-5, -3)	142.9 (120.5, 165.3)	94.4% (92.4%, 96.4%)	6.4% (4.3%, 8.5%)
			Second	48.3% (-66.1%, 95%)	-11.7 (-13.6, -9.8)	609.8 (487, 732.5)	90.8% (88.3%, 93.3%)	56% (51.6%, 60.4%)
		Medium	First	55.2% (-0.5%, 76.8%)	-4.8 (-5.6, -3.9)	120.6 (97.6, 143.5)	93% (90.8%, 95.2%)	3% (1.5%, 4.5%)
			Second	47.2% (-101.1%, 85%)	-12.8 (-14.5, -11.1)	543.4 (419, 667.7)	89.6% (86.9%, 92.3%)	51.2% (46.8%, 55.6%)
		High	First	51.3% (28.6%, 66.2%)	-8.7 (-9.3, -8.1)	120.6 (108.1, 133.1)	74% (70.2%, 77.8%)	0% (0%, 0%)
			Second	32.7% (-44.9%, 67.4%)	-27.3 (-28.7, -25.9)	988.4 (893.3, 1083.6)	46% (41.6%, 50.4%)	58% (53.7%, 62.3%)
		Very High	First	46.5% (25.2%, 61.4%)	-13.5 (-14, -13)	216.8 (201.7, 231.9)	28.2% (24.3%, 32.1%)	0% (0%, 0%)
			Second	22.8% (-34.3%, 57.3%)	-37.2 (-38.5, -36)	1,602.9 (1,491.5, 1,714.2)	6.6% (4.4%, 8.8%)	63.2% (59%, 67.4%)
	Symptom-based reporting	Very Low	First	16.8% (-66.1%, 60.1%)	-43.2 (-44.8, -41.7)	2169.6 (2015.2, 2324.1)	7% (4.8%, 9.2%)	79.8% (76.3%, 83.3%)

			Second	56.9% (-88.3%, 100%)	-3.1 (-5, -1.2)	481.8 (367.9, 595.7)	96.8% (95.3%, 98.3%)	48.2% (43.8%, 52.6%)
		Low	First	47.4% (-216.5%, 100%)	-12.6 (-16.7, -8.5)	2347.5 (1662.2, 3032.7)	90.2% (87.6%, 92.8%)	84.6% (81.4%, 87.8%)
			Second	56.3% (16.5%, 82.7%)	-3.7 (-4.7, -2.7)	139.9 (118, 161.7)	94.2% (92.2%, 96.2%)	5.6% (3.6%, 7.6%)
		Medium	First	49% (-63.3%, 95.2%)	-11 (-12.9, -9.1)	585.6 (467, 704.3)	91.6% (89.2%, 94%)	55.4% (51%, 59.8%)
			Second	55.6% (1.2%, 77.2%)	-4.4 (-5.3, -3.5)	115.6 (93.7, 137.6)	94% (91.9%, 96.1%)	2.8% (1.4%, 4.2%)
		High	First	52.1% (29.6%, 67%)	-7.9 (-8.5, -7.3)	106.9 (95.3, 118.6)	79.2% (75.6%, 82.8%)	0% (0%, 0%)
			Second	34.4% (-41.7%, 68.6%)	-25.6 (-27, -24.3)	893.7 (804.2, 983.2)	51.4% (47%, 55.8%)	53.6% (49.2%, 58%)
		Very High	First	47.6% (26.5%, 62.7%)	-12.4 (-12.9, -11.9)	188.2 (174.2, 202.1)	35% (30.8%, 39.2%)	0% (0%, 0%)
			Second	18.2% (-64%, 60%)	-41.8 (-43.4, -40.3)	2047 (1898.2, 2195.9)	7.6% (5.3%, 9.9%)	77.8% (74.2%, 81.4%)
Crude recurrent outcome	Active surveillance	Very Low	First	56.8% (-88.9%, 100%)	-3.2 (-5.1, -1.3)	484.5 (369.8, 599.2)	96.6% (95%, 98.2%)	47.4% (43%, 51.8%)
			Second	47.3% (-200%, 100%)	-12.7 (-16.9, -8.6)	2355.7 (1670.4, 3041)	90.4% (87.8%, 93%)	84% (80.8%, 87.2%)
		Low	First	56% (16.3%, 82.5%)	-4 (-5, -3)	142.7 (120.4, 165)	94.2% (92.2%, 96.2%)	6.2% (4.1%, 8.3%)
			Second	48.3% (-66.7%, 95%)	-11.7 (-13.6, -9.8)	609 (486, 731.9)	91% (88.5%, 93.5%)	56% (51.6%, 60.4%)
		Medium	First	55.2% (0%, 76.8%)	-4.8 (-5.6, -3.9)	120.1 (97.3, 142.9)	93% (90.8%, 95.2%)	3% (1.5%, 4.5%)
			Second	47.2% (-100%, 84.8%)	-12.8 (-14.5, -11.1)	541.6 (418.6, 664.7)	89.6% (86.9%, 92.3%)	50.8% (46.4%, 55.2%)
		High	First	51.3% (28.7%, 66%)	-8.7 (-9.3, -8.1)	120.2 (107.7, 132.6)	73.8% (69.9%, 77.7%)	0% (0%, 0%)
			Second	32.7% (-44.8%, 67.4%)	-27.3 (-28.6, -25.9)	985.9 (891.2, 1080.7)	45.6% (41.2%, 50%)	57.8% (53.5%, 62.1%)
		Very High	First	46.5% (25.7%, 61.6%)	-13.5 (-14, -13)	216.9 (201.9, 231.9)	27% (23.1%, 30.9%)	0% (0%, 0%)

			Second	16.8%<br>(-66.7%, 60.2%)	-43.2<br>(-44.8, -41.7)	2170<br>(2015.7, 2324.3)	7%<br>(4.8%, 9.2%)	80.2%<br>(76.7%, 83.7%)
	Symptom-based reporting	Very Low	First	56.8%<br>(-88.9%, 100%)	-3.2<br>(-5.1, -1.3)	484.5<br>(369.8, 599.2)	96.6%<br>(95%, 98.2%)	47.4%<br>(43%, 51.8%)
			Second	47.3%<br>(-200%, 100%)	-12.7<br>(-16.8, -8.6)	2355.5<br>(1670.2, 3040.8)	90.4%<br>(87.8%, 93%)	84%<br>(80.8%, 87.2%)
		Low	First	56%<br>(16.3%, 82.5%)	-4<br>(-5, -3)	142.6<br>(120.3, 164.9)	94.2%<br>(92.2%, 96.2%)	6.2%<br>(4.1%, 8.3%)
			Second	48.3%<br>(-66.7%, 95%)	-11.7<br>(-13.6, -9.8)	608.7<br>(485.8, 731.6)	91%<br>(88.5%, 93.5%)	56%<br>(51.6%, 60.4%)
		Medium	First	55.2%<br>(0%, 76.8%)	-4.8<br>(-5.6, -3.9)	120<br>(97.2, 142.7)	93%<br>(90.8%, 95.2%)	3%<br>(1.5%, 4.5%)
			Second	47.2%<br>(-100%, 84.9%)	-12.8<br>(-14.5, -11.1)	541.3<br>(418.4, 664.3)	89.6%<br>(86.9%, 92.3%)	50.8%<br>(46.4%, 55.2%)
		High	First	51.4%<br>(28.7%, 66.1%)	-8.6<br>(-9.2, -8.1)	119.8<br>(107.4, 132.3)	73.8%<br>(69.9%, 77.7%)	0%<br>(0%, 0%)
			Second	32.7%<br>(-44.8%, 67.5%)	-27.3<br>(-28.6, -25.9)	985.1<br>(890.4, 1079.8)	45.6%<br>(41.2%, 50%)	57.8%<br>(53.5%, 62.1%)
		Very High	First	46.5%<br>(25.8%, 61.6%)	-13.5<br>(-14, -13)	216.1<br>(201.1, 231.1)	27.6%<br>(23.7%, 31.5%)	0%<br>(0%, 0%)
			Second	16.8%<br>(-66.6%, 60.2%)	-43.2<br>(-44.7, -41.7)	2169<br>(2014.7, 2323.3)	7%<br>(4.8%, 9.2%)	80.2%<br>(76.7%, 83.7%)

Table J. Mean size of each trial arm, estimate, bias, mean squared error (MSE), coverage, and false negative rate for vaccine efficacy (VE) estimates against severe *Shigella* diarrhea from realistically sized trials for all 50 simulation scenarios when using active surveillance for infection and a single outcome regression model. Hybrid immunity was either not possible (no) or possible such that the first post-vaccination infection conferred additional immunity (yes). Bias was calculated as the average VE estimate for a set of simulations minus the true VE (60%). MSE was calculated as the average of the square of the estimate minus the true parameter. Coverage represents the percent of 95% confidence intervals (CIs) that contained the true VE (60%), and the false negative rate indicates the percent of 95% CIs that incorrectly indicated a non-significant effect.

VE against infection	Strength of infection-acquired immunity	Hybrid immunity	Force of infection	Mean VE estimate (range)	Bias (95% CI)	MSE (95% CI)	Coverage (95% CI)	False negatives (95% CI)
0%	Superior	Yes	Very Low	52.2% (-133.8%, 100%)	-7.8 (-8.9, -6.8)	731.9 (653.4, 810.4)	95.9% (95.1%, 96.7%)	62.2% (60.3%, 64.1%)
			Low	55.4% (-41.6%, 94.3%)	-4.6 (-5.2, -3.9)	286.5 (261.2, 311.7)	95.5% (94.7%, 96.3%)	29.1% (27.3%, 30.9%)
			Medium	54.2% (-44%, 91.9%)	-5.8 (-6.5, -5.1)	330.1 (302.9, 357.3)	94.4% (93.5%, 95.3%)	36.8% (34.9%, 38.7%)
			High	54.8% (-18.3%, 93.5%)	-5.2 (-5.9, -4.6)	290.6 (269.4, 311.7)	94.4% (93.5%, 95.3%)	31.8% (30%, 33.7%)
			Very High	55% (-36.7%, 95.6%)	-5 (-5.7, -4.4)	295.1 (272, 318.2)	94% (93%, 94.9%)	31.8% (30%, 33.6%)
	Similar	No	Very Low	57.3% (-101.5%, 100%)	-2.7 (-3.6, -1.8)	528.6 (479.8, 577.4)	97.4% (96.8%, 98.1%)	54.8% (52.8%, 56.7%)
			Low	58.1% (-48.5%, 93.1%)	-1.9 (-2.5, -1.3)	236.1 (217.7, 254.4)	96.4% (95.6%, 97.1%)	25% (23.3%, 26.7%)
			Medium	58.4% (-54.1%, 95.8%)	-1.6 (-2.3, -1)	258.9 (237.2, 280.6)	96% (95.3%, 96.8%)	27.9% (26.1%, 29.6%)
			High	58.3% (-29.9%, 100%)	-1.7 (-2.3, -1.1)	213.1 (196.8, 229.4)	96.2% (95.5%, 97%)	20.9% (19.3%, 22.5%)
			Very High	58.6% (2.3%, 89.4%)	-1.4 (-1.9, -0.9)	163.3 (152.3, 174.4)	95.2% (94.4%, 96.1%)	10.7% (9.5%, 11.9%)
		Yes	Very Low	57.4% (-59.8%, 100%)	-2.6 (-3.5, -1.7)	532.5 (486.9, 578)	97.8% (97.3%, 98.4%)	54.1% (52.2%, 56.1%)
			Low	58.6% (-9.1%, 94.4%)	-1.4 (-1.9, -0.8)	218.4 (202.9, 233.8)	96.9% (96.2%, 97.6%)	23.8% (22.1%, 25.4%)
			Medium	58.2% (-48.8%, 95.7%)	-1.8 (-2.4, -1.1)	267.4 (243.3, 291.5)	96.1% (95.3%, 96.8%)	27.5% (25.8%, 29.3%)
			High	58% (-40.4%, 95.2%)	-2 (-2.6, -1.4)	251.8 (231.9, 271.8)	95.4% (94.6%, 96.2%)	26.4% (24.6%, 28.1%)
			Very High	58.1% (-15.6%, 96.1%)	-1.9 (-2.5, -1.2)	258.5 (239.7, 277.4)	95.2% (94.3%, 96%)	26.3% (24.6%, 28%)

	Inferior	No	Very Low	57.1% (-120.5%, 100%)	-2.9 (-3.8, -2)	533.9 (483.1, 584.6)	97.6% (97%, 98.2%)	55.7% (53.8%, 57.7%)
			Low	58.2% (-21.1%, 90.2%)	-1.8 (-2.4, -1.2)	241.5 (223.5, 259.5)	95.8% (95.1%, 96.6%)	24.4% (22.7%, 26.1%)
			Medium	57.9% (-24%, 96.2%)	-2.1 (-2.7, -1.5)	261 (241.5, 280.5)	95.7% (94.9%, 96.5%)	29.4% (27.6%, 31.2%)
			High	57.7% (-21.1%, 97.2%)	-2.3 (-3, -1.7)	251.4 (233.4, 269.3)	95.6% (94.8%, 96.4%)	28% (26.2%, 29.8%)
			Very High	58.7% (-13.4%, 94.2%)	-1.3 (-1.9, -0.7)	225 (209.2, 240.8)	96.2% (95.5%, 97%)	24% (22.4%, 25.7%)
		Yes	Very Low	57.1% (-120.5%, 100%)	-2.9 (-3.8, -2)	538.7 (488.2, 589.3)	97.4% (96.8%, 98%)	55.7% (53.7%, 57.6%)
			Low	58.3% (-20.9%, 91.3%)	-1.7 (-2.3, -1.1)	244.7 (226, 263.4)	95.6% (94.8%, 96.4%)	25% (23.3%, 26.7%)
			Medium	58% (-42.7%, 96.2%)	-2 (-2.6, -1.3)	262.4 (241.4, 283.5)	96.1% (95.3%, 96.8%)	27.6% (25.8%, 29.3%)
			High	57.7% (-26.8%, 97.2%)	-2.3 (-3, -1.7)	264.7 (244.6, 284.8)	95.3% (94.5%, 96.1%)	27.2% (25.5%, 28.9%)
			Very High	58.4% (-24%, 95.4%)	-1.6 (-2.2, -1)	247.2 (227.8, 266.7)	95.4% (94.6%, 96.3%)	25.4% (23.7%, 27.1%)
20%	Superior	Yes	Very Low	55.9% (-230.7%, 100%)	-4.1 (-5.1, -3.1)	628.6 (539, 718.3)	96.9% (96.2%, 97.6%)	57.6% (55.7%, 59.6%)
			Low	58.7% (-33.1%, 95.9%)	-1.3 (-1.9, -0.7)	222.4 (204.5, 240.3)	96.4% (95.7%, 97.1%)	21.8% (20.2%, 23.4%)
			Medium	57.9% (-29.6%, 96.3%)	-2.1 (-2.7, -1.5)	265.9 (245.8, 286)	95.8% (95%, 96.5%)	28.5% (26.8%, 30.3%)
			High	58.4% (-34.1%, 92.7%)	-1.6 (-2.2, -1)	232.9 (213.2, 252.6)	96.2% (95.5%, 96.9%)	22% (20.3%, 23.6%)
			Very High	58.4% (-21.5%, 95.8%)	-1.6 (-2.2, -1)	235 (216.7, 253.3)	95% (94.1%, 95.9%)	22.3% (20.7%, 24%)
	Similar	No	Very Low	55.6% (-294.1%, 100%)	-4.4 (-5.4, -3.4)	671.9 (550.4, 793.3)	96.2% (95.5%, 96.9%)	56.8% (54.9%, 58.8%)
			Low	58.2% (-46.5%, 92.5%)	-1.8 (-2.4, -1.2)	241.8 (221.9, 261.8)	96% (95.3%, 96.8%)	23.3% (21.7%, 25%)
			Medium	58% (-84.4%, 95.2%)	-2 (-2.6, -1.3)	263.9 (239.5, 288.4)	95.6% (94.8%, 96.4%)	27.6% (25.8%, 29.4%)
			High	58.3% (-20.1%, 94.3%)	-1.7 (-2.3, -1.1)	236.8 (218.8, 254.9)	95.4% (94.5%, 96.2%)	22.8% (21.1%, 24.4%)

			Very High	59.3% (-54.3%, 94.6%)	-0.7 (-1.3, -0.2)	221.3 (203.3, 239.3)	95.3% (94.4%, 96.1%)	20.8% (19.2%, 22.4%)
		Yes	Very Low	56.2% (-148.7%, 100%)	-3.8 (-4.7, -2.8)	588.8 (519.5, 658.1)	97.2% (96.6%, 97.8%)	56.8% (54.9%, 58.8%)
			Low	58.1% (-27%, 93.6%)	-1.9 (-2.5, -1.3)	244.8 (225.6, 264)	95.9% (95.1%, 96.7%)	24.1% (22.4%, 25.8%)
			Medium	58.1% (-28.8%, 91.5%)	-1.9 (-2.6, -1.3)	274.1 (251.9, 296.3)	94.9% (94%, 95.7%)	27.6% (25.8%, 29.4%)
			High	58.1% (-45.4%, 97.4%)	-1.9 (-2.5, -1.3)	235.8 (216.7, 254.9)	95.8% (95%, 96.5%)	23.8% (22.1%, 25.5%)
			Very High	58.5% (-26.5%, 94.5%)	-1.5 (-2, -0.9)	212.4 (195.5, 229.2)	96.3% (95.6%, 97.1%)	21.2% (19.6%, 22.8%)
	Inferior	No	Very Low	56% (-97.8%, 100%)	-4 (-5, -3.1)	589.1 (533.4, 644.8)	96.7% (96%, 97.4%)	56.8% (54.8%, 58.7%)
			Low	58.4% (-16.2%, 94.4%)	-1.6 (-2.2, -1)	229.1 (212.8, 245.4)	96.4% (95.7%, 97.2%)	23.9% (22.2%, 25.6%)
			Medium	58.2% (-40.2%, 93.9%)	-1.8 (-2.4, -1.1)	253.6 (233.3, 273.8)	96.2% (95.4%, 96.9%)	27.2% (25.5%, 29%)
			High	58.5% (-31.2%, 97.2%)	-1.5 (-2.1, -0.9)	235.8 (215, 256.7)	95.9% (95.1%, 96.7%)	22.6% (21%, 24.2%)
			Very High	58.5% (-27.3%, 92.2%)	-1.5 (-2.1, -1)	207.7 (192.1, 223.3)	96.4% (95.6%, 97.1%)	21.7% (20.1%, 23.3%)
		Yes	Very Low	55.9% (-98%, 100%)	-4.1 (-5, -3.2)	591 (535.8, 646.2)	96.8% (96.1%, 97.5%)	57% (55%, 58.9%)
			Low	58.6% (-21.5%, 94.2%)	-1.4 (-2, -0.8)	230 (213.6, 246.3)	96.3% (95.5%, 97%)	23.5% (21.8%, 25.1%)
			Medium	58.1% (-28.1%, 93.9%)	-1.9 (-2.6, -1.3)	261.3 (240.6, 282.1)	95.7% (94.9%, 96.5%)	28% (26.2%, 29.7%)
			High	58.4% (-24.7%, 94.9%)	-1.6 (-2.2, -1)	234.6 (215.1, 254.1)	95.7% (94.9%, 96.5%)	22.1% (20.5%, 23.7%)
			Very High	58.7% (-10.1%, 91.2%)	-1.3 (-1.8, -0.7)	210.4 (195.4, 225.4)	96.3% (95.5%, 97%)	21.4% (19.8%, 23%)

Table K. Mean estimate, bias, mean squared error (MSE), coverage, and false negative rate for vaccine efficacy (VE) estimates against severe *Shigella* diarrhea from realistically sized trials for all 50 simulation scenarios when using symptom-based reporting and a single outcome regression model. Hybrid immunity was either not possible (no) or possible such that the first post-vaccination infection conferred additional immunity (yes). Bias was calculated as the average VE estimate for a set of simulations minus the true VE (60%). MSE was calculated as the average of the square of the estimate minus the true parameter. Coverage represents the percent of 95% confidence intervals (CIs) that contained the true VE (60%), and the false negative rate indicates the percent of 95% CIs that incorrectly indicated a non-significant effect.

VE against infection	Strength of infection-acquired immunity	Hybrid immunity	Force of infection	Mean VE estimate (range)	Bias (95% CI)	MSE (95% CI)	Coverage (95% CI)	False negatives (95% CI)
0%	Superior	Yes	Very Low	51.7% (-132.2%, 100%)	-8.3 (-9.4, -7.3)	740.7 (662.3, 819.2)	95.4% (94.5%, 96.2%)	63.1% (61.2%, 65%)
			Low	54% (-41.4%, 94.6%)	-6 (-6.7, -5.4)	301.2 (275, 327.4)	94.5% (93.6%, 95.4%)	30.4% (28.6%, 32.2%)
			Medium	52.4% (-62.7%, 92.2%)	-7.6 (-8.3, -6.9)	363.5 (332.9, 394.1)	93% (92%, 94%)	37.5% (35.6%, 39.4%)
			High	50.3% (-28.1%, 91.8%)	-9.7 (-10.3, -9.1)	358.1 (332.3, 383.9)	90.8% (89.7%, 92%)	35.7% (33.8%, 37.6%)
			Very High	47.8% (-45.3%, 83.7%)	-12.2 (-12.9, -11.6)	418.8 (389.1, 448.5)	87.9% (86.6%, 89.2%)	38% (36.1%, 39.9%)
	Similar	No	Very Low	56.3% (-99.6%, 100%)	-3.7 (-4.6, -2.8)	541.3 (491.7, 590.8)	96.9% (96.2%, 97.6%)	55.9% (53.9%, 57.8%)
			Low	55.3% (-61.1%, 91.6%)	-4.7 (-5.3, -4.1)	262 (240, 283.9)	95.2% (94.4%, 96%)	26.9% (25.2%, 28.7%)
			Medium	54.8% (-43.7%, 91.8%)	-5.2 (-5.8, -4.5)	285.2 (262.5, 308)	94.3% (93.4%, 95.2%)	31% (29.2%, 32.8%)
			High	50.3% (-44.6%, 86.8%)	-9.7 (-10.3, -9.1)	313 (290.2, 335.8)	89.5% (88.3%, 90.7%)	27.7% (25.9%, 29.4%)
			Very High	46.4% (-17.1%, 81.9%)	-13.6 (-14.1, -13.1)	353.7 (332.4, 374.9)	78.8% (77.2%, 80.4%)	21.4% (19.8%, 23%)
		Yes	Very Low	57.6% (-59.7%, 100%)	-2.4 (-3.3, -1.5)	521.1 (476, 566.2)	97.7% (97.1%, 98.3%)	54% (52.1%, 56%)
			Low	59.3% (-9.9%, 94.6%)	-0.7 (-1.2, -0.1)	195.6 (182, 209.1)	96.7% (96%, 97.4%)	20.1% (18.5%, 21.7%)
			Medium	58.8% (-48.9%, 94.3%)	-1.2 (-1.8, -0.6)	244.2 (222.4, 266)	95.7% (94.9%, 96.5%)	22.7% (21.1%, 24.4%)
			High	58.5% (-29.5%, 92.9%)	-1.5 (-2, -0.9)	207.2 (191, 223.4)	95.3% (94.4%, 96.1%)	18.3% (16.8%, 19.8%)
			Very High	57.7% (-17.6%, 91.8%)	-2.3 (-2.8, -1.7)	199.8 (185.5, 214)	95.2% (94.4%, 96.1%)	17.9% (16.4%, 19.4%)

	Inferior	No	Very Low	56.7% (-119.3%, 100%)	-3.3 (-4.2, -2.4)	528.1 (477.6, 578.7)	97.2% (96.6%, 97.9%)	55.5% (53.6%, 57.5%)
			Low	56.9% (-19.6%, 88.7%)	-3.1 (-3.6, -2.5)	233.6 (216.1, 251)	95.6% (94.8%, 96.4%)	23.2% (21.6%, 24.9%)
			Medium	56.3% (-11.1%, 93.9%)	-3.7 (-4.3, -3.1)	254.2 (235.4, 273.1)	94.9% (94%, 95.7%)	27.1% (25.3%, 28.8%)
			High	54.1% (-23.9%, 88.5%)	-5.9 (-6.5, -5.3)	244.8 (226.4, 263.2)	93.1% (92.1%, 94.1%)	22.5% (20.9%, 24.2%)
			Very High	52.9% (-4.3%, 87%)	-7.1 (-7.6, -6.6)	224.8 (209.4, 240.2)	92.3% (91.3%, 93.4%)	18% (16.5%, 19.5%)
		Yes	Very Low	57.1% (-119.3%, 100%)	-2.9 (-3.8, -2)	526.8 (477.2, 576.4)	97.2% (96.6%, 97.8%)	54.5% (52.6%, 56.5%)
			Low	58.7% (-19.6%, 91.4%)	-1.3 (-1.9, -0.8)	216.7 (200.3, 233.2)	95.7% (94.9%, 96.5%)	19.8% (18.2%, 21.3%)
			Medium	58.3% (-25.1%, 95.2%)	-1.7 (-2.3, -1.1)	228.5 (211.5, 245.6)	95.3% (94.5%, 96.1%)	22.2% (20.6%, 23.8%)
			High	58% (-12.3%, 88.6%)	-2 (-2.5, -1.4)	193.7 (179.2, 208.2)	95.3% (94.5%, 96.1%)	16% (14.6%, 17.4%)
			Very High	58.1% (-13.6%, 92.9%)	-1.9 (-2.4, -1.4)	167.4 (154.2, 180.7)	95% (94.1%, 95.9%)	11.1% (9.9%, 12.4%)
20%	Superior	Yes	Very Low	55.5% (-233%, 100%)	-4.5 (-5.5, -3.6)	623.4 (536.7, 710.2)	96.6% (95.9%, 97.3%)	57.7% (55.7%, 59.6%)
			Low	57.2% (-42.4%, 89.7%)	-2.8 (-3.4, -2.2)	233 (213.3, 252.7)	96.3% (95.6%, 97.1%)	23.8% (22.1%, 25.5%)
			Medium	56% (-49.4%, 96.2%)	-4 (-4.7, -3.4)	283.4 (260.5, 306.3)	95.1% (94.3%, 96%)	30.4% (28.6%, 32.2%)
			High	53.6% (-48.9%, 88.3%)	-6.4 (-7, -5.8)	280 (255.7, 304.2)	94% (93.1%, 95%)	28.1% (26.4%, 29.9%)
			Very High	50.9% (-48.5%, 87.9%)	-9.1 (-9.7, -8.4)	336.5 (309.8, 363.2)	91% (89.8%, 92.1%)	30.7% (28.9%, 32.5%)
	Similar	No	Very Low	54.8% (-198.3%, 100%)	-5.2 (-6.2, -4.2)	651.2 (570.2, 732.3)	96.3% (95.5%, 97%)	57.7% (55.7%, 59.6%)
			Low	55.4% (-39.3%, 90.5%)	-4.6 (-5.2, -4)	264.3 (242, 286.7)	95% (94.1%, 95.9%)	27% (25.3%, 28.8%)
			Medium	54.6% (-51%, 93.3%)	-5.4 (-6.1, -4.8)	298.8 (274.9, 322.7)	95% (94.2%, 95.9%)	32.6% (30.8%, 34.4%)
			High	50.1% (-42.1%, 91.1%)	-9.9 (-10.6, -9.3)	355.5 (329.4, 381.6)	90.2% (89.1%, 91.4%)	33.4% (31.5%, 35.2%)

			Very High	46.8% (-50.4%, 86.1%)	-13.2 (-13.8, -12.6)	424.9 (396.6, 453.2)	84.2% (82.8%, 85.7%)	35.8% (33.9%, 37.7%)
		Yes	Very Low	56.2% (-148.5%, 100%)	-3.8 (-4.7, -2.9)	571.2 (509.7, 632.7)	97% (96.3%, 97.6%)	56.5% (54.6%, 58.5%)
			Low	57.9% (-32.7%, 91.5%)	-2.1 (-2.7, -1.5)	238.3 (218.1, 258.6)	95.2% (94.4%, 96%)	22.6% (21%, 24.2%)
			Medium	57.7% (-28.9%, 92.4%)	-2.3 (-2.9, -1.7)	257.8 (237.6, 278)	95.5% (94.7%, 96.3%)	25.4% (23.7%, 27.1%)
			High	57% (-18.7%, 93%)	-3 (-3.6, -2.4)	220.3 (204.2, 236.3)	94.9% (94.1%, 95.8%)	21.1% (19.5%, 22.7%)
			Very High	56% (-7.5%, 95.1%)	-4 (-4.5, -3.5)	203.4 (188.6, 218.1)	95.2% (94.3%, 96%)	19.3% (17.8%, 20.9%)
	Inferior	No	Very Low	55.6% (-131.5%, 100%)	-4.4 (-5.3, -3.5)	594.8 (533.1, 656.5)	96.4% (95.7%, 97.2%)	56.4% (54.4%, 58.3%)
			Low	57.1% (-18.8%, 92.1%)	-2.9 (-3.5, -2.3)	225.7 (208.6, 242.9)	95.6% (94.8%, 96.4%)	22.3% (20.7%, 24%)
			Medium	56.6% (-20.3%, 90.3%)	-3.4 (-4, -2.8)	246.8 (228.6, 265)	95.7% (94.9%, 96.5%)	26.1% (24.4%, 27.8%)
			High	54.9% (-27.6%, 92.8%)	-5.1 (-5.6, -4.5)	228.7 (210.5, 246.9)	94.6% (93.7%, 95.4%)	20.8% (19.2%, 22.4%)
			Very High	52.1% (-10.2%, 84%)	-7.9 (-8.4, -7.3)	245.7 (229, 262.5)	91.5% (90.4%, 92.6%)	21.3% (19.7%, 22.9%)
		Yes	Very Low	55.9% (-98.7%, 100%)	-4.1 (-5.1, -3.2)	582.9 (526.7, 639.1)	96.5% (95.8%, 97.2%)	55.6% (53.7%, 57.5%)
			Low	58.4% (-17.9%, 92.7%)	-1.6 (-2.2, -1)	212.6 (197, 228.2)	96% (95.2%, 96.8%)	20.5% (18.9%, 22.1%)
			Medium	58% (-14%, 91.3%)	-2 (-2.6, -1.4)	232.7 (215.9, 249.4)	95.6% (94.8%, 96.4%)	24.1% (22.4%, 25.8%)
			High	58% (-27.1%, 95.5%)	-2 (-2.5, -1.5)	183.7 (168.7, 198.7)	95.9% (95.1%, 96.7%)	14.9% (13.5%, 16.3%)
			Very High	56.8% (-26.1%, 88.6%)	-3.2 (-3.7, -2.7)	174 (160.8, 187.2)	94.8% (94%, 95.7%)	12.8% (11.5%, 14.1%)

Table L. Mean estimate, bias, mean squared error (MSE), coverage, and false negative rate for vaccine efficacy (VE) estimates against severe *Shigella* diarrhea from realistically sized trials for all 50 simulation scenarios when using active surveillance for infection and a stratified recurrent outcome regression model. Hybrid immunity was either not possible (no) or possible such that the first post-vaccination infection conferred additional immunity (yes). Bias was calculated as the average VE estimate for a set of simulations minus the true VE (60%). MSE was calculated as the average of the square of the estimate minus the true parameter. Coverage represents the percent of 95% confidence intervals (CIs) that contained the true VE (60%), and the false negative rate indicates the percent of 95% CIs that incorrectly indicated a non-significant effect.

VE against infection	Strength of infection-acquired immunity	Hybrid immunity	Force of infection	Mean VE estimate (range)	Bias (95% CI)	MSE (95% CI)	Coverage (95% CI)	False negatives (95% CI)
0%	Superior	Yes	Very Low	51.2% (-133.8%, 100%)	-8.8 (-9.8, -7.8)	759.8 (679.5, 840.1)	94.7% (93.8%, 95.6%)	63% (61.1%, 64.9%)
			Low	52.7% (-40.9%, 94.4%)	-7.3 (-8, -6.7)	328.2 (299.8, 356.6)	93.8% (92.9%, 94.8%)	32.2% (30.4%, 34.1%)
			Medium	50.8% (-53.7%, 88.2%)	-9.2 (-9.9, -8.5)	395.6 (364.9, 426.3)	91.6% (90.5%, 92.6%)	39.9% (38%, 41.8%)
			High	46.7% (-40.4%, 90.5%)	-13.3 (-14, -12.6)	459.9 (429, 490.7)	86.1% (84.8%, 87.5%)	41.8% (39.9%, 43.8%)
			Very High	42.4% (-34.6%, 81.2%)	-17.6 (-18.2, -16.9)	593.7 (558.8, 628.6)	77.5% (75.9%, 79.2%)	46% (44.1%, 48%)
	Similar	No	Very Low	55.9% (-101.5%, 100%)	-4.1 (-5.1, -3.2)	548.5 (497.6, 599.4)	96.5% (95.8%, 97.2%)	55.7% (53.7%, 57.6%)
			Low	53.9% (-65.1%, 91.4%)	-6.1 (-6.7, -5.5)	284.1 (260.4, 307.8)	94% (93.1%, 94.9%)	28.9% (27.1%, 30.7%)
			Medium	52.9% (-31.9%, 91.8%)	-7.1 (-7.7, -6.4)	319 (294.6, 343.4)	93.8% (92.8%, 94.7%)	33% (31.2%, 34.8%)
			High	45.9% (-51.6%, 83.7%)	-14.1 (-14.7, -13.5)	424.4 (397.9, 450.9)	81.4% (79.9%, 83%)	34.6% (32.7%, 36.4%)
			Very High	39.6% (-23.3%, 73.1%)	-20.4 (-20.9, -19.9)	596.9 (568.7, 625.1)	56.8% (54.8%, 58.7%)	31.5% (29.7%, 33.3%)
		Yes	Very Low	57.4% (-59.8%, 100%)	-2.6 (-3.5, -1.7)	521.9 (477, 566.8)	97.4% (96.7%, 98%)	53.5% (51.5%, 55.4%)
			Low	58.8% (-12.7%, 94.5%)	-1.2 (-1.8, -0.7)	196.6 (183, 210.1)	96.6% (95.9%, 97.3%)	20.2% (18.7%, 21.8%)
			Medium	58.1% (-43.7%, 94.4%)	-1.9 (-2.5, -1.3)	245.3 (224.1, 266.5)	95.8% (95%, 96.5%)	23.5% (21.8%, 25.1%)
			High	56.5% (-20.5%, 93.4%)	-3.5 (-4.1, -3)	222.3 (205.1, 239.4)	94.6% (93.7%, 95.5%)	20.4% (18.8%, 21.9%)
			Very High	54.3% (-9%, 91.3%)	-5.7 (-6.3, -5.1)	238.5 (221.7, 255.2)	93.2% (92.2%, 94.2%)	21.8% (20.1%, 23.4%)

	Inferior	No	Very Low	56.5% (-120.5%, 100%)	-3.5 (-4.4, -2.6)	525.9 (474.8, 576.9)	97% (96.4%, 97.7%)	54.6% (52.6%, 56.6%)
			Low	56.2% (-18.6%, 89.8%)	-3.8 (-4.4, -3.2)	236.6 (218.5, 254.7)	95.2% (94.4%, 96.1%)	23% (21.4%, 24.6%)
			Medium	55.4% (-18.1%, 94.6%)	-4.6 (-5.2, -4)	260.3 (240.7, 279.8)	94.2% (93.3%, 95.1%)	27.4% (25.6%, 29.1%)
			High	51.7% (-29.9%, 84.8%)	-8.3 (-8.8, -7.7)	273.6 (253.6, 293.7)	90.4% (89.3%, 91.6%)	22.9% (21.3%, 24.6%)
			Very High	48.7% (-11.5%, 83.8%)	-11.3 (-11.8, -10.8)	293.2 (274.9, 311.4)	85.9% (84.5%, 87.2%)	20.4% (18.8%, 22%)
		Yes	Very Low	57.1% (-120.5%, 100%)	-2.9 (-3.8, -2)	521.6 (471.7, 571.4)	97% (96.3%, 97.6%)	53.4% (51.4%, 55.4%)
		Yes	Low	58.4% (-16.5%, 91.1%)	-1.6 (-2.2, -1)	211.2 (195.1, 227.4)	95.6% (94.8%, 96.4%)	19.4% (17.9%, 21%)
			Medium	58% (-19.8%, 95%)	-2 (-2.6, -1.4)	224.3 (207.5, 241)	95% (94.2%, 95.9%)	21.1% (19.5%, 22.7%)
			High	56.8% (-3.9%, 88.2%)	-3.2 (-3.7, -2.7)	190.1 (176.3, 203.9)	94.5% (93.6%, 95.4%)	15.2% (13.8%, 16.6%)
			Very High	55.6% (-14%, 90.7%)	-4.4 (-4.9, -3.9)	167.2 (154.7, 179.7)	93.9% (93%, 94.9%)	10% (8.9%, 11.2%)
20%	Superior	Yes	Very Low	55.5% (-230.7%, 100%)	-4.5 (-5.4, -3.5)	615.9 (529.9, 701.9)	96.2% (95.5%, 97%)	57.1% (55.2%, 59.1%)
			Low	57% (-42.6%, 89.8%)	-3 (-3.6, -2.4)	231.8 (212.3, 251.4)	96.2% (95.5%, 97%)	23.5% (21.8%, 25.1%)
			Medium	55.8% (-43.2%, 96.3%)	-4.2 (-4.9, -3.6)	277.9 (256.2, 299.6)	95.1% (94.3%, 96%)	29.4% (27.6%, 31.2%)
			High	53% (-39.3%, 89.2%)	-7 (-7.6, -6.4)	283.2 (260.3, 306.1)	93% (92%, 94%)	27.5% (25.8%, 29.3%)
			Very High	49.5% (-37.8%, 85.5%)	-10.5 (-11.1, -9.9)	357 (330.7, 383.3)	88.8% (87.6%, 90%)	31.4% (29.6%, 33.2%)
	Similar	No	Very Low	54.6% (-199.6%, 100%)	-5.4 (-6.4, -4.4)	651.6 (569.6, 733.7)	95.9% (95.1%, 96.7%)	57.9% (55.9%, 59.8%)
			Low	54.6% (-40.8%, 90.6%)	-5.4 (-6, -4.7)	273.7 (250.6, 296.7)	94.6% (93.7%, 95.4%)	28.5% (26.7%, 30.2%)
			Medium	53.3% (-38.9%, 93.2%)	-6.7 (-7.3, -6)	319.6 (294.8, 344.3)	94% (93%, 94.9%)	33.2% (31.4%, 35.1%)
			High	47.1% (-30.1%, 88.7%)	-12.9 (-13.5, -12.2)	434.6 (404.9, 464.3)	86.3% (84.9%, 87.6%)	37.5% (35.6%, 39.4%)

			Very High	41.9% (-35.1%, 81.7%)	-18.1 (-18.7, -17.5)	582.8 (550, 615.7)	73.2% (71.4%, 74.9%)	43.2% (41.3%, 45.1%)
		Yes	Very Low	56.4% (-148.7%, 100%)	-3.6 (-4.5, -2.7)	563.4 (502, 624.8)	96.7% (96%, 97.4%)	55.9% (53.9%, 57.8%)
			Low	58.1% (-33.6%, 91.6%)	-1.9 (-2.5, -1.3)	229.4 (210.5, 248.3)	95.6% (94.8%, 96.4%)	22.3% (20.6%, 23.9%)
			Medium	58.1% (-14.8%, 92.2%)	-1.9 (-2.5, -1.3)	241.4 (223.7, 259.1)	95.5% (94.7%, 96.3%)	24.2% (22.5%, 25.8%)
			High	57.4% (-5.5%, 91.6%)	-2.6 (-3.1, -2)	202.3 (188.2, 216.4)	95% (94.1%, 95.9%)	18.3% (16.8%, 19.8%)
			Very High	56.3% (-4.3%, 93.7%)	-3.7 (-4.2, -3.2)	186.2 (173, 199.4)	94.9% (94%, 95.7%)	16.1% (14.6%, 17.5%)
	Inferior	No	Very Low	55.6% (-134.6%, 100%)	-4.4 (-5.3, -3.5)	588.7 (527.7, 649.8)	95.9% (95.1%, 96.7%)	55.9% (53.9%, 57.8%)
			Low	56.7% (-14.8%, 92.4%)	-3.3 (-3.8, -2.7)	223.8 (207.1, 240.5)	95.1% (94.3%, 96%)	21.3% (19.7%, 22.9%)
			Medium	56.1% (-25%, 90.3%)	-3.9 (-4.5, -3.3)	246.7 (228, 265.4)	95.1% (94.2%, 95.9%)	24.6% (22.9%, 26.3%)
			High	53.5% (-18%, 87.9%)	-6.5 (-7, -6)	226.7 (210.1, 243.2)	92.8% (91.7%, 93.8%)	19.6% (18.1%, 21.2%)
			Very High	49.6% (-4.8%, 82.7%)	-10.4 (-10.9, -9.8)	274.3 (257, 291.6)	87.2% (85.9%, 88.5%)	19.2% (17.6%, 20.7%)
		Yes	Very Low	56% (-100.4%, 100%)	-4 (-4.9, -3)	568.3 (514.1, 622.5)	96.1% (95.4%, 96.9%)	55% (53%, 56.9%)
			Low	58.5% (-10.1%, 92.9%)	-1.5 (-2, -0.9)	204.3 (189.6, 219.1)	95.9% (95.1%, 96.7%)	18.4% (16.9%, 20%)
			Medium	58.1% (-17.8%, 91.3%)	-1.9 (-2.4, -1.3)	223.1 (207, 239.2)	95.3% (94.5%, 96.1%)	21.6% (20%, 23.2%)
			High	57.9% (-0.1%, 93.6%)	-2.1 (-2.6, -1.6)	160.8 (149.1, 172.5)	95.8% (95%, 96.5%)	11.6% (10.4%, 12.9%)
			Very High	56.2% (-19.4%, 89.7%)	-3.8 (-4.3, -3.3)	158.4 (146.5, 170.3)	94.5% (93.6%, 95.4%)	9.4% (8.3%, 10.6%)

Table M. Mean estimate, bias, mean squared error (MSE), coverage, and false negative rate for vaccine efficacy (VE) estimates against severe *Shigella* diarrhea from realistically sized trials for all 50 simulation scenarios when using symptom-based reporting and a stratified recurrent outcome regression model. Hybrid immunity was either not possible (no) or possible such that the first post-vaccination infection conferred additional immunity (yes). Bias was calculated as the average VE estimate for a set of simulations minus the true VE (60%). MSE was calculated as the average of the square of the estimate minus the true parameter. Coverage represents the percent of 95% confidence intervals (CIs) that contained the true VE (60%), and the false negative rate indicates the percent of 95% CIs that incorrectly indicated a non-significant effect.

VE against infection	Strength of infection-acquired immunity	Hybrid immunity	Force of infection	Mean VE estimate (range)	Bias (95% CI)	MSE (95% CI)	Coverage (95% CI)	False negatives (95% CI)
0%	Superior	Yes	Very Low	51.4% (-132.2%, 100%)	-8.6 (-9.6, -7.6)	749.9 (670.6, 829.2)	94.8% (93.9%, 95.7%)	63.2% (61.3%, 65.1%)
			Low	53.2% (-40%, 94.6%)	-6.8 (-7.4, -6.1)	314 (286.6, 341.4)	94.3% (93.4%, 95.2%)	30.9% (29.1%, 32.7%)
			Medium	51.5% (-52.3%, 88.5%)	-8.5 (-9.2, -7.8)	376.9 (347.3, 406.5)	92% (90.9%, 93.1%)	38.8% (36.9%, 40.7%)
			High	48.1% (-36.6%, 90.9%)	-11.9 (-12.6, -11.3)	413.4 (384.9, 441.9)	87.8% (86.6%, 89.1%)	38.6% (36.7%, 40.5%)
			Very High	44.2% (-34.6%, 82.2%)	-15.8 (-16.5, -15.2)	522.1 (490, 554.2)	80.9% (79.4%, 82.5%)	42% (40%, 43.9%)
	Similar	No	Very Low	56% (-99.6%, 100%)	-4 (-4.9, -3.1)	544.7 (493.9, 595.5)	96.5% (95.8%, 97.2%)	55.8% (53.9%, 57.7%)
			Low	54.3% (-61.1%, 91.6%)	-5.7 (-6.3, -5.1)	276.6 (253.7, 299.5)	94.4% (93.5%, 95.3%)	28.2% (26.4%, 29.9%)
			Medium	53.5% (-31%, 91.9%)	-6.5 (-7.2, -5.9)	307.9 (284.2, 331.6)	94% (93%, 94.9%)	32.1% (30.3%, 33.9%)
			High	46.9% (-49.2%, 83.4%)	-13.1 (-13.7, -12.5)	392.3 (367.1, 417.6)	84% (82.6%, 85.4%)	32.6% (30.7%, 34.4%)
			Very High	40.8% (-19%, 73.8%)	-19.2 (-19.7, -18.7)	545.1 (518.4, 571.9)	60.4% (58.5%, 62.3%)	29.4% (27.6%, 31.2%)
		Yes	Very Low	57.6% (-59.7%, 100%)	-2.4 (-3.3, -1.5)	516.4 (471.9, 560.9)	97.4% (96.7%, 98%)	53.6% (51.6%, 55.5%)
			Low	59.3% (-9.9%, 94.6%)	-0.7 (-1.2, -0.1)	190.8 (177.8, 203.7)	96.6% (95.9%, 97.3%)	19% (17.5%, 20.6%)
			Medium	58.8% (-44.4%, 94.4%)	-1.2 (-1.8, -0.6)	234.8 (214.6, 255)	95.8% (95.1%, 96.6%)	21.6% (20%, 23.2%)
			High	57.9% (-15.3%, 93.5%)	-2.1 (-2.7, -1.6)	201.9 (186.6, 217.3)	95% (94.2%, 95.9%)	17.8% (16.3%, 19.3%)
			Very High	56.2% (-5.7%, 91.6%)	-3.8 (-4.3, -3.2)	205.6 (191.2, 220)	95% (94.1%, 95.9%)	18.2% (16.6%, 19.7%)

	Inferior	No	Very Low	56.6% (-119.3%, 100%)	-3.4 (-4.3, -2.5)	523.3 (472.8, 573.9)	97% (96.4%, 97.7%)	55.1% (53.1%, 57%)
			Low	56.4% (-17.2%, 89.5%)	-3.6 (-4.1, -3)	234 (216.2, 251.8)	95.4% (94.5%, 96.2%)	22.8% (21.1%, 24.4%)
			Medium	55.7% (-16.6%, 94.4%)	-4.3 (-4.9, -3.7)	257.4 (238, 276.7)	94.1% (93.2%, 95%)	27.1% (25.3%, 28.8%)
			High	52.4% (-24.7%, 85.2%)	-7.6 (-8.2, -7.1)	259.6 (240.4, 278.8)	91% (89.9%, 92.1%)	21.6% (19.9%, 23.2%)
			Very High	49.6% (-13.9%, 85%)	-10.4 (-10.9, -9.9)	270.9 (253.4, 288.3)	87.5% (86.2%, 88.8%)	18.7% (17.2%, 20.2%)
		Yes	Very Low	57.2% (-119.3%, 100%)	-2.8 (-3.7, -1.9)	518.6 (469.1, 568)	97% (96.3%, 97.6%)	54% (52%, 56%)
			Low	58.6% (-14.7%, 91.4%)	-1.4 (-1.9, -0.8)	208.6 (192.9, 224.4)	95.7% (94.9%, 96.5%)	19.1% (17.5%, 20.6%)
			Medium	58.4% (-17.8%, 95.2%)	-1.6 (-2.2, -1.1)	220.4 (204.1, 236.8)	95.2% (94.3%, 96%)	20.6% (19%, 22.2%)
			High	57.6% (-4.8%, 88.3%)	-2.4 (-3, -1.9)	181.1 (168.1, 194.2)	94.7% (93.8%, 95.6%)	13.8% (12.5%, 15.2%)
			Very High	56.8% (-10.9%, 91.4%)	-3.2 (-3.7, -2.8)	153.2 (141.7, 164.8)	94.4% (93.5%, 95.3%)	8.9% (7.8%, 10%)
20%	Superior	Yes	Very Low	55.4% (-233%, 100%)	-4.6 (-5.5, -3.6)	619.5 (532.7, 706.3)	96.2% (95.5%, 96.9%)	57.2% (55.2%, 59.1%)
			Low	56.7% (-42.4%, 89.7%)	-3.3 (-3.9, -2.7)	236.8 (216.8, 256.8)	96% (95.3%, 96.8%)	24% (22.3%, 25.6%)
			Medium	55.4% (-41.6%, 96.2%)	-4.6 (-5.3, -4)	286.4 (263.9, 308.8)	94.9% (94%, 95.7%)	30.3% (28.5%, 32.1%)
			High	52% (-40.6%, 88.9%)	-8 (-8.6, -7.3)	306.5 (281.9, 331.1)	92.2% (91.1%, 93.3%)	30% (28.2%, 31.8%)
			Very High	48% (-40.9%, 85.5%)	-12 (-12.7, -11.4)	404.9 (376.1, 433.7)	86.6% (85.3%, 87.9%)	34.9% (33.1%, 36.8%)
	Similar	No	Very Low	54.5% (-198.3%, 100%)	-5.5 (-6.4, -4.5)	653.2 (571.9, 734.5)	95.8% (95%, 96.6%)	58.1% (56.2%, 60.1%)
			Low	54.4% (-42.1%, 90.6%)	-5.6 (-6.2, -4.9)	278.6 (255.1, 302.2)	94.4% (93.5%, 95.3%)	28.7% (26.9%, 30.5%)
			Medium	53.1% (-40.7%, 93.3%)	-6.9 (-7.6, -6.3)	325.7 (300.4, 351.1)	93.7% (92.8%, 94.7%)	33.7% (31.9%, 35.6%)
			High	46.6% (-31.2%, 88.2%)	-13.4 (-14.1, -12.8)	452.4 (421.7, 483.1)	85.6% (84.2%, 87%)	38.8% (36.9%, 40.7%)

			Very High	41.1% (-37.3%, 81.8%)	-18.9 (-19.5, -18.3)	617.1 (582.9, 651.2)	71.8% (70%, 73.5%)	45% (43%, 47%)
		Yes	Very Low	56.3% (-148.5%, 100%)	-3.7 (-4.6, -2.8)	566.9 (505.5, 628.3)	96.6% (95.9%, 97.3%)	56% (54%, 57.9%)
			Low	57.9% (-37.8%, 91.5%)	-2.1 (-2.7, -1.6)	233.8 (214.3, 253.4)	95.6% (94.8%, 96.4%)	23% (21.3%, 24.6%)
			Medium	57.8% (-16.8%, 92.4%)	-2.2 (-2.8, -1.6)	247.6 (229.3, 265.9)	95.5% (94.7%, 96.3%)	24.9% (23.2%, 26.6%)
			High	56.6% (-8.2%, 91.6%)	-3.4 (-3.9, -2.8)	213.4 (198.4, 228.4)	94.7% (93.8%, 95.6%)	20.2% (18.7%, 21.8%)
			Very High	55% (-4.9%, 93.3%)	-5 (-5.6, -4.5)	207.7 (192.9, 222.6)	94.7% (93.8%, 95.6%)	18.3% (16.8%, 19.8%)
	Inferior	No	Very Low	55.5% (-131.5%, 100%)	-4.5 (-5.4, -3.5)	590.8 (530.1, 651.4)	95.9% (95.1%, 96.7%)	56.1% (54.1%, 58%)
			Low	56.6% (-16.5%, 92.5%)	-3.4 (-3.9, -2.8)	226.3 (209.3, 243.3)	94.9% (94%, 95.7%)	21.6% (20%, 23.2%)
			Medium	56% (-24.8%, 90.3%)	-4 (-4.6, -3.4)	248.8 (230, 267.6)	95.1% (94.2%, 95.9%)	24.8% (23.1%, 26.5%)
			High	53.2% (-19.6%, 88%)	-6.8 (-7.3, -6.3)	233.8 (216.8, 250.8)	92.3% (91.3%, 93.4%)	19.6% (18.1%, 21.2%)
			Very High	49.3% (-6.1%, 83.1%)	-10.7 (-11.3, -10.2)	283.5 (265.8, 301.1)	86.5% (85.2%, 87.9%)	19.9% (18.4%, 21.5%)
		Yes	Very Low	56% (-98.7%, 100%)	-4 (-4.9, -3.1)	570.5 (516.3, 624.7)	96.1% (95.4%, 96.9%)	55.3% (53.3%, 57.2%)
			Low	58.4% (-11.8%, 92.9%)	-1.6 (-2.2, -1)	206.4 (191.4, 221.3)	95.7% (94.9%, 96.5%)	19% (17.5%, 20.6%)
			Medium	58% (-16.8%, 91.2%)	-2 (-2.6, -1.4)	225.4 (209.2, 241.7)	95.5% (94.7%, 96.3%)	22.4% (20.8%, 24%)
			High	57.5% (-3.6%, 93.7%)	-2.5 (-3, -2)	165.8 (153.6, 178)	95.7% (94.9%, 96.5%)	12.3% (11%, 13.6%)
			Very High	55.7% (-21.4%, 88.9%)	-4.3 (-4.7, -3.8)	164.1 (152, 176.3)	94.3% (93.4%, 95.2%)	10.6% (9.4%, 11.8%)

Table N. Mean estimate, bias, mean squared error (MSE), coverage, and false negative rate for vaccine efficacy (VE) estimates against severe *Shigella* diarrhea from realistically sized trials for all simulation scenarios when using active surveillance for infection and a crude recurrent outcome regression model. Hybrid immunity was either not possible (no) or possible such that the first post-vaccination infection conferred additional immunity (yes). Bias was calculated as the average VE estimate for a set of simulations minus the true VE (60%). MSE was calculated as the average of the square of the estimate minus the true parameter. Coverage represents the percent of 95% confidence intervals (CIs) that contained the true VE (60%), and the false negative rate indicates the percent of 95% CIs that incorrectly indicated a non-significant effect.

VE against infection	Strength of infection-acquired immunity	Hybrid immunity	Force of infection	Mean VE estimate (range)	Bias (95% CI)	MSE (95% CI)	Coverage (95% CI)	False negatives (95% CI)
0%	Superior	Yes	Very Low	51.2% (-133.3%, 100%)	-8.8 (-9.8, -7.8)	757.2 (677.2, 837.1)	94.8% (93.9%, 95.7%)	62.9% (61%, 64.8%)
			Low	52.7% (-41.7%, 94.4%)	-7.3 (-8, -6.7)	326.7 (298.5, 354.9)	93.8% (92.9%, 94.7%)	32% (30.2%, 33.8%)
			Medium	50.8% (-53.4%, 88.5%)	-9.2 (-9.9, -8.5)	394.2 (363.5, 424.8)	91.6% (90.5%, 92.6%)	39.6% (37.7%, 41.6%)
			High	46.7% (-41.2%, 90.5%)	-13.3 (-13.9, -12.6)	454.4 (424.2, 484.7)	86% (84.6%, 87.3%)	41.2% (39.3%, 43.1%)
			Very High	42.5% (-37.4%, 81.3%)	-17.5 (-18.2, -16.9)	587.9 (553.3, 622.5)	77.2% (75.6%, 78.8%)	45.5% (43.5%, 47.4%)
	Similar	No	Very Low	55.9% (-100%, 100%)	-4.1 (-5, -3.2)	547.4 (496.8, 598)	96.5% (95.8%, 97.2%)	55.6% (53.7%, 57.5%)
			Low	53.9% (-64.3%, 91.4%)	-6.1 (-6.7, -5.5)	283.2 (259.6, 306.7)	94.1% (93.2%, 95%)	28.3% (26.6%, 30.1%)
			Medium	52.9% (-30.8%, 91.7%)	-7.1 (-7.7, -6.4)	317.9 (293.7, 342.2)	93.9% (92.9%, 94.8%)	32.4% (30.5%, 34.2%)
			High	45.9% (-47.9%, 83.7%)	-14.1 (-14.7, -13.5)	423.3 (396.9, 449.6)	81.2% (79.7%, 82.8%)	34.3% (32.4%, 36.1%)
			Very High	39.6% (-21.7%, 72.4%)	-20.4 (-20.9, -19.9)	593.6 (565.8, 621.5)	56.5% (54.6%, 58.5%)	31.5% (29.7%, 33.3%)
		Yes	Very Low	57.4% (-60%, 100%)	-2.6 (-3.5, -1.7)	520.4 (475.6, 565.2)	97.4% (96.8%, 98%)	53.3% (51.3%, 55.2%)
			Low	58.8% (-11.8%, 94.4%)	-1.2 (-1.8, -0.7)	195.4 (182, 208.9)	96.6% (95.9%, 97.3%)	19.9% (18.4%, 21.5%)
			Medium	58.1% (-42.9%, 94.3%)	-1.9 (-2.5, -1.3)	243.6 (222.6, 264.5)	95.8% (95%, 96.5%)	23% (21.4%, 24.6%)
			High	56.5% (-17.4%, 93.3%)	-3.5 (-4.1, -2.9)	219.4 (202.5, 236.2)	94.9% (94%, 95.7%)	19.8% (18.2%, 21.4%)
			Very High	54.3% (-8.7%, 91.1%)	-5.7 (-6.2, -5.1)	234.7 (218.3, 251.2)	93.4% (92.4%, 94.4%)	21.2% (19.6%, 22.8%)

	Inferior	No	Very Low	56.5% (-120%, 100%)	-3.5 (-4.4, -2.6)	525.1 (474.3, 575.9)	97.2% (96.6%, 97.8%)	54.6% (52.6%, 56.5%)
			Low	56.2% (-19%, 89.7%)	-3.8 (-4.4, -3.2)	236.2 (218.2, 254.2)	95.4% (94.6%, 96.2%)	22.7% (21.1%, 24.4%)
			Medium	55.4% (-17.6%, 94.6%)	-4.6 (-5.2, -4)	259.6 (240.1, 279.1)	94.2% (93.3%, 95.1%)	26.7% (24.9%, 28.4%)
			High	51.7% (-29.5%, 84.6%)	-8.3 (-8.8, -7.7)	272.7 (252.7, 292.8)	90.3% (89.1%, 91.4%)	22.8% (21.1%, 24.4%)
			Very High	48.7% (-11.5%, 83.6%)	-11.3 (-11.8, -10.8)	291.3 (273.2, 309.4)	86% (84.6%, 87.3%)	20.3% (18.7%, 21.9%)
		Yes	Very Low	57.1% (-120%, 100%)	-2.9 (-3.8, -2)	520.7 (470.9, 570.5)	97% (96.4%, 97.7%)	53.3% (51.3%, 55.2%)
			Low	58.4% (-15.8%, 91.2%)	-1.6 (-2.2, -1.1)	210.9 (194.8, 227)	95.7% (94.9%, 96.5%)	19% (17.5%, 20.5%)
			Medium	58% (-20%, 95%)	-2 (-2.6, -1.4)	223.7 (207, 240.5)	95.1% (94.2%, 95.9%)	21% (19.4%, 22.6%)
			High	56.8% (-3.9%, 88.2%)	-3.2 (-3.7, -2.7)	188.7 (175, 202.4)	94.4% (93.5%, 95.3%)	15% (13.6%, 16.4%)
			Very High	55.7% (-14.2%, 90.9%)	-4.3 (-4.8, -3.9)	165.5 (153, 177.9)	93.9% (93%, 94.9%)	9.9% (8.7%, 11%)
20%	Superior	Yes	Very Low	55.2% (-233.4%, 100%)	-4.8 (-5.8, -3.8)	625.6 (538.2, 713)	96.2% (95.5%, 97%)	57.1% (55.1%, 59%)
			Low	56.1% (-46.2%, 89.5%)	-3.9 (-4.5, -3.3)	246.5 (225.6, 267.4)	95.7% (94.9%, 96.5%)	24.5% (22.8%, 26.2%)
			Medium	54.6% (-41.1%, 96.2%)	-5.4 (-6, -4.7)	299.2 (276.1, 322.3)	94.6% (93.7%, 95.5%)	31.5% (29.7%, 33.3%)
			High	50.6% (-46.6%, 88.4%)	-9.4 (-10.1, -8.8)	342.2 (315.5, 369)	90.8% (89.6%, 91.9%)	33.1% (31.2%, 34.9%)
			Very High	46.1% (-44.8%, 84.9%)	-13.9 (-14.6, -13.3)	467.2 (435.3, 499.1)	83.6% (82.2%, 85.1%)	38.6% (36.7%, 40.5%)
	Similar	No	Very Low	54.4% (-200%, 100%)	-5.6 (-6.6, -4.6)	657.9 (575.6, 740.2)	95.8% (95.1%, 96.6%)	57.5% (55.6%, 59.5%)
			Low	54% (-41.6%, 90.5%)	-6 (-6.6, -5.4)	284.4 (260.8, 308.1)	94.4% (93.5%, 95.3%)	29.2% (27.4%, 30.9%)
			Medium	52.6% (-41.6%, 93.1%)	-7.4 (-8.1, -6.7)	334.7 (308.7, 360.7)	93.6% (92.6%, 94.5%)	34% (32.1%, 35.8%)
			High	45.6% (-31.6%, 87.9%)	-14.4 (-15, -13.7)	481.2 (449.7, 512.7)	83.3% (81.9%, 84.8%)	40.2% (38.3%, 42.1%)

			Very High	39.9% (-38.4%, 81.3%)	-20.1 (-20.7, -19.4)	665 (629.3, 700.6)	67.9% (66.1%, 69.7%)	46.9% (45%, 48.9%)
		Yes	Very Low	56.1% (-150%, 100%)	-3.9 (-4.8, -3)	572.4 (510.1, 634.7)	96.6% (95.9%, 97.3%)	55.6% (53.7%, 57.6%)
			Low	57.3% (-40%, 91.3%)	-2.7 (-3.3, -2.1)	241.5 (221.2, 261.7)	95.4% (94.6%, 96.3%)	23.6% (21.9%, 25.3%)
			Medium	57.1% (-16.6%, 92%)	-2.9 (-3.5, -2.3)	256.3 (237.1, 275.5)	95.4% (94.5%, 96.2%)	25.9% (24.2%, 27.6%)
			High	55.2% (-11.7%, 91.1%)	-4.8 (-5.4, -4.2)	235.8 (219, 252.7)	94% (93.1%, 95%)	23.2% (21.5%, 24.8%)
			Very High	53.1% (-6.9%, 93.2%)	-6.9 (-7.5, -6.4)	241.6 (224.3, 258.8)	93% (92%, 94%)	21.8% (20.2%, 23.4%)
	Inferior	No	Very Low	55.5% (-133.3%, 100%)	-4.5 (-5.5, -3.6)	590.9 (530, 651.8)	96% (95.2%, 96.8%)	55.5% (53.5%, 57.4%)
			Low	56.4% (-16.7%, 92.5%)	-3.6 (-4.1, -3)	227.3 (210.3, 244.3)	95.1% (94.2%, 95.9%)	21.8% (20.1%, 23.4%)
			Medium	55.8% (-26.6%, 90%)	-4.2 (-4.8, -3.6)	252.2 (233, 271.5)	94.8% (93.9%, 95.7%)	24.8% (23.1%, 26.5%)
			High	52.6% (-19.3%, 87.5%)	-7.4 (-7.9, -6.8)	243.6 (226, 261.1)	91.8% (90.7%, 92.9%)	21% (19.4%, 22.6%)
			Very High	48.4% (-7%, 82.9%)	-11.6 (-12.1, -11.1)	304.1 (285.6, 322.7)	84.7% (83.3%, 86.1%)	21.5% (19.9%, 23.1%)
		Yes	Very Low	55.9% (-100%, 100%)	-4.1 (-5, -3.2)	571.9 (517.5, 626.4)	96.2% (95.5%, 96.9%)	54.6% (52.7%, 56.6%)
			Low	58.2% (-13.3%, 92.9%)	-1.8 (-2.4, -1.3)	208.4 (193.2, 223.6)	95.9% (95.1%, 96.7%)	19% (17.5%, 20.6%)
			Medium	57.6% (-20%, 91.2%)	-2.4 (-2.9, -1.8)	229.9 (213.1, 246.7)	95.2% (94.3%, 96%)	22.8% (21.1%, 24.4%)
			High	56.8% (-3.9%, 93.5%)	-3.2 (-3.7, -2.7)	173.2 (160.4, 186)	95.4% (94.6%, 96.3%)	12.9% (11.6%, 14.2%)
			Very High	54.6% (-24.9%, 88.9%)	-5.4 (-5.8, -4.9)	179.6 (166.2, 192.9)	93.4% (92.4%, 94.3%)	11.8% (10.5%, 13.1%)

Table O. Mean estimate, bias, mean squared error (MSE), coverage, and false negative rate for vaccine efficacy (VE) estimates against severe *Shigella* diarrhea from realistically sized trials for all 50 simulation scenarios when using symptom-based reporting and a crude recurrent outcome regression model. Hybrid immunity was either not possible (no) or possible such that the first post-vaccination infection conferred additional immunity (yes). Bias was calculated as the average VE estimate for a set of simulations minus the true VE (60%). MSE was calculated as the average of the square of the estimate minus the true parameter. Coverage represents the percent of 95% confidence intervals (CIs) that contained the true VE (60%), and the false negative rate indicates the percent of 95% CIs that incorrectly indicated a non-significant effect.

VE against infection	Strength of infection-acquired immunity	Hybrid immunity	Force of infection	Mean VE estimate (range)	Bias (95% CI)	MSE (95% CI)	Coverage (95% CI)	False negatives (95% CI)
0%	Superior	Yes	Very Low	51.2% (-133.3%, 100%)	-8.8 (-9.8, -7.8)	757.1 (677.2, 837.1)	94.8% (93.9%, 95.7%)	62.9% (61%, 64.8%)
			Low	52.7% (-41.7%, 94.4%)	-7.3 (-8, -6.7)	326.6 (298.3, 354.8)	93.8% (92.9%, 94.7%)	31.7% (29.9%, 33.5%)
			Medium	50.8% (-53.3%, 88.5%)	-9.2 (-9.9, -8.5)	394 (363.3, 424.6)	91.6% (90.5%, 92.6%)	39.6% (37.7%, 41.6%)
			High	46.8% (-41.1%, 90.5%)	-13.2 (-13.9, -12.6)	453.8 (423.6, 484.1)	86% (84.6%, 87.3%)	41.2% (39.3%, 43.1%)
			Very High	42.5% (-37.4%, 81.3%)	-17.5 (-18.2, -16.8)	587 (552.4, 621.6)	77.4% (75.8%, 79%)	45.4% (43.4%, 47.3%)
	Similar	No	Very Low	55.9% (-100%, 100%)	-4.1 (-5, -3.2)	547.4 (496.8, 598)	96.5% (95.8%, 97.2%)	55.6% (53.7%, 57.5%)
			Low	53.9% (-64.3%, 91.4%)	-6.1 (-6.7, -5.5)	283 (259.5, 306.5)	94.1% (93.2%, 95%)	28.2% (26.4%, 29.9%)
			Medium	52.9% (-30.7%, 91.7%)	-7.1 (-7.7, -6.4)	317.7 (293.5, 341.9)	93.8% (92.9%, 94.8%)	32.4% (30.5%, 34.2%)
			High	45.9% (-47.8%, 83.7%)	-14.1 (-14.7, -13.5)	422.7 (396.4, 449)	81.4% (79.9%, 82.9%)	34.2% (32.4%, 36.1%)
			Very High	39.6% (-21.6%, 72.4%)	-20.4 (-20.9, -19.8)	592.4 (564.6, 620.3)	56.6% (54.6%, 58.5%)	31.5% (29.7%, 33.3%)
		Yes	Very Low	57.4% (-60%, 100%)	-2.6 (-3.5, -1.7)	520.3 (475.5, 565.1)	97.4% (96.8%, 98%)	53.3% (51.3%, 55.2%)
			Low	58.8% (-11.8%, 94.4%)	-1.2 (-1.8, -0.7)	195.4 (181.9, 208.8)	96.6% (95.9%, 97.3%)	19.8% (18.3%, 21.4%)
			Medium	58.1% (-42.8%, 94.3%)	-1.9 (-2.5, -1.3)	243.4 (222.5, 264.4)	95.7% (94.9%, 96.5%)	23% (21.4%, 24.6%)
			High	56.5% (-17.4%, 93.3%)	-3.5 (-4.1, -2.9)	219.1 (202.3, 235.9)	94.9% (94%, 95.7%)	19.8% (18.2%, 21.4%)
			Very High	54.4% (-8.6%, 91.1%)	-5.6 (-6.2, -5.1)	234.2 (217.8, 250.6)	93.4% (92.4%, 94.4%)	21.2% (19.6%, 22.8%)

	Inferior	No	Very Low	56.5% (-120%, 100%)	-3.5 (-4.4, -2.6)	525 (474.2, 575.8)	97.2% (96.6%, 97.8%)	54.6% (52.6%, 56.5%)
			Low	56.2% (-19%, 89.7%)	-3.8 (-4.4, -3.2)	236.1 (218.1, 254)	95.4% (94.6%, 96.2%)	22.6% (21%, 24.2%)
			Medium	55.4% (-17.6%, 94.6%)	-4.6 (-5.2, -4)	259.4 (240, 278.9)	94.1% (93.2%, 95%)	26.7% (24.9%, 28.4%)
			High	51.8% (-29.4%, 84.6%)	-8.2 (-8.8, -7.7)	272.2 (252.2, 292.2)	90.3% (89.1%, 91.4%)	22.8% (21.1%, 24.4%)
			Very High	48.7% (-11.5%, 83.7%)	-11.3 (-11.8, -10.8)	290.3 (272.3, 308.4)	86% (84.6%, 87.3%)	20.2% (18.7%, 21.8%)
		Yes	Very Low	57.1% (-120%, 100%)	-2.9 (-3.8, -2)	520.7 (470.9, 570.4)	97% (96.4%, 97.7%)	53.3% (51.3%, 55.2%)
			Low	58.4% (-15.8%, 91.2%)	-1.6 (-2.2, -1)	210.8 (194.7, 226.9)	95.7% (94.9%, 96.5%)	18.8% (17.3%, 20.4%)
			Medium	58% (-20%, 95%)	-2 (-2.6, -1.4)	223.6 (206.9, 240.3)	95.1% (94.2%, 95.9%)	21% (19.4%, 22.6%)
			High	56.8% (-3.9%, 88.2%)	-3.2 (-3.7, -2.7)	188.4 (174.7, 202.1)	94.4% (93.5%, 95.3%)	15% (13.6%, 16.4%)
			Very High	55.7% (-14.2%, 90.9%)	-4.3 (-4.8, -3.8)	165 (152.6, 177.4)	93.9% (92.9%, 94.8%)	9.8% (8.6%, 10.9%)
20%	Superior	Yes	Very Low	55.2% (-233.4%, 100%)	-4.8 (-5.8, -3.8)	625.6 (538.2, 713)	96.2% (95.5%, 97%)	57.1% (55.1%, 59%)
			Low	56.1% (-46.1%, 89.5%)	-3.9 (-4.5, -3.3)	246.6 (225.7, 267.5)	95.7% (94.9%, 96.5%)	24.5% (22.8%, 26.2%)
			Medium	54.6% (-41.2%, 96.2%)	-5.4 (-6, -4.7)	299.3 (276.2, 322.5)	94.6% (93.7%, 95.5%)	31.5% (29.7%, 33.3%)
			High	50.6% (-46.6%, 88.4%)	-9.4 (-10.1, -8.8)	342.6 (315.8, 369.4)	90.8% (89.6%, 91.9%)	33.1% (31.2%, 34.9%)
			Very High	46% (-45%, 84.9%)	-14 (-14.6, -13.3)	468 (436, 499.9)	83.6% (82.1%, 85.1%)	38.6% (36.7%, 40.5%)
	Similar	No	Very Low	54.4% (-200%, 100%)	-5.6 (-6.6, -4.6)	657.9 (575.6, 740.3)	95.8% (95.1%, 96.6%)	57.5% (55.6%, 59.5%)
			Low	54% (-41.6%, 90.5%)	-6 (-6.6, -5.4)	284.5 (260.8, 308.2)	94.4% (93.5%, 95.3%)	29.2% (27.4%, 30.9%)
			Medium	52.6% (-41.6%, 93.1%)	-7.4 (-8.1, -6.8)	334.8 (308.8, 360.8)	93.6% (92.6%, 94.5%)	34% (32.1%, 35.8%)
			High	45.6% (-31.6%, 87.9%)	-14.4 (-15, -13.7)	481.4 (449.9, 513)	83.3% (81.9%, 84.8%)	40.2% (38.3%, 42.1%)

			Very High	39.9% (-38.4%, 81.3%)	-20.1 (-20.7, -19.4)	665.4 (629.8, 701.1)	67.9% (66%, 69.7%)	46.9% (45%, 48.9%)
		Yes	Very Low	56.1% (-150%, 100%)	-3.9 (-4.8, -3)	572.4 (510.1, 634.7)	96.6% (95.9%, 97.3%)	55.6% (53.7%, 57.6%)
			Low	57.3% (-40%, 91.3%)	-2.7 (-3.3, -2.1)	241.5 (221.3, 261.7)	95.4% (94.6%, 96.3%)	23.5% (21.8%, 25.1%)
			Medium	57.1% (-16.6%, 92%)	-2.9 (-3.6, -2.3)	256.3 (237.1, 275.5)	95.4% (94.5%, 96.2%)	25.9% (24.2%, 27.6%)
			High	55.2% (-11.8%, 91.1%)	-4.8 (-5.4, -4.2)	236 (219.2, 252.8)	94.1% (93.2%, 95%)	23.2% (21.5%, 24.8%)
			Very High	53.1% (-6.9%, 93.2%)	-6.9 (-7.5, -6.4)	241.9 (224.6, 259.2)	93% (92%, 94%)	21.8% (20.2%, 23.4%)
	Inferior	No	Very Low	55.5% (-133.3%, 100%)	-4.5 (-5.5, -3.6)	590.9 (530, 651.8)	96% (95.2%, 96.8%)	55.5% (53.5%, 57.4%)
			Low	56.4% (-16.7%, 92.5%)	-3.6 (-4.1, -3)	227.3 (210.3, 244.4)	95.1% (94.2%, 95.9%)	21.7% (20.1%, 23.3%)
			Medium	55.8% (-26.6%, 90%)	-4.2 (-4.8, -3.6)	252.3 (233.1, 271.6)	94.8% (93.9%, 95.7%)	24.8% (23.1%, 26.5%)
			High	52.6% (-19.3%, 87.5%)	-7.4 (-7.9, -6.9)	243.9 (226.3, 261.4)	91.8% (90.7%, 92.9%)	21% (19.4%, 22.6%)
			Very High	48.4% (-7.1%, 82.9%)	-11.6 (-12.1, -11.1)	305 (286.4, 323.5)	84.7% (83.3%, 86.1%)	21.5% (19.9%, 23.1%)
		Yes	Very Low	55.9% (-100%, 100%)	-4.1 (-5, -3.2)	572 (517.5, 626.4)	96.2% (95.5%, 96.9%)	54.6% (52.7%, 56.6%)
			Low	58.2% (-13.3%, 92.9%)	-1.8 (-2.4, -1.3)	208.4 (193.2, 223.6)	96% (95.2%, 96.7%)	19% (17.5%, 20.6%)
			Medium	57.6% (-20%, 91.2%)	-2.4 (-2.9, -1.8)	230 (213.1, 246.8)	95.2% (94.3%, 96%)	22.8% (21.1%, 24.4%)
			High	56.8% (-4%, 93.5%)	-3.2 (-3.7, -2.7)	173.4 (160.6, 186.2)	95.4% (94.6%, 96.3%)	13% (11.7%, 14.3%)
			Very High	54.6% (-25%, 88.9%)	-5.4 (-5.9, -4.9)	179.9 (166.5, 193.3)	93.4% (92.4%, 94.4%)	11.8% (10.6%, 13.1%)

Table P. Mean estimate, bias, mean squared error (MSE), coverage, and false negative rate for vaccine efficacy (VE) estimates against any *Shigella* diarrhea from highly powered trials for all 50 simulation scenarios when using active surveillance for infection and a single outcome regression model. Infection-acquired immunity was either inferior or similar to vaccine-acquired immunity. Hybrid immunity was either not possible (no) or possible such that the first post-vaccination infection conferred additional immunity (yes). Bias was calculated as the average VE estimate for a set of simulations minus the true VE (60%). MSE was calculated as the average of the square of the estimate minus the true parameter. Coverage represents the percent of 95% confidence intervals (CIs) that contained the true VE (60%), and the false negative rate indicates the percent of 95% CIs that incorrectly indicated a non-significant effect.

VE against infection	Strength of infection-acquired immunity	Hybrid immunity	Force of infection	Mean VE estimate (range)	Bias (95% CI)	MSE (95% CI)	Coverage (95% CI)	False negatives (95% CI)
0%	Superior	Yes	Very Low	34.3% (12.9%, 52.8%)	-5.7 (-6.3, -5.1)	81.3 (71.6, 90.9)	87% (84.1%, 89.9%)	1.2% (0.2%, 2.2%)
			Low	35.2% (18.2%, 44.6%)	-4.8 (-5.2, -4.5)	39.5 (35, 44)	75.6% (71.8%, 79.4%)	0% (0%, 0%)
			Medium	34.9% (17.1%, 43.6%)	-5.1 (-5.4, -4.8)	39.6 (35.4, 43.8)	71.2% (67.2%, 75.2%)	0% (0%, 0%)
			High	35.1% (27.1%, 43.1%)	-4.9 (-5.2, -4.7)	31.1 (28.6, 33.6)	55.4% (51%, 59.8%)	0% (0%, 0%)
			Very High	35% (26.8%, 41.6%)	-5 (-5.2, -4.8)	31.9 (29.5, 34.4)	47.4% (43%, 51.8%)	0% (0%, 0%)
	Similar	No	Very Low	39.6% (14%, 56.3%)	-0.4 (-1, 0.1)	42 (36.1, 47.8)	95% (93.1%, 96.9%)	0.4% (-0.2%, 1%)
			Low	39.6% (23.3%, 49.1%)	-0.4 (-0.7, -0.1)	14.4 (12.3, 16.4)	95.4% (93.6%, 97.2%)	0% (0%, 0%)
			Medium	39.8% (29.2%, 50.1%)	-0.2 (-0.5, 0.2)	13.2 (11.6, 14.7)	94.4% (92.4%, 96.4%)	0% (0%, 0%)
			High	39.9% (28.2%, 46.9%)	-0.1 (-0.3, 0.2)	6.9 (5.9, 7.9)	95.6% (93.8%, 97.4%)	0% (0%, 0%)
			Very High	40% (33.6%, 47.4%)	0 (-0.2, 0.3)	6 (5.3, 6.7)	95.4% (93.6%, 97.2%)	0% (0%, 0%)
		Yes	Very Low	39.6% (15.2%, 57.1%)	-0.4 (-1, 0.2)	42.4 (36.7, 48.1)	95.8% (94%, 97.6%)	0.4% (-0.2%, 1%)
			Low	39.6% (27.3%, 48.8%)	-0.4 (-0.7, -0.1)	13.8 (12, 15.6)	96.4% (94.8%, 98%)	0% (0%, 0%)
			Medium	39.7% (29.2%, 48.8%)	-0.3 (-0.6, 0)	12.5 (11.1, 14)	95.4% (93.6%, 97.2%)	0% (0%, 0%)
			High	39.9% (30.3%, 47.1%)	-0.1 (-0.3, 0.2)	7.5 (6.5, 8.4)	95% (93.1%, 96.9%)	0% (0%, 0%)
			Very High	40% (33.3%, 46.1%)	0 (-0.3, 0.2)	5.6 (4.9, 6.3)	95% (93.1%, 96.9%)	0% (0%, 0%)

	Inferior	No	Very Low	39.5% (13.8%, 57.4%)	-0.5 (-1.1, 0.1)	43.4 (37.3, 49.5)	94.8% (92.9%, 96.7%)	0.2% (-0.2%, 0.6%)
			Low	39.8% (26%, 51.4%)	-0.2 (-0.6, 0.1)	15.3 (13.3, 17.3)	94.6% (92.6%, 96.6%)	0% (0%, 0%)
			Medium	39.8% (29.6%, 49.5%)	-0.2 (-0.5, 0.1)	12.4 (11, 13.8)	95.4% (93.6%, 97.2%)	0% (0%, 0%)
			High	39.8% (30.6%, 49.3%)	-0.2 (-0.5, 0)	7 (6.1, 7.9)	95.8% (94%, 97.6%)	0% (0%, 0%)
			Very High	39.9% (33.1%, 45.9%)	-0.1 (-0.3, 0.1)	5.9 (5.2, 6.6)	94.8% (92.9%, 96.7%)	0% (0%, 0%)
		Yes	Very Low	39.5% (13.8%, 59.1%)	-0.5 (-1, 0.1)	43.3 (37.1, 49.6)	94.2% (92.2%, 96.2%)	0.2% (-0.2%, 0.6%)
			Low	39.8% (28.5%, 51.3%)	-0.2 (-0.6, 0.1)	15.1 (13.2, 17)	95% (93.1%, 96.9%)	0% (0%, 0%)
			Medium	39.7% (29.4%, 49.2%)	-0.3 (-0.6, 0)	12.6 (11.1, 14.1)	94% (91.9%, 96.1%)	0% (0%, 0%)
			High	39.8% (30.1%, 47.4%)	-0.2 (-0.4, 0.1)	6.8 (5.9, 7.7)	95.8% (94%, 97.6%)	0% (0%, 0%)
			Very High	39.9% (33%, 46.8%)	-0.1 (-0.3, 0.1)	5.9 (5.2, 6.6)	95.2% (93.3%, 97.1%)	0% (0%, 0%)
20%	Superior	Yes	Very Low	40% (15.5%, 58.2%)	0 (-0.5, 0.6)	43.7 (38, 49.4)	94.4% (92.4%, 96.4%)	0.2% (-0.2%, 0.6%)
			Low	40% (28.5%, 52.3%)	0 (-0.4, 0.3)	16.5 (14.4, 18.5)	93.6% (91.5%, 95.7%)	0% (0%, 0%)
			Medium	39.9% (30.3%, 50.9%)	-0.1 (-0.4, 0.2)	11.6 (10.2, 13)	95.8% (94%, 97.6%)	0% (0%, 0%)
			High	39.9% (31.6%, 47.3%)	-0.1 (-0.3, 0.1)	6.8 (5.9, 7.7)	94% (91.9%, 96.1%)	0% (0%, 0%)
			Very High	40% (32.8%, 46.5%)	0 (-0.2, 0.3)	5.9 (5.2, 6.6)	94% (91.9%, 96.1%)	0% (0%, 0%)
	Similar	No	Very Low	39.9% (13.2%, 57.3%)	-0.1 (-0.6, 0.5)	43.3 (37.3, 49.3)	95.6% (93.8%, 97.4%)	0.6% (-0.1%, 1.3%)
			Low	39.8% (26.9%, 52.1%)	-0.2 (-0.6, 0.1)	15.4 (13.5, 17.3)	94.8% (92.9%, 96.7%)	0% (0%, 0%)
			Medium	39.7% (27.4%, 48.9%)	-0.3 (-0.6, 0)	12.3 (10.8, 13.7)	95.6% (93.8%, 97.4%)	0% (0%, 0%)
			High	39.9% (31.2%, 47.4%)	-0.1 (-0.4, 0.1)	6.9 (6.1, 7.8)	94.4% (92.4%, 96.4%)	0% (0%, 0%)

			Very High	39.9% (31.8%, 45.9%)	-0.1 (-0.3, 0.1)	4.9 (4.3, 5.6)	95.4% (93.6%, 97.2%)	0% (0%, 0%)
		Yes	Very Low	40.1% (15.6%, 57.4%)	0.1 (-0.5, 0.6)	40.9 (35.7, 46.1)	96.2% (94.5%, 97.9%)	0.2% (-0.2%, 0.6%)
			Low	40% (28.4%, 50.1%)	0 (-0.3, 0.3)	13.8 (12.2, 15.5)	94.8% (92.9%, 96.7%)	0% (0%, 0%)
			Medium	39.9% (28.8%, 50.3%)	-0.1 (-0.4, 0.2)	11.9 (10.5, 13.4)	94.2% (92.2%, 96.2%)	0% (0%, 0%)
			High	39.9% (31.8%, 46.2%)	-0.1 (-0.4, 0.1)	6.2 (5.5, 6.9)	96.6% (95%, 98.2%)	0% (0%, 0%)
			Very High	39.8% (33.9%, 46.7%)	-0.2 (-0.4, 0)	5.3 (4.7, 6)	94.8% (92.9%, 96.7%)	0% (0%, 0%)
	Inferior	No	Very Low	39.5% (12%, 57.6%)	-0.5 (-1.1, 0.1)	49.5 (42.7, 56.3)	93.6% (91.5%, 95.7%)	0.6% (-0.1%, 1.3%)
			Low	39.7% (28.1%, 50.4%)	-0.3 (-0.6, 0.1)	14.5 (12.8, 16.1)	95.8% (94%, 97.6%)	0% (0%, 0%)
			Medium	39.7% (28.8%, 49.1%)	-0.3 (-0.6, 0)	13.5 (11.8, 15.1)	92.8% (90.5%, 95.1%)	0% (0%, 0%)
			High	40% (30.8%, 46.5%)	0 (-0.3, 0.2)	6.6 (5.8, 7.5)	95.6% (93.8%, 97.4%)	0% (0%, 0%)
			Very High	39.8% (30.8%, 47.2%)	-0.2 (-0.4, 0.1)	5.9 (5.1, 6.7)	94% (91.9%, 96.1%)	0% (0%, 0%)
		Yes	Very Low	39.5% (10.3%, 57.6%)	-0.5 (-1.1, 0.1)	49.1 (42.2, 55.9)	93.4% (91.2%, 95.6%)	0.6% (-0.1%, 1.3%)
			Low	39.8% (27.2%, 51.3%)	-0.2 (-0.6, 0.1)	14.5 (12.8, 16.3)	95.4% (93.6%, 97.2%)	0% (0%, 0%)
			Medium	39.7% (29.5%, 49.7%)	-0.3 (-0.6, 0)	13.2 (11.6, 14.8)	93% (90.8%, 95.2%)	0% (0%, 0%)
			High	40% (31.9%, 48.2%)	0 (-0.2, 0.2)	6.7 (5.8, 7.5)	94.2% (92.2%, 96.2%)	0% (0%, 0%)
			Very High	39.9% (31.3%, 47.4%)	-0.1 (-0.4, 0.1)	5.5 (4.7, 6.3)	94.4% (92.4%, 96.4%)	0% (0%, 0%)

Table Q. Mean estimate, bias, mean squared error (MSE), coverage, and false negative rate for vaccine efficacy (VE) estimates against any *Shigella* diarrhea from highly powered trials for all 50 simulation scenarios when using symptom-based reporting and a single outcome regression model. Infection-acquired immunity was either inferior or similar to vaccine-acquired immunity. Hybrid immunity was either not possible (no) or possible such that the first post-vaccination infection conferred additional immunity (yes). Bias was calculated as the average VE estimate for a set of simulations minus the true VE (60%). MSE was calculated as the average of the square of the estimate minus the true parameter. Coverage represents the percent of 95% confidence intervals (CIs) that contained the true VE (60%), and the false negative rate indicates the percent of 95% CIs that incorrectly indicated a non-significant effect.

VE against infection	Strength of infection-acquired immunity	Hybrid immunity	Force of infection	Mean VE estimate (range)	Bias (95% CI)	MSE (95% CI)	Coverage (95% CI)	False negatives (95% CI)
0%	Superior	Yes	Very Low	33.6% (11.1%, 53.4%)	-6.4 (-7, -5.8)	89.2 (78.9, 99.5)	83.4% (80.1%, 86.7%)	1% (0.1%, 1.9%)
			Low	33.4% (17.4%, 43%)	-6.6 (-7, -6.3)	59.3 (53.9, 64.7)	61% (56.7%, 65.3%)	0% (0%, 0%)
			Medium	32.3% (16.3%, 40.5%)	-7.7 (-8, -7.4)	71.9 (66.5, 77.4)	37.8% (33.5%, 42.1%)	0% (0%, 0%)
			High	29.2% (21.8%, 35.7%)	-10.8 (-11, -10.5)	121.9 (117.1, 126.8)	0.6% (-0.1%, 1.3%)	0% (0%, 0%)
			Very High	26.1% (18.9%, 31.8%)	-13.9 (-14.1, -13.7)	199.1 (193.6, 204.6)	0% (0%, 0%)	0% (0%, 0%)
	Similar	No	Very Low	38.5% (12.2%, 57.3%)	-1.5 (-2, -0.9)	44.6 (38.1, 51.1)	94.6% (92.6%, 96.6%)	0.4% (-0.2%, 1%)
			Low	36.3% (20.8%, 48.3%)	-3.7 (-4, -3.4)	27 (23.6, 30.4)	85.2% (82.1%, 88.3%)	0% (0%, 0%)
			Medium	35.7% (26.2%, 45.5%)	-4.3 (-4.6, -3.9)	31.5 (28.5, 34.5)	72% (68.1%, 75.9%)	0% (0%, 0%)
			High	30.7% (21.8%, 38%)	-9.3 (-9.5, -9.1)	93.1 (88.8, 97.4)	2.8% (1.4%, 4.2%)	0% (0%, 0%)
			Very High	26.5% (19.9%, 32.9%)	-13.5 (-13.7, -13.3)	188.2 (182.6, 193.9)	0% (0%, 0%)	0% (0%, 0%)
		Yes	Very Low	39.8% (16.5%, 58%)	-0.2 (-0.7, 0.4)	40.5 (35.1, 45.9)	95% (93.1%, 96.9%)	0.2% (-0.2%, 0.6%)
			Low	40% (27.6%, 49.4%)	0 (-0.3, 0.3)	12.4 (10.7, 14)	96% (94.3%, 97.7%)	0% (0%, 0%)
			Medium	40.2% (31.4%, 49.9%)	0.2 (-0.1, 0.5)	11.3 (10, 12.6)	93.2% (91%, 95.4%)	0% (0%, 0%)
			High	39.5% (31%, 46.5%)	-0.5 (-0.7, -0.3)	5.8 (5, 6.6)	94.2% (92.2%, 96.2%)	0% (0%, 0%)
			Very High	37.3% (30.6%, 43.6%)	-2.7 (-2.9, -2.5)	11.5 (10.4, 12.6)	71.8% (67.9%, 75.7%)	0% (0%, 0%)

	Inferior	No	Very Low	39.3% (13.6%, 57.8%)	-0.7 (-1.3, -0.1)	42.1 (36.1, 48.1)	95% (93.1%, 96.9%)	0.2% (-0.2%, 0.6%)
			Low	39% (29.1%, 49.2%)	-1 (-1.4, -0.7)	14.1 (12.3, 15.8)	93.8% (91.7%, 95.9%)	0% (0%, 0%)
			Medium	38.8% (29.7%, 49%)	-1.2 (-1.5, -0.9)	11.9 (10.5, 13.3)	94.6% (92.6%, 96.6%)	0% (0%, 0%)
			High	37.2% (30.3%, 44%)	-2.8 (-3, -2.6)	13.4 (12.1, 14.7)	72.6% (68.7%, 76.5%)	0% (0%, 0%)
			Very High	35.6% (30.2%, 41.3%)	-4.4 (-4.5, -4.2)	22.3 (20.9, 23.8)	34.6% (30.4%, 38.8%)	0% (0%, 0%)
		Yes	Very Low	39.6% (13.6%, 58.7%)	-0.4 (-1, 0.2)	41.7 (35.7, 47.8)	95% (93.1%, 96.9%)	0.2% (-0.2%, 0.6%)
			Low	39.9% (29%, 50.7%)	-0.1 (-0.4, 0.2)	12.5 (11, 14.1)	95.6% (93.8%, 97.4%)	0% (0%, 0%)
			Medium	39.9% (30%, 49.4%)	-0.1 (-0.4, 0.1)	10.3 (9.1, 11.5)	95.4% (93.6%, 97.2%)	0% (0%, 0%)
			High	39.6% (32%, 45.1%)	-0.4 (-0.6, -0.2)	4.9 (4.3, 5.5)	95.2% (93.3%, 97.1%)	0% (0%, 0%)
			Very High	38.7% (33.7%, 44.1%)	-1.3 (-1.4, -1.1)	4.9 (4.4, 5.4)	88.8% (86%, 91.6%)	0% (0%, 0%)
20%	Superior	Yes	Very Low	39.5% (15.3%, 57.6%)	-0.5 (-1.1, 0.1)	44.2 (38.3, 50.1)	95% (93.1%, 96.9%)	0.2% (-0.2%, 0.6%)
			Low	38.3% (27.2%, 51.6%)	-1.7 (-2.1, -1.4)	19.6 (17.2, 22.1)	91.4% (88.9%, 93.9%)	0% (0%, 0%)
			Medium	37.7% (28.5%, 49.6%)	-2.3 (-2.6, -2)	16.4 (14.5, 18.3)	89.6% (86.9%, 92.3%)	0% (0%, 0%)
			High	34.4% (25.9%, 42.3%)	-5.6 (-5.8, -5.4)	37.9 (35.3, 40.6)	35.8% (31.6%, 40%)	0% (0%, 0%)
			Very High	31.1% (24.3%, 36.7%)	-8.9 (-9.1, -8.7)	83.3 (79.8, 86.9)	0.2% (-0.2%, 0.6%)	0% (0%, 0%)
	Similar	No	Very Low	38.9% (12.6%, 56.4%)	-1.1 (-1.7, -0.5)	44.4 (38.2, 50.5)	95% (93.1%, 96.9%)	0.6% (-0.1%, 1.3%)
			Low	36.5% (23.6%, 47.6%)	-3.5 (-3.8, -3.2)	27.6 (24.5, 30.6)	84.4% (81.2%, 87.6%)	0% (0%, 0%)
			Medium	35.5% (23.3%, 43.4%)	-4.5 (-4.8, -4.2)	31.8 (28.6, 35)	72.6% (68.7%, 76.5%)	0% (0%, 0%)
			High	30.7% (22.5%, 37.2%)	-9.3 (-9.5, -9.1)	92.6 (88.3, 96.9)	2.8% (1.4%, 4.2%)	0% (0%, 0%)

			Very High	26.4% (20.8%, 32.6%)	-13.6 (-13.8, -13.4)	189.7 (184.7, 194.8)	0% (0%, 0%)	0% (0%, 0%)
		Yes	Very Low	39.9% (15.3%, 56.8%)	-0.1 (-0.7, 0.4)	40.6 (35.5, 45.8)	95.8% (94%, 97.6%)	0.2% (-0.2%, 0.6%)
			Low	39.5% (27.7%, 49%)	-0.5 (-0.8, -0.2)	13.8 (12, 15.5)	94.4% (92.4%, 96.4%)	0% (0%, 0%)
			Medium	39.2% (28.6%, 49.7%)	-0.8 (-1.1, -0.5)	11.8 (10.3, 13.4)	93.8% (91.7%, 95.9%)	0% (0%, 0%)
			High	37.7% (30.3%, 43.5%)	-2.3 (-2.5, -2.1)	10.4 (9.3, 11.6)	84.4% (81.2%, 87.6%)	0% (0%, 0%)
			Very High	35.3% (28.4%, 41.8%)	-4.7 (-4.9, -4.5)	26.6 (24.7, 28.5)	36.8% (32.6%, 41%)	0% (0%, 0%)
	Inferior	No	Very Low	39.2% (14%, 56%)	-0.8 (-1.4, -0.2)	47.8 (41.5, 54.1)	93.8% (91.7%, 95.9%)	0.6% (-0.1%, 1.3%)
			Low	39% (26.9%, 48.6%)	-1 (-1.3, -0.7)	13.8 (12.2, 15.5)	94.8% (92.9%, 96.7%)	0% (0%, 0%)
			Medium	38.6% (27.3%, 47.8%)	-1.4 (-1.7, -1.1)	13.6 (12, 15.3)	91.8% (89.4%, 94.2%)	0% (0%, 0%)
			High	37.3% (28.2%, 43.8%)	-2.7 (-2.9, -2.5)	12.4 (11, 13.8)	76.2% (72.5%, 79.9%)	0% (0%, 0%)
			Very High	35.7% (29.2%, 42.9%)	-4.3 (-4.5, -4.1)	22.9 (21.3, 24.6)	36.2% (32%, 40.4%)	0% (0%, 0%)
		Yes	Very Low	39.4% (13.5%, 56.2%)	-0.6 (-1.2, 0)	47 (40.8, 53.3)	94.2% (92.2%, 96.2%)	0.4% (-0.2%, 1%)
			Low	39.8% (28%, 49.7%)	-0.2 (-0.5, 0.1)	12.7 (11.2, 14.1)	94.4% (92.4%, 96.4%)	0% (0%, 0%)
			Medium	39.5% (28.6%, 48.9%)	-0.5 (-0.8, -0.2)	11.5 (10.1, 12.9)	94.4% (92.4%, 96.4%)	0% (0%, 0%)
			High	39.2% (32.1%, 45%)	-0.8 (-1, -0.6)	5.4 (4.7, 6.1)	94.4% (92.4%, 96.4%)	0% (0%, 0%)
			Very High	38.2% (31.8%, 44.7%)	-1.8 (-2, -1.6)	7.1 (6.2, 7.9)	80% (76.5%, 83.5%)	0% (0%, 0%)

Table R. Mean estimate, bias, mean squared error (MSE), coverage, and false negative rate for vaccine efficacy (VE) estimates against any *Shigella* diarrhea from highly powered trials for all 50 simulation scenarios when using active surveillance for infection and a stratified recurrent outcome regression model. Hybrid immunity was either not possible (no) or possible such that the first post-vaccination infection conferred additional immunity (yes). Bias was calculated as the average VE estimate for a set of simulations minus the true VE (60%). MSE was calculated as the average of the square of the estimate minus the true parameter. Coverage represents the percent of 95% confidence intervals (CIs) that contained the true VE (60%), and the false negative rate indicates the percent of 95% CIs that incorrectly indicated a non-significant effect.

VE against infection	Strength of infection-acquired immunity	Hybrid immunity	Force of infection	Mean VE estimate (range)	Bias (95% CI)	MSE (95% CI)	Coverage (95% CI)	False negatives (95% CI)
0%	Superior	Yes	Very Low	33.1% (11.5%, 52.1%)	-6.9 (-7.5, -6.3)	96.5 (85.6, 107.4)	80.6% (77.1%, 84.1%)	1.6% (0.5%, 2.7%)
			Low	31.7% (15.2%, 41.7%)	-8.3 (-8.6, -7.9)	84.6 (78, 91.3)	41.2% (36.9%, 45.5%)	0% (0%, 0%)
			Medium	30.4% (15.2%, 38.8%)	-9.6 (-9.9, -9.3)	105.8 (99.2, 112.5)	19.8% (16.3%, 23.3%)	0% (0%, 0%)
			High	25.2% (17.1%, 31.7%)	-14.8 (-15, -14.5)	224.2 (217.6, 230.8)	0% (0%, 0%)	0% (0%, 0%)
			Very High	20.8% (14.3%, 27.1%)	-19.2 (-19.4, -19)	374 (366.6, 381.4)	0% (0%, 0%)	0% (0%, 0%)
	Similar	No	Very Low	38% (10.9%, 57.7%)	-2 (-2.6, -1.4)	47.9 (40.8, 54.9)	94.2% (92.2%, 96.2%)	0.4% (-0.2%, 1%)
			Low	34.6% (17%, 47.2%)	-5.4 (-5.7, -5)	42.6 (38.2, 47.1)	71.6% (67.6%, 75.6%)	0% (0%, 0%)
			Medium	33.6% (24.3%, 44.1%)	-6.4 (-6.7, -6.1)	54.4 (50.2, 58.7)	49% (44.6%, 53.4%)	0% (0%, 0%)
			High	26.3% (16.8%, 33.2%)	-13.7 (-13.9, -13.5)	193 (187, 198.9)	0% (0%, 0%)	0% (0%, 0%)
			Very High	20.5% (14.3%, 26.5%)	-19.5 (-19.7, -19.3)	383.8 (376.1, 391.4)	0% (0%, 0%)	0% (0%, 0%)
		Yes	Very Low	39.6% (17.8%, 58.8%)	-0.4 (-1, 0.1)	41 (35.5, 46.5)	94.6% (92.6%, 96.6%)	0.2% (-0.2%, 0.6%)
			Low	39.2% (27.2%, 48.9%)	-0.8 (-1.1, -0.5)	13.1 (11.3, 14.8)	96.4% (94.8%, 98%)	0% (0%, 0%)
			Medium	39.1% (30.2%, 48.1%)	-0.9 (-1.2, -0.6)	12.2 (10.8, 13.6)	93.4% (91.2%, 95.6%)	0% (0%, 0%)
			High	36.7% (28.9%, 44.3%)	-3.3 (-3.5, -3.1)	16.2 (14.6, 17.7)	68.6% (64.5%, 72.7%)	0% (0%, 0%)
			Very High	32.9% (26.5%, 38.5%)	-7.1 (-7.3, -6.9)	53.9 (51.5, 56.4)	3.6% (2%, 5.2%)	0% (0%, 0%)

	Inferior	No	Very Low	39.2% (13.2%, 57.7%)	-0.8 (-1.4, -0.3)	43 (36.8, 49.1)	93.8% (91.7%, 95.9%)	0.4% (-0.2%, 1%)
			Low	38.5% (26.9%, 47.8%)	-1.5 (-1.9, -1.2)	15.1 (13.2, 16.9)	93.2% (91%, 95.4%)	0% (0%, 0%)
			Medium	38.1% (29.4%, 47.7%)	-1.9 (-2.2, -1.6)	13.8 (12.2, 15.4)	91.8% (89.4%, 94.2%)	0% (0%, 0%)
			High	35.4% (28.2%, 41.3%)	-4.6 (-4.7, -4.4)	25.1 (23.3, 26.9)	39.8% (35.5%, 44.1%)	0% (0%, 0%)
			Very High	32.6% (27%, 38.1%)	-7.4 (-7.6, -7.3)	57.7 (55.5, 59.9)	0.8% (0%, 1.6%)	0% (0%, 0%)
		Yes	Very Low	39.6% (13.2%, 58.9%)	-0.4 (-1, 0.1)	42.2 (36, 48.4)	94.6% (92.6%, 96.6%)	0.4% (-0.2%, 1%)
			Low	39.6% (27.3%, 50%)	-0.4 (-0.7, -0.1)	12.3 (10.8, 13.9)	95% (93.1%, 96.9%)	0% (0%, 0%)
			Medium	39.5% (30.4%, 48%)	-0.5 (-0.8, -0.2)	10 (8.9, 11.2)	95.2% (93.3%, 97.1%)	0% (0%, 0%)
			High	38.4% (31.3%, 43.6%)	-1.6 (-1.7, -1.4)	6.3 (5.6, 7.1)	90% (87.4%, 92.6%)	0% (0%, 0%)
			Very High	36.6% (32%, 41.5%)	-3.4 (-3.5, -3.2)	14.1 (13.1, 15.1)	44.8% (40.4%, 49.2%)	0% (0%, 0%)
20%	Superior	Yes	Very Low	39.4% (18%, 57.5%)	-0.6 (-1.1, 0)	43.8 (38.1, 49.6)	94.4% (92.4%, 96.4%)	0.2% (-0.2%, 0.6%)
			Low	38.1% (27.2%, 50.7%)	-1.9 (-2.3, -1.6)	19.8 (17.4, 22.3)	89.6% (86.9%, 92.3%)	0% (0%, 0%)
			Medium	37.5% (29.3%, 48.9%)	-2.5 (-2.8, -2.2)	17 (15.1, 18.8)	88.8% (86%, 91.6%)	0% (0%, 0%)
			High	33.7% (25.2%, 40.7%)	-6.3 (-6.5, -6.1)	46.1 (43.2, 49)	24% (20.3%, 27.7%)	0% (0%, 0%)
			Very High	29.9% (22.9%, 36.4%)	-10.1 (-10.3, -9.9)	106.1 (102.3, 109.9)	0.2% (-0.2%, 0.6%)	0% (0%, 0%)
	Similar	No	Very Low	38.6% (14.5%, 56.4%)	-1.4 (-2, -0.8)	45.5 (39.3, 51.7)	94.8% (92.9%, 96.7%)	0.6% (-0.1%, 1.3%)
			Low	35.4% (23.5%, 46.1%)	-4.6 (-4.9, -4.2)	36 (32.4, 39.6)	74.8% (71%, 78.6%)	0% (0%, 0%)
			Medium	34.2% (22%, 42.3%)	-5.8 (-6.1, -5.5)	45.3 (41.5, 49.2)	59% (54.7%, 63.3%)	0% (0%, 0%)
			High	27.6% (18.6%, 35.7%)	-12.4 (-12.6, -12.2)	159.8 (154.1, 165.5)	0% (0%, 0%)	0% (0%, 0%)

			Very High	21.9% (16.4%, 27.3%)	-18.1 (-18.3, -18)	333.3 (326.8, 339.9)	0% (0%, 0%)	0% (0%, 0%)
		Yes	Very Low	40% (18.1%, 57.2%)	0 (-0.6, 0.5)	40.2 (35.2, 45.1)	96% (94.3%, 97.7%)	0.2% (-0.2%, 0.6%)
			Low	39.7% (26.5%, 48.6%)	-0.3 (-0.6, 0)	13.2 (11.5, 14.9)	94.8% (92.9%, 96.7%)	0% (0%, 0%)
			Medium	39.4% (28.1%, 48.9%)	-0.6 (-0.9, -0.3)	10.9 (9.5, 12.4)	93.4% (91.2%, 95.6%)	0% (0%, 0%)
			High	37.7% (32.1%, 42.9%)	-2.3 (-2.5, -2.1)	9.7 (8.7, 10.7)	84.4% (81.2%, 87.6%)	0% (0%, 0%)
			Very High	34.8% (29.4%, 40.9%)	-5.2 (-5.4, -5)	31.3 (29.4, 33.2)	20.8% (17.2%, 24.4%)	0% (0%, 0%)
	Inferior	No	Very Low	39.2% (15.4%, 56%)	-0.8 (-1.4, -0.2)	47.1 (41, 53.3)	93.4% (91.2%, 95.6%)	0.6% (-0.1%, 1.3%)
			Low	38.7% (26.6%, 48.3%)	-1.3 (-1.6, -1)	13.7 (12, 15.5)	94.4% (92.4%, 96.4%)	0% (0%, 0%)
			Medium	38.2% (27.8%, 47.3%)	-1.8 (-2.1, -1.5)	13.9 (12.3, 15.6)	91.2% (88.7%, 93.7%)	0% (0%, 0%)
			High	36.3% (27.4%, 42.4%)	-3.7 (-3.9, -3.5)	18.2 (16.5, 19.8)	57% (52.7%, 61.3%)	0% (0%, 0%)
			Very High	33.8% (27.6%, 39.7%)	-6.2 (-6.3, -6)	41.4 (39.3, 43.5)	3.6% (2%, 5.2%)	0% (0%, 0%)
		Yes	Very Low	39.5% (14.9%, 56%)	-0.5 (-1.1, 0.1)	46 (40, 52)	94.6% (92.6%, 96.6%)	0.4% (-0.2%, 1%)
			Low	39.8% (28.2%, 48%)	-0.2 (-0.5, 0.1)	11.7 (10.4, 13.1)	96.2% (94.5%, 97.9%)	0% (0%, 0%)
			Medium	39.5% (29.5%, 48.3%)	-0.5 (-0.8, -0.2)	10.4 (9.2, 11.7)	94.8% (92.9%, 96.7%)	0% (0%, 0%)
			High	39% (32.6%, 44.8%)	-1 (-1.2, -0.9)	5.3 (4.6, 6)	90.6% (88%, 93.2%)	0% (0%, 0%)
			Very High	37.2% (31.4%, 42.2%)	-2.8 (-2.9, -2.6)	10.7 (9.7, 11.7)	60.2% (55.9%, 64.5%)	0% (0%, 0%)

Table S. Mean estimate, bias, mean squared error (MSE), coverage, and false negative rate for vaccine efficacy (VE) estimates against any *Shigella* diarrhea from highly powered trials for all 50 simulation scenarios when using symptom-based reporting and a stratified recurrent outcome regression model. Hybrid immunity was either not possible (no) or possible such that the first post-vaccination infection conferred additional immunity (yes). Bias was calculated as the average VE estimate for a set of simulations minus the true VE (60%). MSE was calculated as the average of the square of the estimate minus the true parameter. Coverage represents the percent of 95% confidence intervals (CIs) that contained the true VE (60%), and the false negative rate indicates the percent of 95% CIs that incorrectly indicated a non-significant effect.

VE against infection	Strength of infection-acquired immunity	Hybrid immunity	Force of infection	Mean VE estimate (range)	Bias (95% CI)	MSE (95% CI)	Coverage (95% CI)	False negatives (95% CI)
0%	Superior	Yes	Very Low	33.3% (11.7%, 52.6%)	-6.7 (-7.3, -6.1)	93.2 (82.6, 103.9)	81.2% (77.8%, 84.6%)	1.2% (0.2%, 2.2%)
			Low	32.4% (15.9%, 42.5%)	-7.6 (-7.9, -7.2)	73.6 (67.4, 79.8)	48.8% (44.4%, 53.2%)	0% (0%, 0%)
			Medium	31.2% (16.1%, 39.5%)	-8.8 (-9.1, -8.5)	90.2 (84.1, 96.3)	26.8% (22.9%, 30.7%)	0% (0%, 0%)
			High	26.6% (18.5%, 33.1%)	-13.4 (-13.6, -13.1)	184.4 (178.5, 190.3)	0% (0%, 0%)	0% (0%, 0%)
			Very High	22.4% (15.9%, 28.4%)	-17.6 (-17.8, -17.4)	313.1 (306.5, 319.8)	0% (0%, 0%)	0% (0%, 0%)
	Similar	No	Very Low	38.1% (11.3%, 57.5%)	-1.9 (-2.4, -1.3)	47.4 (40.5, 54.3)	94.4% (92.4%, 96.4%)	0.4% (-0.2%, 1%)
			Low	35.1% (17.8%, 48%)	-4.9 (-5.2, -4.6)	38 (33.9, 42.2)	75.6% (71.8%, 79.4%)	0% (0%, 0%)
			Medium	34.2% (24.6%, 44.4%)	-5.8 (-6.2, -5.5)	47.7 (43.7, 51.6)	56.4% (52.1%, 60.7%)	0% (0%, 0%)
			High	27.2% (17.6%, 34%)	-12.8 (-13.1, -12.6)	170.9 (165.3, 176.5)	0% (0%, 0%)	0% (0%, 0%)
			Very High	21.4% (15.1%, 27.2%)	-18.6 (-18.8, -18.5)	352.8 (345.4, 360.2)	0% (0%, 0%)	0% (0%, 0%)
		Yes	Very Low	39.8% (18%, 58.7%)	-0.2 (-0.8, 0.4)	40.8 (35.4, 46.2)	94.6% (92.6%, 96.6%)	0.2% (-0.2%, 0.6%)
			Low	39.9% (28.2%, 49.3%)	-0.1 (-0.4, 0.2)	12.3 (10.7, 13.9)	96.2% (94.5%, 97.9%)	0% (0%, 0%)
			Medium	39.9% (31.1%, 48.6%)	-0.1 (-0.4, 0.2)	11.1 (9.8, 12.3)	94% (91.9%, 96.1%)	0% (0%, 0%)
			High	38.3% (30.2%, 45.7%)	-1.7 (-1.9, -1.5)	8.3 (7.2, 9.3)	87% (84.1%, 89.9%)	0% (0%, 0%)
			Very High	34.8% (28.2%, 40.4%)	-5.2 (-5.3, -5)	30.4 (28.6, 32.2)	21.2% (17.6%, 24.8%)	0% (0%, 0%)

	Inferior	No	Very Low	39.2% (13.4%, 57.5%)	-0.8 (-1.4, -0.2)	43 (36.8, 49.2)	94.4% (92.4%, 96.4%)	0.4% (-0.2%, 1%)
			Low	38.6% (27.5%, 48.3%)	-1.4 (-1.7, -1.1)	14.7 (12.8, 16.5)	93.6% (91.5%, 95.7%)	0% (0%, 0%)
			Medium	38.3% (29.5%, 47.7%)	-1.7 (-2, -1.4)	13.1 (11.6, 14.6)	92.4% (90.1%, 94.7%)	0% (0%, 0%)
			High	35.9% (28.5%, 41.9%)	-4.1 (-4.3, -4)	21.5 (19.8, 23.1)	48% (43.6%, 52.4%)	0% (0%, 0%)
			Very High	33.2% (27.3%, 38.7%)	-6.8 (-6.9, -6.6)	48.9 (46.8, 51)	1.4% (0.4%, 2.4%)	0% (0%, 0%)
		Yes	Very Low	39.6% (13.4%, 58.9%)	-0.4 (-1, 0.2)	42.3 (36.1, 48.5)	94.4% (92.4%, 96.4%)	0.4% (-0.2%, 1%)
			Low	39.8% (27.9%, 50.4%)	-0.2 (-0.5, 0.1)	12.2 (10.7, 13.7)	95.2% (93.3%, 97.1%)	0% (0%, 0%)
			Medium	39.7% (30.8%, 48.2%)	-0.3 (-0.6, 0)	9.8 (8.7, 10.8)	96% (94.3%, 97.7%)	0% (0%, 0%)
			High	38.9% (31.8%, 44%)	-1.1 (-1.2, -0.9)	5 (4.4, 5.7)	93.6% (91.5%, 95.7%)	0% (0%, 0%)
			Very High	37.3% (32.4%, 42.1%)	-2.7 (-2.8, -2.5)	9.8 (8.9, 10.6)	63.8% (59.6%, 68%)	0% (0%, 0%)
20%	Superior	Yes	Very Low	39.3% (17.7%, 57.3%)	-0.7 (-1.2, -0.1)	44.2 (38.4, 50)	94.2% (92.2%, 96.2%)	0.2% (-0.2%, 0.6%)
			Low	37.7% (26.7%, 50.6%)	-2.3 (-2.6, -1.9)	21.5 (18.9, 24.1)	89% (86.3%, 91.7%)	0% (0%, 0%)
			Medium	37% (28.2%, 48.6%)	-3 (-3.3, -2.7)	19.8 (17.7, 21.9)	85.8% (82.7%, 88.9%)	0% (0%, 0%)
			High	32.5% (23.8%, 39.6%)	-7.5 (-7.7, -7.2)	62.1 (58.6, 65.5)	10.4% (7.7%, 13.1%)	0% (0%, 0%)
			Very High	28.2% (20.3%, 34.4%)	-11.8 (-12, -11.6)	144.4 (139.9, 148.9)	0% (0%, 0%)	0% (0%, 0%)
	Similar	No	Very Low	38.5% (14.6%, 56.5%)	-1.5 (-2, -0.9)	45.8 (39.6, 52)	94.8% (92.9%, 96.7%)	0.6% (-0.1%, 1.3%)
			Low	35.2% (23.2%, 45.8%)	-4.8 (-5.1, -4.4)	37.7 (33.9, 41.5)	73.6% (69.7%, 77.5%)	0% (0%, 0%)
			Medium	33.9% (21.7%, 42.1%)	-6.1 (-6.4, -5.8)	48.1 (44.1, 52.1)	55.8% (51.4%, 60.2%)	0% (0%, 0%)
			High	27.1% (18%, 35.6%)	-12.9 (-13.1, -12.7)	172.5 (166.6, 178.5)	0% (0%, 0%)	0% (0%, 0%)

			Very High	21.2% (15.6%, 26.6%)	-18.8 (-18.9, -18.6)	356.2 (349.4, 363)	0% (0%, 0%)	0% (0%, 0%)
		Yes	Very Low	39.9% (17.7%, 56.9%)	-0.1 (-0.7, 0.4)	40.4 (35.4, 45.3)	95.8% (94%, 97.6%)	0.2% (-0.2%, 0.6%)
			Low	39.4% (25.9%, 48.4%)	-0.6 (-0.9, -0.3)	13.6 (11.8, 15.4)	95% (93.1%, 96.9%)	0% (0%, 0%)
			Medium	39% (27.3%, 48.7%)	-1 (-1.3, -0.7)	11.8 (10.2, 13.4)	93% (90.8%, 95.2%)	0% (0%, 0%)
			High	36.7% (30.6%, 42.1%)	-3.3 (-3.4, -3.1)	15.1 (13.7, 16.4)	70% (66%, 74%)	0% (0%, 0%)
			Very High	33.4% (27.8%, 39.5%)	-6.6 (-6.8, -6.4)	48.5 (46, 50.9)	6.6% (4.4%, 8.8%)	0% (0%, 0%)
	Inferior	No	Very Low	39.2% (15.9%, 55.9%)	-0.8 (-1.4, -0.2)	47.2 (41.1, 53.4)	93.4% (91.2%, 95.6%)	0.6% (-0.1%, 1.3%)
			Low	38.7% (26.8%, 48.4%)	-1.3 (-1.6, -1)	13.9 (12.2, 15.7)	94% (91.9%, 96.1%)	0% (0%, 0%)
			Medium	38.2% (28.2%, 47.6%)	-1.8 (-2.1, -1.5)	14.2 (12.5, 15.9)	90.4% (87.8%, 93%)	0% (0%, 0%)
			High	36.2% (27.4%, 42.3%)	-3.8 (-4, -3.6)	19.5 (17.7, 21.2)	53.2% (48.8%, 57.6%)	0% (0%, 0%)
			Very High	33.6% (27.3%, 39.8%)	-6.4 (-6.5, -6.2)	43.9 (41.7, 46)	3.6% (2%, 5.2%)	0% (0%, 0%)
		Yes	Very Low	39.5% (15%, 55.9%)	-0.5 (-1.1, 0.1)	46.1 (40.1, 52)	94.4% (92.4%, 96.4%)	0.4% (-0.2%, 1%)
			Low	39.7% (28.3%, 48.4%)	-0.3 (-0.6, 0)	11.9 (10.5, 13.2)	95.8% (94%, 97.6%)	0% (0%, 0%)
			Medium	39.4% (29.1%, 48.4%)	-0.6 (-0.9, -0.3)	10.6 (9.3, 11.8)	94% (91.9%, 96.1%)	0% (0%, 0%)
			High	38.8% (32.5%, 44.4%)	-1.2 (-1.4, -1.1)	5.9 (5.2, 6.6)	89.6% (86.9%, 92.3%)	0% (0%, 0%)
			Very High	37% (31.2%, 42.2%)	-3 (-3.1, -2.8)	12 (11, 13.1)	57% (52.7%, 61.3%)	0% (0%, 0%)

Table T. Mean estimate, bias, mean squared error (MSE), coverage, and false negative rate for vaccine efficacy (VE) estimates against any *Shigella* diarrhea from highly powered trials for all 50 simulation scenarios when using active surveillance for infection and a crude recurrent outcome regression model. Hybrid immunity was either not possible (no) or possible such that the first post-vaccination infection conferred additional immunity (yes). Bias was calculated as the average VE estimate for a set of simulations minus the true VE (60%). MSE was calculated as the average of the square of the estimate minus the true parameter. Coverage represents the percent of 95% confidence intervals (CIs) that contained the true VE (60%), and the false negative rate indicates the percent of 95% CIs that incorrectly indicated a non-significant effect.

VE against infection	Strength of infection-acquired immunity	Hybrid immunity	Force of infection	Mean VE estimate (range)	Bias (95% CI)	MSE (95% CI)	Coverage (95% CI)	False negatives (95% CI)
0%	Superior	Yes	Very Low	33.1% (11.7%, 52.1%)	-6.9 (-7.5, -6.3)	95.7 (85, 106.5)	80% (76.5%, 83.5%)	1.2% (0.2%, 2.2%)
			Low	31.7% (15.7%, 41.6%)	-8.3 (-8.6, -7.9)	83.9 (77.4, 90.4)	39.6% (35.3%, 43.9%)	0% (0%, 0%)
			Medium	30.4% (15.6%, 38.6%)	-9.6 (-9.9, -9.3)	104.9 (98.5, 111.3)	17.6% (14.3%, 20.9%)	0% (0%, 0%)
			High	25.2% (17.5%, 31.4%)	-14.8 (-15, -14.6)	224.3 (218, 230.5)	0% (0%, 0%)	0% (0%, 0%)
			Very High	20.8% (14.4%, 26.6%)	-19.2 (-19.4, -19)	373.4 (366.4, 380.3)	0% (0%, 0%)	0% (0%, 0%)
	Similar	No	Very Low	38% (11.3%, 57.7%)	-2 (-2.6, -1.4)	47.5 (40.5, 54.5)	94% (91.9%, 96.1%)	0.4% (-0.2%, 1%)
			Low	34.6% (17.3%, 47.2%)	-5.4 (-5.7, -5)	42.3 (37.9, 46.6)	71.2% (67.2%, 75.2%)	0% (0%, 0%)
			Medium	33.6% (24.3%, 43.7%)	-6.4 (-6.7, -6.1)	54.1 (49.8, 58.3)	48% (43.6%, 52.4%)	0% (0%, 0%)
			High	26.3% (17.1%, 33%)	-13.7 (-13.9, -13.5)	192.4 (186.6, 198.2)	0% (0%, 0%)	0% (0%, 0%)
			Very High	20.5% (14.5%, 26.3%)	-19.5 (-19.7, -19.3)	384.1 (376.6, 391.6)	0% (0%, 0%)	0% (0%, 0%)
		Yes	Very Low	39.6% (18%, 58.8%)	-0.4 (-1, 0.1)	40.4 (35, 45.9)	94.8% (92.9%, 96.7%)	0.2% (-0.2%, 0.6%)
			Low	39.2% (27.5%, 48.7%)	-0.8 (-1.1, -0.5)	12.5 (10.9, 14.2)	96.2% (94.5%, 97.9%)	0% (0%, 0%)
			Medium	39.1% (30.4%, 47.9%)	-0.9 (-1.2, -0.6)	11.6 (10.2, 12.9)	93.2% (91%, 95.4%)	0% (0%, 0%)
			High	36.7% (28.9%, 43.9%)	-3.3 (-3.5, -3.1)	15.7 (14.2, 17.1)	67.6% (63.5%, 71.7%)	0% (0%, 0%)
			Very High	32.9% (26.8%, 38.2%)	-7.1 (-7.2, -6.9)	53.5 (51.2, 55.8)	2.6% (1.2%, 4%)	0% (0%, 0%)

	Inferior	No	Very Low	39.2% (13.4%, 57.7%)	-0.8 (-1.4, -0.3)	42.9 (36.7, 49)	94.2% (92.2%, 96.2%)	0.4% (-0.2%, 1%)
			Low	38.5% (27%, 47.9%)	-1.5 (-1.9, -1.2)	15 (13.1, 16.9)	93.2% (91%, 95.4%)	0% (0%, 0%)
			Medium	38.1% (29.4%, 47.6%)	-1.9 (-2.2, -1.6)	13.7 (12.1, 15.3)	91.8% (89.4%, 94.2%)	0% (0%, 0%)
			High	35.4% (28.2%, 41.3%)	-4.6 (-4.7, -4.4)	25 (23.2, 26.7)	38.8% (34.5%, 43.1%)	0% (0%, 0%)
			Very High	32.6% (27%, 37.9%)	-7.4 (-7.6, -7.3)	57.6 (55.4, 59.8)	0.8% (0%, 1.6%)	0% (0%, 0%)
		Yes	Very Low	39.6% (13.4%, 58.8%)	-0.4 (-1, 0.1)	42.1 (35.9, 48.2)	94.4% (92.4%, 96.4%)	0.4% (-0.2%, 1%)
			Low	39.6% (27.4%, 50%)	-0.4 (-0.7, -0.1)	12.2 (10.7, 13.7)	95% (93.1%, 96.9%)	0% (0%, 0%)
			Medium	39.5% (30.4%, 47.9%)	-0.5 (-0.8, -0.2)	9.9 (8.8, 11)	95.4% (93.6%, 97.2%)	0% (0%, 0%)
			High	38.4% (31.3%, 43.6%)	-1.6 (-1.7, -1.4)	6.2 (5.4, 7)	90.2% (87.6%, 92.8%)	0% (0%, 0%)
			Very High	36.6% (32.1%, 41.4%)	-3.4 (-3.5, -3.2)	14 (13, 15)	43% (38.7%, 47.3%)	0% (0%, 0%)
20%	Superior	Yes	Very Low	39.1% (17.7%, 57%)	-0.9 (-1.5, -0.3)	44.2 (38.4, 50.1)	94% (91.9%, 96.1%)	0.2% (-0.2%, 0.6%)
			Low	37% (26.1%, 49.8%)	-3 (-3.4, -2.7)	25.1 (22.1, 28)	83.8% (80.6%, 87%)	0% (0%, 0%)
			Medium	36.1% (27.5%, 47.6%)	-3.9 (-4.2, -3.6)	25.6 (23.2, 28.1)	76.6% (72.9%, 80.3%)	0% (0%, 0%)
			High	30.9% (22.4%, 37.9%)	-9.1 (-9.3, -8.8)	87.9 (84, 91.9)	2.4% (1.1%, 3.7%)	0% (0%, 0%)
			Very High	26.3% (18.7%, 32.6%)	-13.7 (-13.9, -13.5)	191.1 (186.1, 196)	0% (0%, 0%)	0% (0%, 0%)
	Similar	No	Very Low	38.4% (14.6%, 55.9%)	-1.6 (-2.2, -1)	46 (39.7, 52.2)	95.2% (93.3%, 97.1%)	0.6% (-0.1%, 1.3%)
			Low	34.8% (22.9%, 45.4%)	-5.2 (-5.5, -4.9)	41.7 (37.7, 45.6)	69.2% (65.2%, 73.2%)	0% (0%, 0%)
			Medium	33.4% (21.6%, 41.6%)	-6.6 (-6.9, -6.3)	54.4 (50.2, 58.6)	49% (44.6%, 53.4%)	0% (0%, 0%)
			High	26.3% (17.5%, 34.5%)	-13.7 (-13.9, -13.4)	193 (186.8, 199.2)	0% (0%, 0%)	0% (0%, 0%)

			Very High	20.4%<br>(15%, 25.6%)	-19.6<br>(-19.7, -19.4)	386.5<br>(379.6, 393.4)	0%<br>(0%, 0%)	0%<br>(0%, 0%)
		Yes	Very Low	39.7%<br>(17.7%, 56.4%)	-0.3<br>(-0.9, 0.2)	40.1<br>(35.2, 45.1)	96%<br>(94.3%, 97.7%)	0.2%<br>(-0.2%, 0.6%)
			Low	38.7%<br>(25.6%, 47.6%)	-1.3<br>(-1.6, -0.9)	14.6<br>(12.7, 16.5)	93%<br>(90.8%, 95.2%)	0%<br>(0%, 0%)
			Medium	38.2%<br>(26.7%, 47.6%)	-1.8<br>(-2.1, -1.5)	13.8<br>(11.9, 15.7)	89%<br>(86.3%, 91.7%)	0%<br>(0%, 0%)
			High	35.2%<br>(28.8%, 40.1%)	-4.8<br>(-5, -4.6)	27.1<br>(25.3, 28.9)	38.8%<br>(34.5%, 43.1%)	0%<br>(0%, 0%)
			Very High	31.5%<br>(26.2%, 37.4%)	-8.5<br>(-8.7, -8.3)	76.8<br>(73.7, 79.8)	1%<br>(0.1%, 1.9%)	0%<br>(0%, 0%)
	Inferior	No	Very Low	39.1%<br>(15.9%, 55.8%)	-0.9<br>(-1.5, -0.3)	47.2<br>(41, 53.4)	93.6%<br>(91.5%, 95.7%)	0.6%<br>(-0.1%, 1.3%)
			Low	38.5%<br>(26.8%, 48.2%)	-1.5<br>(-1.8, -1.2)	14.2<br>(12.4, 16)	93.8%<br>(91.7%, 95.9%)	0%<br>(0%, 0%)
			Medium	38%<br>(27.8%, 47%)	-2<br>(-2.3, -1.7)	14.7<br>(13, 16.4)	90%<br>(87.4%, 92.6%)	0%<br>(0%, 0%)
			High	35.8%<br>(27.2%, 41.8%)	-4.2<br>(-4.3, -4)	21.9<br>(20.1, 23.7)	47.4%<br>(43%, 51.8%)	0%<br>(0%, 0%)
			Very High	33.2%<br>(27.2%, 39%)	-6.8<br>(-7, -6.6)	49.6<br>(47.3, 51.8)	1.6%<br>(0.5%, 2.7%)	0%<br>(0%, 0%)
		Yes	Very Low	39.4%<br>(15%, 56.1%)	-0.6<br>(-1.2, 0)	46<br>(40.1, 52)	94.6%<br>(92.6%, 96.6%)	0.4%<br>(-0.2%, 1%)
			Low	39.5%<br>(28.3%, 48.1%)	-0.5<br>(-0.8, -0.2)	11.9<br>(10.5, 13.3)	95.6%<br>(93.8%, 97.4%)	0%<br>(0%, 0%)
			Medium	39.2%<br>(28.8%, 48%)	-0.8<br>(-1.1, -0.5)	10.7<br>(9.4, 12)	94.2%<br>(92.2%, 96.2%)	0%<br>(0%, 0%)
			High	38.3%<br>(32.2%, 44%)	-1.7<br>(-1.9, -1.5)	7<br>(6.2, 7.9)	85%<br>(81.9%, 88.1%)	0%<br>(0%, 0%)
			Very High	36.4%<br>(30.7%, 41.2%)	-3.6<br>(-3.8, -3.5)	16.1<br>(14.9, 17.2)	39%<br>(34.7%, 43.3%)	0%<br>(0%, 0%)

Table U. Mean estimate, bias, mean squared error (MSE), coverage, and false negative rate for vaccine efficacy (VE) estimates against any *Shigella* diarrhea from highly powered trials for all 50 simulation scenarios when using symptom-based reporting and a crude recurrent outcome regression model. Hybrid immunity was either not possible (no) or possible such that the first post-vaccination infection conferred additional immunity (yes). Bias was calculated as the average VE estimate for a set of simulations minus the true VE (60%). MSE was calculated as the average of the square of the estimate minus the true parameter. Coverage represents the percent of 95% confidence intervals (CIs) that contained the true VE (60%), and the false negative rate indicates the percent of 95% CIs that incorrectly indicated a non-significant effect.

VE against infection	Strength of infection-acquired immunity	Hybrid immunity	Force of infection	Mean VE estimate (range)	Bias (95% CI)	MSE (95% CI)	Coverage (95% CI)	False negatives (95% CI)
0%	Superior	Yes	Very Low	33.1% (11.7%, 52.1%)	-6.9 (-7.5, -6.3)	95.7 (84.9, 106.5)	80% (76.5%, 83.5%)	1.2% (0.2%, 2.2%)
			Low	31.7% (15.7%, 41.6%)	-8.3 (-8.6, -7.9)	83.7 (77.3, 90.2)	39.8% (35.5%, 44.1%)	0% (0%, 0%)
			Medium	30.4% (15.6%, 38.7%)	-9.6 (-9.9, -9.3)	104.7 (98.3, 111.1)	17.6% (14.3%, 20.9%)	0% (0%, 0%)
			High	25.2% (17.5%, 31.4%)	-14.8 (-15, -14.6)	223.7 (217.5, 229.9)	0% (0%, 0%)	0% (0%, 0%)
			Very High	20.8% (14.4%, 26.6%)	-19.2 (-19.4, -19)	372.4 (365.4, 379.3)	0% (0%, 0%)	0% (0%, 0%)
	Similar	No	Very Low	38% (11.3%, 57.7%)	-2 (-2.6, -1.4)	47.5 (40.5, 54.5)	94% (91.9%, 96.1%)	0.4% (-0.2%, 1%)
			Low	34.7% (17.3%, 47.2%)	-5.3 (-5.7, -5)	42.2 (37.8, 46.5)	71.2% (67.2%, 75.2%)	0% (0%, 0%)
			Medium	33.6% (24.3%, 43.8%)	-6.4 (-6.7, -6.1)	53.9 (49.7, 58.1)	48% (43.6%, 52.4%)	0% (0%, 0%)
			High	26.4% (17.1%, 33%)	-13.6 (-13.9, -13.4)	191.8 (186, 197.6)	0% (0%, 0%)	0% (0%, 0%)
			Very High	20.5% (14.5%, 26.4%)	-19.5 (-19.6, -19.3)	383.1 (375.6, 390.5)	0% (0%, 0%)	0% (0%, 0%)
		Yes	Very Low	39.6% (18%, 58.8%)	-0.4 (-1, 0.1)	40.4 (35, 45.9)	94.8% (92.9%, 96.7%)	0.2% (-0.2%, 0.6%)
			Low	39.2% (27.5%, 48.7%)	-0.8 (-1.1, -0.4)	12.5 (10.9, 14.2)	96.2% (94.5%, 97.9%)	0% (0%, 0%)
			Medium	39.1% (30.4%, 47.9%)	-0.9 (-1.2, -0.6)	11.5 (10.2, 12.9)	93.4% (91.2%, 95.6%)	0% (0%, 0%)
			High	36.8% (28.9%, 43.9%)	-3.2 (-3.4, -3)	15.5 (14, 17)	67.6% (63.5%, 71.7%)	0% (0%, 0%)
			Very High	33% (26.9%, 38.2%)	-7 (-7.2, -6.9)	53 (50.7, 55.3)	2.8% (1.4%, 4.2%)	0% (0%, 0%)

	Inferior	No	Very Low	39.2% (13.4%, 57.7%)	-0.8 (-1.4, -0.3)	42.9 (36.7, 49)	94.2% (92.2%, 96.2%)	0.4% (-0.2%, 1%)
			Low	38.5% (27%, 47.9%)	-1.5 (-1.9, -1.2)	15 (13.1, 16.8)	93.4% (91.2%, 95.6%)	0% (0%, 0%)
			Medium	38.1% (29.4%, 47.6%)	-1.9 (-2.2, -1.6)	13.7 (12.1, 15.3)	92% (89.6%, 94.4%)	0% (0%, 0%)
			High	35.5% (28.3%, 41.3%)	-4.5 (-4.7, -4.3)	24.7 (23, 26.5)	39.4% (35.1%, 43.7%)	0% (0%, 0%)
			Very High	32.6% (27.1%, 38%)	-7.4 (-7.5, -7.2)	57 (54.8, 59.2)	0.8% (0%, 1.6%)	0% (0%, 0%)
		Yes	Very Low	39.6% (13.4%, 58.8%)	-0.4 (-1, 0.1)	42.1 (35.9, 48.2)	94.4% (92.4%, 96.4%)	0.4% (-0.2%, 1%)
			Low	39.6% (27.4%, 50%)	-0.4 (-0.7, -0.1)	12.2 (10.7, 13.7)	95.2% (93.3%, 97.1%)	0% (0%, 0%)
			Medium	39.5% (30.4%, 47.9%)	-0.5 (-0.8, -0.2)	9.9 (8.8, 11)	95.4% (93.6%, 97.2%)	0% (0%, 0%)
			High	38.5% (31.4%, 43.6%)	-1.5 (-1.7, -1.4)	6.1 (5.4, 6.9)	90.2% (87.6%, 92.8%)	0% (0%, 0%)
			Very High	36.7% (32.1%, 41.5%)	-3.3 (-3.5, -3.2)	13.7 (12.7, 14.7)	44.6% (40.2%, 49%)	0% (0%, 0%)
20%	Superior	Yes	Very Low	39.1% (17.7%, 57%)	-0.9 (-1.5, -0.3)	44.2 (38.4, 50.1)	94% (91.9%, 96.1%)	0.2% (-0.2%, 0.6%)
			Low	37% (26.1%, 49.8%)	-3 (-3.4, -2.7)	25.1 (22.2, 28)	83.8% (80.6%, 87%)	0% (0%, 0%)
			Medium	36.1% (27.5%, 47.6%)	-3.9 (-4.2, -3.6)	25.7 (23.2, 28.2)	76.6% (72.9%, 80.3%)	0% (0%, 0%)
			High	30.9% (22.4%, 37.9%)	-9.1 (-9.3, -8.9)	88.2 (84.2, 92.2)	2.4% (1.1%, 3.7%)	0% (0%, 0%)
			Very High	26.3% (18.7%, 32.6%)	-13.7 (-13.9, -13.5)	191.7 (186.8, 196.7)	0% (0%, 0%)	0% (0%, 0%)
	Similar	No	Very Low	38.4% (14.6%, 55.9%)	-1.6 (-2.2, -1)	46 (39.7, 52.2)	95.2% (93.3%, 97.1%)	0.6% (-0.1%, 1.3%)
			Low	34.8% (22.9%, 45.4%)	-5.2 (-5.5, -4.9)	41.7 (37.8, 45.7)	69.2% (65.2%, 73.2%)	0% (0%, 0%)
			Medium	33.4% (21.6%, 41.5%)	-6.6 (-6.9, -6.3)	54.4 (50.2, 58.6)	49% (44.6%, 53.4%)	0% (0%, 0%)
			High	26.3% (17.5%, 34.5%)	-13.7 (-13.9, -13.4)	193.3 (187.1, 199.4)	0% (0%, 0%)	0% (0%, 0%)

			Very High	20.4% (15%, 25.6%)	-19.6 (-19.7, -19.4)	386.9 (380, 393.8)	0% (0%, 0%)	0% (0%, 0%)
		Yes	Very Low	39.7% (17.7%, 56.4%)	-0.3 (-0.9, 0.2)	40.1 (35.2, 45.1)	96% (94.3%, 97.7%)	0.2% (-0.2%, 0.6%)
			Low	38.7% (25.6%, 47.6%)	-1.3 (-1.6, -0.9)	14.6 (12.7, 16.5)	93% (90.8%, 95.2%)	0% (0%, 0%)
			Medium	38.2% (26.7%, 47.6%)	-1.8 (-2.1, -1.6)	13.8 (12, 15.7)	89% (86.3%, 91.7%)	0% (0%, 0%)
			High	35.2% (28.7%, 40.1%)	-4.8 (-5, -4.6)	27.2 (25.4, 29.1)	38.4% (34.1%, 42.7%)	0% (0%, 0%)
			Very High	31.5% (26.2%, 37.3%)	-8.5 (-8.7, -8.4)	77.1 (74, 80.1)	1% (0.1%, 1.9%)	0% (0%, 0%)
	Inferior	No	Very Low	39.1% (15.9%, 55.8%)	-0.9 (-1.5, -0.3)	47.2 (41, 53.4)	93.6% (91.5%, 95.7%)	0.6% (-0.1%, 1.3%)
			Low	38.5% (26.8%, 48.2%)	-1.5 (-1.8, -1.2)	14.2 (12.4, 16)	93.8% (91.7%, 95.9%)	0% (0%, 0%)
			Medium	38% (27.8%, 47%)	-2 (-2.3, -1.7)	14.7 (13, 16.4)	90% (87.4%, 92.6%)	0% (0%, 0%)
			High	35.8% (27.2%, 41.8%)	-4.2 (-4.4, -4)	22.1 (20.3, 23.9)	46.8% (42.4%, 51.2%)	0% (0%, 0%)
			Very High	33.2% (27.2%, 39%)	-6.8 (-7, -6.7)	50.1 (47.9, 52.4)	1.6% (0.5%, 2.7%)	0% (0%, 0%)
		Yes	Very Low	39.4% (14.9%, 56.1%)	-0.6 (-1.2, 0)	46 (40.1, 52)	94.6% (92.6%, 96.6%)	0.4% (-0.2%, 1%)
			Low	39.5% (28.3%, 48.1%)	-0.5 (-0.8, -0.2)	11.9 (10.5, 13.3)	95.6% (93.8%, 97.4%)	0% (0%, 0%)
			Medium	39.2% (28.8%, 48%)	-0.8 (-1.1, -0.5)	10.7 (9.5, 12)	94.2% (92.2%, 96.2%)	0% (0%, 0%)
			High	38.3% (32.1%, 44%)	-1.7 (-1.9, -1.5)	7.1 (6.2, 7.9)	84.8% (81.7%, 87.9%)	0% (0%, 0%)
			Very High	36.4% (30.7%, 41.2%)	-3.6 (-3.8, -3.5)	16.3 (15.1, 17.5)	38% (33.7%, 42.3%)	0% (0%, 0%)

Table V. Mean size of each trial arm, estimate, bias, mean squared error (MSE), coverage, and false negative rate for vaccine efficacy (VE) estimates against any *Shigella* diarrhea from realistically sized trials for all 50 simulation scenarios when using active surveillance for infection and a single outcome regression model. Hybrid immunity was either not possible (no) or possible such that the first post-vaccination infection conferred additional immunity (yes). Bias was calculated as the average VE estimate for a set of simulations minus the true VE (60%). MSE was calculated as the average of the square of the estimate minus the true parameter. Coverage represents the percent of 95% confidence intervals (CIs) that contained the true VE (60%), and the false negative rate indicates the percent of 95% CIs that incorrectly indicated a non-significant effect.

VE against infection	Strength of infection-acquired immunity	Hybrid immunity	Force of infection	Mean VE estimate (range)	Bias (95% CI)	MSE (95% CI)	Coverage (95% CI)	False negatives (95% CI)
0%	Superior	Yes	Very Low	34.3% (-11%, 57.8%)	-5.7 (-6.1, -5.4)	107 (100.5, 113.5)	91.6% (90.5%, 92.6%)	10.4% (9.2%, 11.6%)
			Low	34.8% (9.6%, 53.8%)	-5.2 (-5.5, -5)	67.2 (63.4, 70.9)	87.6% (86.3%, 88.9%)	0.5% (0.2%, 0.8%)
			Medium	34.5% (5.5%, 56.3%)	-5.5 (-5.8, -5.2)	75.7 (71.4, 80)	86.9% (85.6%, 88.2%)	1.4% (0.9%, 1.8%)
			High	34.5% (4.6%, 55.8%)	-5.5 (-5.7, -5.2)	77.6 (72.8, 82.3)	87% (85.7%, 88.3%)	1.6% (1.1%, 2.1%)
			Very High	34.7% (7.7%, 55.7%)	-5.3 (-5.5, -5)	76.6 (72.2, 81.1)	88% (86.8%, 89.3%)	2% (1.4%, 2.5%)
	Similar	No	Very Low	39.5% (3.7%, 62.9%)	-0.5 (-0.8, -0.2)	68.5 (64.5, 72.5)	95.7% (94.9%, 96.5%)	4% (3.3%, 4.8%)
			Low	39.5% (16%, 57.1%)	-0.5 (-0.8, -0.3)	37.3 (35.1, 39.4)	94.7% (93.8%, 95.6%)	0.1% (0%, 0.2%)
			Medium	39.8% (8.5%, 57.2%)	-0.2 (-0.5, 0)	38.5 (36.2, 40.8)	95% (94.1%, 95.8%)	0.2% (0%, 0.3%)
			High	39.5% (11%, 56.4%)	-0.5 (-0.8, -0.3)	37.1 (34.9, 39.4)	94.8% (93.9%, 95.7%)	0.1% (0%, 0.3%)
			Very High	39.7% (16.7%, 56.6%)	-0.3 (-0.6, -0.1)	30.2 (28.4, 31.9)	95% (94.2%, 95.9%)	0% (0%, 0%)
		Yes	Very Low	39.4% (5.4%, 62.7%)	-0.6 (-0.9, -0.2)	69.3 (65.2, 73.5)	96.2% (95.4%, 96.9%)	3.8% (3%, 4.5%)
			Low	39.5% (15.9%, 58.4%)	-0.5 (-0.8, -0.3)	36.1 (33.9, 38.3)	95% (94.2%, 95.9%)	0.2% (0%, 0.4%)
			Medium	39.6% (14.2%, 59.2%)	-0.4 (-0.6, -0.1)	39.8 (37.5, 42.1)	95.4% (94.5%, 96.2%)	0.1% (0%, 0.3%)
			High	39.4% (15.6%, 61.6%)	-0.6 (-0.8, -0.3)	43.3 (40.9, 45.8)	94.6% (93.8%, 95.5%)	0.1% (0%, 0.3%)
			Very High	39.7% (9%, 60.2%)	-0.3 (-0.6, -0.1)	42 (39.5, 44.6)	95.1% (94.2%, 95.9%)	0.3% (0.1%, 0.5%)

	Inferior	No	Very Low	39.5% (-3%, 63.2%)	-0.5 (-0.8, -0.1)	70.2 (65.8, 74.6)	95.3% (94.5%, 96.1%)	3.9% (3.1%, 4.6%)
			Low	39.5% (16.9%, 57.3%)	-0.5 (-0.7, -0.2)	36.3 (34.1, 38.5)	95.6% (94.8%, 96.4%)	0% (0%, 0.1%)
			Medium	39.7% (12%, 61.7%)	-0.3 (-0.6, -0.1)	40.8 (38.4, 43.2)	95.3% (94.5%, 96.1%)	0.2% (0%, 0.3%)
			High	39.5% (9.1%, 56.7%)	-0.5 (-0.7, -0.2)	41.9 (39.3, 44.5)	95.1% (94.2%, 95.9%)	0.4% (0.2%, 0.6%)
			Very High	39.6% (11.5%, 57.8%)	-0.4 (-0.6, -0.1)	41.6 (39.1, 44)	95.7% (94.9%, 96.5%)	0.2% (0%, 0.4%)
		Yes	Very Low	39.5% (-3%, 63.8%)	-0.5 (-0.8, -0.2)	69.5 (65.1, 73.9)	95.5% (94.7%, 96.3%)	3.8% (3%, 4.5%)
			Low	39.6% (14.4%, 57.5%)	-0.4 (-0.7, -0.2)	35.6 (33.5, 37.8)	95.7% (94.9%, 96.5%)	0.1% (0%, 0.2%)
			Medium	39.7% (15.7%, 57.4%)	-0.3 (-0.5, 0)	39.8 (37.5, 42.1)	95.6% (94.8%, 96.4%)	0.2% (0%, 0.4%)
			High	39.5% (10%, 58.1%)	-0.5 (-0.8, -0.3)	41.4 (38.8, 44)	95.3% (94.5%, 96.1%)	0.5% (0.2%, 0.8%)
			Very High	39.5% (14.1%, 60.8%)	-0.5 (-0.7, -0.2)	43.4 (40.9, 46)	95.4% (94.5%, 96.2%)	0.4% (0.1%, 0.6%)
20%	Superior	Yes	Very Low	39.3% (4.4%, 64.7%)	-0.7 (-1, -0.4)	70.9 (66.5, 75.3)	95.5% (94.7%, 96.3%)	3.4% (2.7%, 4.1%)
			Low	39.9% (16.6%, 56.7%)	-0.1 (-0.4, 0.1)	35.4 (33.3, 37.4)	95.2% (94.3%, 96%)	0.1% (0%, 0.2%)
			Medium	39.4% (15.9%, 60.5%)	-0.6 (-0.9, -0.4)	39.4 (37.2, 41.7)	95.2% (94.4%, 96.1%)	0.1% (0%, 0.3%)
			High	39.5% (5.3%, 58.2%)	-0.5 (-0.7, -0.2)	39.4 (36.9, 41.9)	94.8% (93.9%, 95.7%)	0.2% (0%, 0.4%)
			Very High	39.7% (16.4%, 57.1%)	-0.3 (-0.6, -0.1)	40.4 (38, 42.7)	94.7% (93.8%, 95.6%)	0.2% (0%, 0.4%)
	Similar	No	Very Low	39.5% (4.4%, 66.1%)	-0.5 (-0.9, -0.2)	72.9 (68.4, 77.4)	94.9% (94.1%, 95.8%)	4.3% (3.5%, 5.1%)
			Low	39.6% (17.5%, 57.4%)	-0.4 (-0.6, -0.2)	36.5 (34.4, 38.5)	94.8% (94%, 95.7%)	0% (0%, 0.1%)
			Medium	39.6% (10.1%, 58%)	-0.4 (-0.7, -0.2)	37.5 (35.4, 39.7)	95.8% (95%, 96.6%)	0% (0%, 0.1%)
			High	39.7% (15.5%, 56%)	-0.3 (-0.6, -0.1)	38.1 (35.9, 40.3)	95% (94.2%, 95.9%)	0.1% (0%, 0.2%)

			Very High	39.7% (13.9%, 57.9%)	-0.3 (-0.6, -0.1)	39.1 (36.9, 41.4)	95.4% (94.6%, 96.3%)	0.2% (0%, 0.3%)
		Yes	Very Low	39.4% (4%, 66.6%)	-0.6 (-0.9, -0.2)	70.9 (66.5, 75.4)	95% (94.1%, 95.9%)	3.6% (2.8%, 4.3%)
			Low	39.7% (14.7%, 55.7%)	-0.3 (-0.5, 0)	36 (34, 38)	95.1% (94.3%, 96%)	0% (0%, 0.1%)
			Medium	39.5% (13.4%, 57.3%)	-0.5 (-0.8, -0.3)	39.4 (37.1, 41.7)	95.2% (94.3%, 96%)	0.1% (0%, 0.2%)
			High	39.6% (13.4%, 59.2%)	-0.4 (-0.7, -0.2)	37.2 (35, 39.4)	95.8% (95%, 96.6%)	0% (0%, 0.1%)
			Very High	39.8% (13.6%, 57.9%)	-0.2 (-0.4, 0.1)	37.5 (35.4, 39.6)	95.8% (95%, 96.5%)	0.1% (0%, 0.2%)
	Inferior	No	Very Low	39.5% (1.5%, 67.1%)	-0.5 (-0.8, -0.1)	71.4 (66.9, 75.8)	95.1% (94.3%, 96%)	3.5% (2.8%, 4.2%)
			Low	39.7% (17.6%, 57.3%)	-0.3 (-0.5, 0)	35.6 (33.6, 37.7)	95.5% (94.7%, 96.3%)	0% (0%, 0%)
			Medium	39.6% (15.6%, 59.4%)	-0.4 (-0.6, -0.1)	36.6 (34.5, 38.7)	95.4% (94.6%, 96.2%)	0.1% (0%, 0.2%)
			High	39.7% (14.6%, 59.1%)	-0.3 (-0.6, -0.1)	39 (36.7, 41.2)	94.9% (94.1%, 95.8%)	0.2% (0%, 0.3%)
			Very High	39.5% (10.7%, 61.4%)	-0.5 (-0.7, -0.2)	40.3 (37.9, 42.6)	94.9% (94%, 95.7%)	0.1% (0%, 0.3%)
		Yes	Very Low	39.6% (1.8%, 67.1%)	-0.4 (-0.8, -0.1)	72.1 (67.5, 76.6)	95.1% (94.2%, 95.9%)	3.6% (2.9%, 4.4%)
			Low	39.7% (17.6%, 57.7%)	-0.3 (-0.5, -0.1)	35.6 (33.5, 37.6)	95.6% (94.8%, 96.4%)	0% (0%, 0%)
			Medium	39.5% (15.7%, 59.6%)	-0.5 (-0.7, -0.2)	38.4 (36.2, 40.6)	94.8% (94%, 95.7%)	0.1% (0%, 0.2%)
			High	39.7% (11.2%, 57.1%)	-0.3 (-0.6, -0.1)	37.6 (35.3, 39.9)	94.9% (94.1%, 95.8%)	0.1% (0%, 0.3%)
			Very High	39.7% (6.3%, 60.6%)	-0.3 (-0.5, 0)	40.9 (38.3, 43.4)	95.2% (94.4%, 96%)	0.1% (0%, 0.3%)

Table W. Mean estimate, bias, mean squared error (MSE), coverage, and false negative rate for vaccine efficacy (VE) estimates against any *Shigella* diarrhea from realistically sized trials for all 50 simulation scenarios when using symptom-based reporting and a single outcome regression model. Hybrid immunity was either not possible (no) or possible such that the first post-vaccination infection conferred additional immunity (yes). Bias was calculated as the average VE estimate for a set of simulations minus the true VE (60%). MSE was calculated as the average of the square of the estimate minus the true parameter. Coverage represents the percent of 95% confidence intervals (CIs) that contained the true VE (60%), and the false negative rate indicates the percent of 95% CIs that incorrectly indicated a non-significant effect.

VE against infection	Strength of infection-acquired immunity	Hybrid immunity	Force of infection	Mean VE estimate (range)	Bias (95% CI)	MSE (95% CI)	Coverage (95% CI)	False negatives (95% CI)
0%	Superior	Yes	Very Low	33.7% (-8.7%, 57.7%)	-6.3 (-6.7, -6)	113.2 (106.5, 119.8)	90.4% (89.3%, 91.6%)	10.7% (9.5%, 11.9%)
			Low	32.8% (10.1%, 50.7%)	-7.2 (-7.5, -7)	90.4 (86.1, 94.8)	78.1% (76.5%, 79.7%)	0.8% (0.4%, 1.1%)
			Medium	32.1% (7.5%, 53.5%)	-7.9 (-8.2, -7.7)	105.9 (100.8, 111)	75.8% (74.1%, 77.5%)	1.9% (1.3%, 2.4%)
			High	28.9% (1.1%, 50.5%)	-11.1 (-11.4, -10.9)	166.2 (159.6, 172.9)	53.8% (51.9%, 55.8%)	3.4% (2.7%, 4.2%)
			Very High	25.8% (-2.3%, 46.1%)	-14.2 (-14.5, -14)	242 (234.2, 249.7)	31.2% (29.4%, 33.1%)	5.4% (4.5%, 6.3%)
	Similar	No	Very Low	38.5% (3.5%, 62.9%)	-1.5 (-1.8, -1.2)	70.4 (66.2, 74.7)	95.6% (94.8%, 96.4%)	4.2% (3.4%, 4.9%)
			Low	36.2% (14.5%, 54.4%)	-3.8 (-4.1, -3.6)	50.1 (47.3, 53)	90.4% (89.2%, 91.6%)	0.2% (0%, 0.3%)
			Medium	35.7% (1.4%, 53.9%)	-4.3 (-4.6, -4.1)	55.7 (52.4, 59)	90.7% (89.6%, 91.9%)	0.2% (0%, 0.4%)
			High	30.2% (3.8%, 50.2%)	-9.8 (-10, -9.5)	127.3 (122.3, 132.2)	55.4% (53.5%, 57.4%)	0.6% (0.3%, 0.9%)
			Very High	26.2% (0.7%, 42%)	-13.8 (-14, -13.6)	215.4 (209.6, 221.2)	14% (12.6%, 15.3%)	0.6% (0.3%, 0.9%)
		Yes	Very Low	39.7% (6.9%, 62.5%)	-0.3 (-0.6, 0)	66.3 (62.4, 70.2)	96% (95.2%, 96.7%)	3% (2.4%, 3.7%)
			Low	39.9% (19%, 59.3%)	-0.1 (-0.3, 0.2)	31.7 (29.9, 33.6)	95.2% (94.3%, 96%)	0% (0%, 0%)
			Medium	40.1% (16.6%, 58.6%)	0.1 (-0.1, 0.4)	34.5 (32.6, 36.4)	95.2% (94.4%, 96.1%)	0% (0%, 0.1%)
			High	39.1% (18.4%, 56.3%)	-0.9 (-1.2, -0.7)	32.4 (30.5, 34.2)	95.6% (94.8%, 96.4%)	0% (0%, 0%)
			Very High	37.2% (14%, 55.5%)	-2.8 (-3, -2.6)	37.3 (35.1, 39.4)	92.2% (91.1%, 93.3%)	0% (0%, 0.1%)

	Inferior	No	Very Low	39.3% (-0.9%, 63.4%)	-0.7 (-1.1, -0.4)	68.6 (64.3, 72.8)	95.5% (94.7%, 96.3%)	3.4% (2.7%, 4.1%)
			Low	38.7% (16.4%, 54.7%)	-1.3 (-1.5, -1)	32.5 (30.5, 34.5)	95.3% (94.4%, 96.1%)	0% (0%, 0%)
			Medium	38.7% (12.3%, 57.5%)	-1.3 (-1.6, -1.1)	35.9 (33.7, 38)	94.9% (94.1%, 95.8%)	0.1% (0%, 0.2%)
			High	36.9% (16.7%, 52.5%)	-3.1 (-3.3, -2.8)	39.3 (36.9, 41.6)	91.6% (90.6%, 92.7%)	0% (0%, 0%)
			Very High	35.4% (17.9%, 52.1%)	-4.6 (-4.8, -4.4)	46.6 (44.1, 49.1)	85.2% (83.8%, 86.6%)	0% (0%, 0%)
		Yes	Very Low	39.6% (-0.9%, 63.4%)	-0.4 (-0.8, -0.1)	66.8 (62.7, 71)	95.6% (94.8%, 96.4%)	3.1% (2.4%, 3.8%)
			Low	39.7% (14.4%, 55.9%)	-0.3 (-0.5, 0)	29.9 (28.1, 31.7)	95.8% (95.1%, 96.6%)	0% (0%, 0.1%)
			Medium	39.9% (13.7%, 56.3%)	-0.1 (-0.4, 0.1)	32.9 (31, 34.8)	95.2% (94.4%, 96%)	0% (0%, 0.1%)
			High	39.3% (18.5%, 56.4%)	-0.7 (-0.9, -0.5)	28.3 (26.6, 30)	94.9% (94.1%, 95.8%)	0% (0%, 0%)
			Very High	38.6% (19.9%, 52.4%)	-1.4 (-1.6, -1.2)	26.5 (25, 28.1)	93.8% (92.9%, 94.7%)	0% (0%, 0%)
20%	Superior	Yes	Very Low	38.8% (4.6%, 65%)	-1.2 (-1.5, -0.9)	71.6 (67.1, 76.1)	95.5% (94.7%, 96.3%)	3.6% (2.9%, 4.4%)
			Low	38.1% (13.6%, 55.1%)	-1.9 (-2.1, -1.6)	38.2 (35.9, 40.5)	94.4% (93.5%, 95.3%)	0.2% (0%, 0.3%)
			Medium	37.3% (12.7%, 56.7%)	-2.7 (-3, -2.5)	45.6 (42.9, 48.3)	92.6% (91.5%, 93.6%)	0.1% (0%, 0.3%)
			High	34.1% (4.5%, 51.7%)	-5.9 (-6.1, -5.6)	71.4 (67.4, 75.4)	83.6% (82.1%, 85.1%)	0.5% (0.2%, 0.8%)
			Very High	30.9% (7.8%, 49.4%)	-9.1 (-9.3, -8.8)	117.6 (112.6, 122.5)	63.9% (62%, 65.8%)	0.8% (0.4%, 1.1%)
	Similar	No	Very Low	38.4% (-1.5%, 65.2%)	-1.6 (-1.9, -1.2)	75.5 (70.7, 80.4)	94.6% (93.7%, 95.4%)	5.1% (4.2%, 5.9%)
			Low	36.3% (14.4%, 53.9%)	-3.7 (-3.9, -3.5)	50.3 (47.4, 53.1)	90.5% (89.3%, 91.6%)	0.2% (0%, 0.4%)
			Medium	35.5% (7.5%, 54%)	-4.5 (-4.8, -4.3)	58.1 (54.8, 61.4)	89.4% (88.2%, 90.6%)	0.3% (0.1%, 0.5%)
			High	30.4% (5.8%, 48.5%)	-9.6 (-9.8, -9.4)	129 (123.5, 134.5)	62.7% (60.8%, 64.6%)	1.3% (0.9%, 1.8%)

			Very High	26.1% (4.4%, 47%)	-13.9 (-14.1, -13.6)	227.2 (220.1, 234.2)	29% (27.2%, 30.8%)	3.3% (2.6%, 4%)
		Yes	Very Low	39.3% (5.4%, 67.3%)	-0.7 (-1, -0.4)	69.9 (65.4, 74.3)	95.2% (94.4%, 96.1%)	3.6% (2.9%, 4.4%)
			Low	39.3% (15.1%, 56.9%)	-0.7 (-1, -0.5)	34.4 (32.5, 36.3)	95% (94.1%, 95.8%)	0% (0%, 0.1%)
			Medium	38.9% (12.9%, 57.1%)	-1.1 (-1.4, -0.9)	37.8 (35.4, 40.1)	94.7% (93.8%, 95.6%)	0.2% (0%, 0.3%)
			High	37.5% (17.1%, 56.6%)	-2.5 (-2.7, -2.3)	37.3 (35.1, 39.5)	93.7% (92.8%, 94.7%)	0% (0%, 0%)
			Very High	35.5% (11%, 51.2%)	-4.5 (-4.7, -4.3)	49.9 (47.1, 52.6)	88% (86.7%, 89.3%)	0.1% (0%, 0.3%)
	Inferior	No	Very Low	39.3% (6.7%, 66.6%)	-0.7 (-1.1, -0.4)	69.2 (65, 73.5)	95.4% (94.6%, 96.2%)	3.1% (2.4%, 3.8%)
			Low	38.9% (17.3%, 56.5%)	-1.1 (-1.4, -0.9)	34.1 (32.1, 36.1)	94.8% (93.9%, 95.7%)	0% (0%, 0%)
			Medium	38.5% (16.1%, 60.3%)	-1.5 (-1.7, -1.2)	34.9 (32.8, 37)	95.2% (94.3%, 96%)	0% (0%, 0.1%)
			High	37.2% (14.1%, 53.4%)	-2.8 (-3, -2.6)	37.2 (35, 39.4)	92.2% (91.1%, 93.2%)	0% (0%, 0.1%)
			Very High	35.5% (16.9%, 52.1%)	-4.5 (-4.7, -4.3)	47.3 (44.8, 49.8)	85% (83.6%, 86.4%)	0% (0%, 0%)
		Yes	Very Low	39.5% (4.5%, 66.6%)	-0.5 (-0.8, -0.2)	69.2 (64.9, 73.5)	95.2% (94.4%, 96%)	3.2% (2.5%, 3.8%)
			Low	39.6% (18.4%, 56.4%)	-0.4 (-0.7, -0.2)	32.6 (30.7, 34.5)	95.2% (94.3%, 96%)	0% (0%, 0%)
			Medium	39.3% (18.1%, 60.9%)	-0.7 (-0.9, -0.4)	33.8 (31.9, 35.8)	95.1% (94.3%, 96%)	0% (0%, 0%)
			High	39% (17.3%, 54.1%)	-1 (-1.2, -0.8)	27.9 (26.2, 29.6)	95.2% (94.4%, 96%)	0% (0%, 0%)
			Very High	38.1% (17.8%, 54.4%)	-1.9 (-2.1, -1.7)	29 (27.4, 30.7)	93% (92%, 94%)	0% (0%, 0%)

Table X. Mean estimate, bias, mean squared error (MSE), coverage, and false negative rate for vaccine efficacy (VE) estimates against any *Shigella* diarrhea from realistically sized trials for all 50 simulation scenarios when using active surveillance for infection and a stratified recurrent outcome regression model. Hybrid immunity was either not possible (no) or possible such that the first post-vaccination infection conferred additional immunity (yes). Bias was calculated as the average VE estimate for a set of simulations minus the true VE (60%). MSE was calculated as the average of the square of the estimate minus the true parameter. Coverage represents the percent of 95% confidence intervals (CIs) that contained the true VE (60%), and the false negative rate indicates the percent of 95% CIs that incorrectly indicated a non-significant effect.

VE against infection	Strength of infection-acquired immunity	Hybrid immunity	Force of infection	Mean VE estimate (range)	Bias (95% CI)	MSE (95% CI)	Coverage (95% CI)	False negatives (95% CI)
0%	Superior	Yes	Very Low	33.1% (-8.1%, 57.5%)	-6.9 (-7.2, -6.5)	120.8 (113.9, 127.7)	89% (87.8%, 90.2%)	11.5% (10.3%, 12.8%)
			Low	31.1% (6.5%, 49.1%)	-8.9 (-9.1, -8.6)	116.6 (111.6, 121.6)	68.2% (66.4%, 70.1%)	1% (0.6%, 1.4%)
			Medium	30.1% (5.4%, 51.9%)	-9.9 (-10.2, -9.7)	141.8 (135.7, 147.8)	64.6% (62.8%, 66.5%)	2.7% (2%, 3.3%)
			High	25% (-3.8%, 48.2%)	-15 (-15.3, -14.8)	269 (260.4, 277.5)	27% (25.2%, 28.7%)	7% (6%, 8%)
			Very High	20.5% (-3.4%, 40%)	-19.5 (-19.7, -19.2)	417.6 (407.7, 427.6)	5.6% (4.7%, 6.5%)	14.9% (13.5%, 16.3%)
	Similar	No	Very Low	38% (4.2%, 62%)	-2 (-2.4, -1.7)	72.5 (68.1, 76.9)	94.6% (93.8%, 95.5%)	4.6% (3.8%, 5.4%)
			Low	34.5% (13.1%, 54.2%)	-5.5 (-5.8, -5.3)	66.4 (62.9, 69.8)	84.2% (82.7%, 85.6%)	0.3% (0.1%, 0.5%)
			Medium	33.5% (1.3%, 51.5%)	-6.5 (-6.7, -6.3)	79.8 (75.7, 83.9)	82.6% (81.1%, 84%)	0.6% (0.3%, 0.9%)
			High	25.9% (0.4%, 47.4%)	-14.1 (-14.3, -13.8)	229.2 (222.5, 235.8)	22.1% (20.5%, 23.7%)	1.5% (1%, 2%)
			Very High	20.2% (-5.5%, 34.7%)	-19.8 (-20, -19.6)	414.4 (406.6, 422.1)	0.2% (0%, 0.4%)	3.1% (2.4%, 3.8%)
		Yes	Very Low	39.5% (7.4%, 62.2%)	-0.5 (-0.9, -0.2)	66.2 (62.3, 70.1)	96.2% (95.4%, 96.9%)	3.2% (2.5%, 3.9%)
			Low	39.1% (16.9%, 58.6%)	-0.9 (-1.1, -0.7)	32.2 (30.3, 34.1)	95.3% (94.4%, 96.1%)	0% (0%, 0%)
			Medium	39% (16.7%, 56.6%)	-1 (-1.2, -0.7)	35.4 (33.4, 37.4)	95.3% (94.5%, 96.1%)	0.1% (0%, 0.2%)
			High	36.3% (14.8%, 53.9%)	-3.7 (-3.9, -3.4)	45.4 (42.9, 48)	89.3% (88.1%, 90.5%)	0.1% (0%, 0.3%)
			Very High	32.8% (12.4%, 49.9%)	-7.2 (-7.4, -6.9)	80.2 (76.5, 83.8)	72.1% (70.4%, 73.9%)	0.1% (0%, 0.3%)

	Inferior	No	Very Low	39.2% (1%, 62.6%)	-0.8 (-1.2, -0.5)	67.3 (63.2, 71.5)	95.4% (94.6%, 96.3%)	3.4% (2.7%, 4.1%)
			Low	38.3% (15.8%, 54.4%)	-1.7 (-2, -1.5)	33 (31, 35)	94.6% (93.8%, 95.5%)	0% (0%, 0%)
			Medium	38% (14.5%, 55.8%)	-2 (-2.2, -1.8)	36.6 (34.4, 38.9)	93.8% (92.8%, 94.7%)	0.1% (0%, 0.3%)
			High	35.3% (17.4%, 50.8%)	-4.7 (-4.9, -4.5)	48.6 (46, 51.2)	84.3% (82.9%, 85.7%)	0% (0%, 0%)
			Very High	32.5% (16.7%, 47.6%)	-7.5 (-7.7, -7.3)	77.4 (74.3, 80.6)	61.6% (59.7%, 63.5%)	0% (0%, 0%)
		Yes	Very Low	39.5% (1%, 62.6%)	-0.5 (-0.8, -0.2)	65.2 (61.2, 69.2)	95.5% (94.7%, 96.3%)	3% (2.3%, 3.6%)
			Low	39.5% (18.5%, 55%)	-0.5 (-0.7, -0.3)	28.8 (27.1, 30.5)	95.7% (94.9%, 96.5%)	0% (0%, 0%)
			Medium	39.5% (15.3%, 56.4%)	-0.5 (-0.7, -0.3)	31.8 (30, 33.7)	94.9% (94.1%, 95.8%)	0% (0%, 0.1%)
			High	38.3% (17.7%, 54.9%)	-1.7 (-1.9, -1.5)	27.6 (25.9, 29.3)	93.8% (92.8%, 94.7%)	0% (0%, 0%)
			Very High	36.6% (22.2%, 50.2%)	-3.4 (-3.6, -3.3)	31.1 (29.5, 32.8)	88.1% (86.9%, 89.4%)	0% (0%, 0%)
20%	Superior	Yes	Very Low	38.8% (4.2%, 65.1%)	-1.2 (-1.6, -0.9)	71.7 (67.2, 76.2)	95.3% (94.5%, 96.1%)	3.9% (3.2%, 4.7%)
			Low	38% (15.6%, 54.6%)	-2 (-2.3, -1.8)	38.1 (35.8, 40.3)	94.4% (93.5%, 95.3%)	0.1% (0%, 0.3%)
			Medium	37% (14%, 57.7%)	-3 (-3.2, -2.7)	46.1 (43.4, 48.8)	92.7% (91.7%, 93.7%)	0.1% (0%, 0.2%)
			High	33.6% (6.8%, 50.3%)	-6.4 (-6.7, -6.2)	75.9 (71.9, 79.8)	79.5% (77.9%, 81.1%)	0.4% (0.1%, 0.6%)
			Very High	29.6% (6.7%, 47.5%)	-10.4 (-10.6, -10.1)	139 (133.9, 144.2)	49.9% (47.9%, 51.8%)	0.6% (0.3%, 1%)
	Similar	No	Very Low	38.1% (-1.4%, 65.8%)	-1.9 (-2.2, -1.6)	76.8 (71.9, 81.8)	94.1% (93.2%, 95%)	5.3% (4.4%, 6.2%)
			Low	35.2% (12.1%, 53.6%)	-4.8 (-5, -4.5)	59.3 (56.1, 62.6)	86.8% (85.5%, 88.1%)	0.3% (0.1%, 0.5%)
			Medium	34% (7.9%, 52.5%)	-6 (-6.2, -5.7)	73 (69.2, 76.8)	84.4% (83%, 85.8%)	0.4% (0.2%, 0.6%)
			High	27.3% (3.6%, 44.7%)	-12.7 (-12.9, -12.4)	196.6 (189.8, 203.4)	37.9% (36%, 39.8%)	2.5% (1.9%, 3.1%)

			Very High	21.7% (2.7%, 41.5%)	-18.3 (-18.5, -18.1)	366.3 (357.9, 374.8)	4.4% (3.6%, 5.2%)	6.8% (5.8%, 7.8%)
		Yes	Very Low	39.4% (5.2%, 67.4%)	-0.6 (-0.9, -0.3)	68.9 (64.6, 73.3)	94.8% (94%, 95.7%)	3.5% (2.8%, 4.2%)
			Low	39.5% (18.6%, 55.6%)	-0.5 (-0.7, -0.3)	32.3 (30.6, 34.1)	95% (94.1%, 95.9%)	0% (0%, 0%)
			Medium	39.1% (12.2%, 57.6%)	-0.9 (-1.2, -0.7)	36.1 (33.8, 38.3)	94.2% (93.3%, 95.2%)	0% (0%, 0.1%)
			High	37.6% (19.6%, 56.3%)	-2.4 (-2.6, -2.2)	34.5 (32.5, 36.5)	92.8% (91.8%, 93.8%)	0% (0%, 0%)
			Very High	34.9% (13.6%, 49.7%)	-5.1 (-5.3, -4.9)	51.5 (48.8, 54.3)	84.2% (82.8%, 85.7%)	0% (0%, 0.1%)
	Inferior	No	Very Low	39.2% (5.5%, 66.6%)	-0.8 (-1.1, -0.5)	69 (64.7, 73.3)	95% (94.1%, 95.9%)	2.9% (2.3%, 3.6%)
			Low	38.6% (18.3%, 57%)	-1.4 (-1.6, -1.2)	33.8 (31.8, 35.8)	94.6% (93.7%, 95.4%)	0% (0%, 0%)
			Medium	38.2% (15%, 59%)	-1.8 (-2.1, -1.6)	34.8 (32.7, 36.8)	94.7% (93.8%, 95.6%)	0% (0%, 0.1%)
			High	36.1% (16.7%, 51.5%)	-3.9 (-4.1, -3.7)	40.6 (38.3, 42.8)	87.8% (86.6%, 89.1%)	0% (0%, 0%)
			Very High	33.7% (17.4%, 48%)	-6.3 (-6.4, -6.1)	60.4 (57.7, 63)	71.1% (69.3%, 72.9%)	0% (0%, 0%)
		Yes	Very Low	39.6% (3.7%, 66.6%)	-0.4 (-0.7, -0.1)	68.5 (64.2, 72.8)	95% (94.2%, 95.9%)	2.7% (2%, 3.3%)
			Low	39.6% (20.2%, 56.7%)	-0.4 (-0.6, -0.2)	31.1 (29.3, 33)	95.1% (94.2%, 95.9%)	0% (0%, 0%)
			Medium	39.4% (17.6%, 60.4%)	-0.6 (-0.9, -0.4)	32 (30.1, 33.8)	95.2% (94.3%, 96%)	0% (0%, 0%)
			High	38.8% (19.6%, 55.1%)	-1.2 (-1.4, -1)	24.6 (23.1, 26.1)	94.9% (94%, 95.7%)	0% (0%, 0%)
			Very High	37.3% (18.3%, 52.1%)	-2.7 (-2.9, -2.6)	27.6 (26, 29.2)	90% (88.9%, 91.2%)	0% (0%, 0%)

Table Y. Mean estimate, bias, mean squared error (MSE), coverage, and false negative rate for vaccine efficacy (VE) estimates against any *Shigella* diarrhea from realistically sized trials for all 50 simulation scenarios when using symptom-based reporting and a stratified recurrent outcome regression model. Hybrid immunity was either not possible (no) or possible such that the first post-vaccination infection conferred additional immunity (yes). Bias was calculated as the average VE estimate for a set of simulations minus the true VE (60%). MSE was calculated as the average of the square of the estimate minus the true parameter. Coverage represents the percent of 95% confidence intervals (CIs) that contained the true VE (60%), and the false negative rate indicates the percent of 95% CIs that incorrectly indicated a non-significant effect.

VE against infection	Strength of infection-acquired immunity	Hybrid immunity	Force of infection	Mean VE estimate (range)	Bias (95% CI)	MSE (95% CI)	Coverage (95% CI)	False negatives (95% CI)
0%	Superior	Yes	Very Low	33.3% (-7.9%, 57.7%)	-6.7 (-7, -6.3)	117.7 (110.8, 124.5)	89.7% (88.5%, 90.9%)	11.3% (10%, 12.5%)
			Low	31.8% (7%, 50%)	-8.2 (-8.4, -7.9)	104.7 (100, 109.4)	72.2% (70.5%, 74%)	0.6% (0.3%, 1%)
			Medium	30.9% (6.6%, 52.4%)	-9.1 (-9.4, -8.9)	125.8 (120.1, 131.4)	69.4% (67.6%, 71.2%)	2.2% (1.7%, 2.8%)
			High	26.4% (-0.9%, 49.3%)	-13.6 (-13.8, -13.3)	226.5 (218.8, 234.2)	34.6% (32.7%, 36.5%)	4.8% (4%, 5.7%)
			Very High	22.2% (-0.7%, 41.6%)	-17.8 (-18, -17.6)	354.3 (345.3, 363.3)	9.4% (8.2%, 10.5%)	9.6% (8.4%, 10.8%)
	Similar	No	Very Low	38.1% (4.3%, 62.2%)	-1.9 (-2.2, -1.6)	71.9 (67.6, 76.3)	94.9% (94.1%, 95.8%)	4.4% (3.6%, 5.2%)
			Low	34.9% (14.2%, 54.7%)	-5.1 (-5.3, -4.9)	61.6 (58.3, 64.9)	86% (84.6%, 87.4%)	0.2% (0%, 0.4%)
			Medium	34% (1.5%, 52.2%)	-6 (-6.2, -5.7)	73.1 (69.3, 77)	84.6% (83.2%, 86%)	0.4% (0.1%, 0.6%)
			High	26.8% (1.6%, 48.4%)	-13.2 (-13.4, -13)	206.4 (200.1, 212.7)	27.6% (25.8%, 29.4%)	1.2% (0.8%, 1.6%)
			Very High	21.1% (-5.1%, 36.2%)	-18.9 (-19.1, -18.8)	381.8 (374.3, 389.2)	0.4% (0.2%, 0.7%)	2.2% (1.6%, 2.8%)
		Yes	Very Low	39.7% (7.6%, 62.6%)	-0.3 (-0.6, 0)	65.6 (61.7, 69.4)	96% (95.2%, 96.7%)	2.9% (2.3%, 3.6%)
			Low	39.8% (17.8%, 59.5%)	-0.2 (-0.4, 0)	31 (29.2, 32.8)	95.1% (94.3%, 96%)	0% (0%, 0%)
			Medium	39.9% (17.9%, 56.7%)	-0.1 (-0.3, 0.1)	33.7 (31.9, 35.6)	95.4% (94.5%, 96.2%)	0% (0%, 0%)
			High	37.9% (15.9%, 55.6%)	-2.1 (-2.3, -1.9)	35 (33, 37)	93.4% (92.4%, 94.4%)	0% (0%, 0.1%)
			Very High	34.8% (14.5%, 51.9%)	-5.2 (-5.4, -5)	55.2 (52.4, 58.1)	82.6% (81.1%, 84%)	0% (0%, 0.1%)

	Inferior	No	Very Low	39.2% (0.9%, 62.9%)	-0.8 (-1.1, -0.5)	67.4 (63.2, 71.6)	95.4% (94.5%, 96.2%)	3.3% (2.6%, 4%)
			Low	38.4% (15.5%, 54.3%)	-1.6 (-1.8, -1.4)	32.7 (30.7, 34.7)	94.6% (93.8%, 95.5%)	0% (0%, 0%)
			Medium	38.2% (15.3%, 55.8%)	-1.8 (-2, -1.6)	35.9 (33.8, 38.1)	94.1% (93.2%, 95%)	0.1% (0%, 0.2%)
			High	35.7% (17.8%, 51%)	-4.3 (-4.5, -4.1)	45.1 (42.7, 47.6)	86% (84.6%, 87.4%)	0% (0%, 0%)
			Very High	33.1% (16.8%, 47.5%)	-6.9 (-7, -6.7)	68.6 (65.7, 71.5)	67.3% (65.5%, 69.2%)	0% (0%, 0%)
		Yes	Very Low	39.6% (0.9%, 62.9%)	-0.4 (-0.7, -0.1)	65.2 (61.2, 69.2)	95.5% (94.7%, 96.3%)	2.9% (2.3%, 3.6%)
			Low	39.7% (18.1%, 54.9%)	-0.3 (-0.5, -0.1)	28.6 (27, 30.3)	95.6% (94.8%, 96.4%)	0% (0%, 0%)
			Medium	39.7% (16.5%, 56.4%)	-0.3 (-0.5, 0)	31.5 (29.6, 33.3)	95% (94.2%, 95.9%)	0% (0%, 0%)
			High	38.8% (18.5%, 55.6%)	-1.2 (-1.4, -1)	26.1 (24.5, 27.8)	94.2% (93.3%, 95.2%)	0% (0%, 0%)
			Very High	37.3% (22.6%, 50.4%)	-2.7 (-2.9, -2.5)	26.8 (25.3, 28.2)	91.2% (90%, 92.3%)	0% (0%, 0%)
20%	Superior	Yes	Very Low	38.6% (3.8%, 65.1%)	-1.4 (-1.7, -1)	72.4 (67.8, 76.9)	95.2% (94.3%, 96%)	4% (3.2%, 4.7%)
			Low	37.6% (15%, 54.2%)	-2.4 (-2.6, -2.2)	40 (37.7, 42.4)	93.6% (92.7%, 94.6%)	0.2% (0%, 0.4%)
			Medium	36.6% (13%, 57.2%)	-3.4 (-3.7, -3.2)	49.6 (46.8, 52.5)	91.4% (90.3%, 92.5%)	0.1% (0%, 0.2%)
			High	32.4% (6.3%, 49.6%)	-7.6 (-7.8, -7.4)	93.5 (89, 98)	73.6% (71.9%, 75.4%)	0.6% (0.3%, 0.9%)
			Very High	27.9% (6.1%, 47.6%)	-12.1 (-12.3, -11.8)	179 (173, 184.9)	38.4% (36.5%, 40.3%)	1.2% (0.8%, 1.6%)
	Similar	No	Very Low	38.1% (-1.5%, 65.5%)	-1.9 (-2.3, -1.6)	77.3 (72.3, 82.2)	94% (93.1%, 95%)	5.3% (4.4%, 6.2%)
			Low	35% (12%, 53.4%)	-5 (-5.2, -4.7)	61.3 (58, 64.6)	86% (84.7%, 87.4%)	0.3% (0.1%, 0.5%)
			Medium	33.8% (8.4%, 51.7%)	-6.2 (-6.4, -6)	76.3 (72.3, 80.2)	83% (81.6%, 84.5%)	0.4% (0.2%, 0.7%)
			High	26.9% (2.8%, 44.3%)	-13.1 (-13.4, -12.9)	209.4 (202.4, 216.5)	35% (33.1%, 36.8%)	3% (2.4%, 3.7%)

			Very High	21.1% (2.1%, 41.3%)	-18.9 (-19.1, -18.7)	389.4 (380.6, 398.2)	3.8% (3%, 4.5%)	8.6% (7.5%, 9.7%)
		Yes	Very Low	39.3% (4.9%, 67.4%)	-0.7 (-1, -0.4)	69.4 (65, 73.8)	94.8% (93.9%, 95.7%)	3.7% (2.9%, 4.4%)
			Low	39.2% (17.5%, 55.8%)	-0.8 (-1.1, -0.6)	33.1 (31.3, 34.9)	95.2% (94.3%, 96%)	0% (0%, 0%)
			Medium	38.7% (11.8%, 57.3%)	-1.3 (-1.6, -1.1)	37.5 (35.1, 39.8)	94.2% (93.3%, 95.1%)	0.1% (0%, 0.2%)
			High	36.7% (18.1%, 55.7%)	-3.3 (-3.6, -3.1)	40.8 (38.5, 43.2)	90.3% (89.1%, 91.4%)	0% (0%, 0%)
			Very High	33.5% (11.9%, 49.6%)	-6.5 (-6.7, -6.3)	68.7 (65.5, 72)	76.8% (75.1%, 78.5%)	0.1% (0%, 0.3%)
	Inferior	No	Very Low	39.2% (5.4%, 66.8%)	-0.8 (-1.1, -0.5)	69 (64.7, 73.3)	95.1% (94.3%, 96%)	2.9% (2.2%, 3.5%)
			Low	38.5% (17.6%, 57.4%)	-1.5 (-1.7, -1.2)	33.9 (31.9, 35.9)	94.4% (93.5%, 95.3%)	0% (0%, 0%)
			Medium	38.1% (14.9%, 59.2%)	-1.9 (-2.1, -1.7)	35.1 (33, 37.2)	94.7% (93.8%, 95.6%)	0% (0%, 0.1%)
			High	36% (16.9%, 51.9%)	-4 (-4.2, -3.8)	42.3 (39.9, 44.6)	87.4% (86.1%, 88.7%)	0% (0%, 0%)
			Very High	33.5% (17.4%, 47.3%)	-6.5 (-6.6, -6.3)	63.3 (60.6, 66.1)	69.8% (68%, 71.6%)	0% (0%, 0%)
		Yes	Very Low	39.6% (3.7%, 66.8%)	-0.4 (-0.8, -0.1)	68.5 (64.2, 72.8)	95.2% (94.3%, 96%)	2.6% (2%, 3.3%)
			Low	39.5% (20.5%, 57%)	-0.5 (-0.7, -0.3)	31.2 (29.4, 33.1)	95.1% (94.2%, 95.9%)	0% (0%, 0%)
			Medium	39.3% (17.7%, 60.5%)	-0.7 (-1, -0.5)	32.2 (30.3, 34.1)	95.2% (94.4%, 96.1%)	0% (0%, 0%)
			High	38.6% (19.4%, 55.4%)	-1.4 (-1.6, -1.2)	25.5 (23.9, 27.1)	94.5% (93.6%, 95.4%)	0% (0%, 0%)
			Very High	37% (17.6%, 51.2%)	-3 (-3.1, -2.8)	29.3 (27.6, 30.9)	89.8% (88.6%, 90.9%)	0% (0%, 0%)

Table Z. Mean estimate, bias, mean squared error (MSE), coverage, and false negative rate for vaccine efficacy (VE) estimates against any *Shigella* diarrhea from realistically sized trials for all 50 simulation scenarios when using active surveillance for infection and a crude recurrent outcome regression model. Hybrid immunity was either not possible (no) or possible such that the first post-vaccination infection conferred additional immunity (yes). Bias was calculated as the average VE estimate for a set of simulations minus the true VE (60%). MSE was calculated as the average of the square of the estimate minus the true parameter. Coverage represents the percent of 95% confidence intervals (CIs) that contained the true VE (60%), and the false negative rate indicates the percent of 95% CIs that incorrectly indicated a non-significant effect.

VE against infection	Strength of infection-acquired immunity	Hybrid immunity	Force of infection	Mean VE estimate (range)	Bias (95% CI)	MSE (95% CI)	Coverage (95% CI)	False negatives (95% CI)
0%	Superior	Yes	Very Low	33.1% (-7.6%, 57.2%)	-6.9 (-7.2, -6.5)	119.8 (112.9, 126.6)	88.8% (87.6%, 90.1%)	11.3% (10%, 12.5%)
0%	Superior	Yes	Low	31.2% (6.9%, 49%)	-8.8 (-9.1, -8.6)	114.8 (110, 119.7)	67.3% (65.4%, 69.1%)	0.7% (0.4%, 1%)
0%	Superior	Yes	Medium	30.1% (6.4%, 51.4%)	-9.9 (-10.2, -9.7)	139.4 (133.6, 145.3)	62.6% (60.7%, 64.5%)	2.3% (1.7%, 2.9%)
0%	Superior	Yes	High	25% (-1.1%, 47.8%)	-15 (-15.2, -14.8)	263.8 (255.8, 271.8)	23% (21.3%, 24.6%)	5.2% (4.3%, 6%)
0%	Superior	Yes	Very High	20.5% (-0.9%, 38.8%)	-19.5 (-19.7, -19.2)	412.4 (403.2, 421.7)	3.6% (2.8%, 4.3%)	10.8% (9.6%, 12%)
0%	Similar	No	Very Low	38% (4.3%, 61.9%)	-2 (-2.4, -1.7)	71.9 (67.6, 76.3)	94.6% (93.7%, 95.5%)	4.4% (3.6%, 5.2%)
0%	Similar	No	Low	34.5% (13.3%, 53.8%)	-5.5 (-5.8, -5.3)	65.5 (62.1, 68.9)	83.9% (82.4%, 85.3%)	0.3% (0.1%, 0.5%)
0%	Similar	No	Medium	33.5% (1.3%, 51.7%)	-6.5 (-6.8, -6.3)	78.9 (74.8, 82.9)	82.4% (80.9%, 83.9%)	0.3% (0.1%, 0.5%)
0%	Similar	No	High	25.9% (1.6%, 46.8%)	-14.1 (-14.3, -13.8)	227.5 (221, 234)	20.5% (18.9%, 22.1%)	1.3% (0.8%, 1.7%)
0%	Similar	No	Very High	20.2% (-5.2%, 34.6%)	-19.8 (-20, -19.6)	413.2 (405.7, 420.8)	0.2% (0%, 0.3%)	2.8% (2.2%, 3.5%)
0%	Similar	Yes	Very Low	39.5% (7.6%, 62%)	-0.5 (-0.9, -0.2)	65.2 (61.4, 69.1)	96.1% (95.3%, 96.8%)	2.9% (2.3%, 3.6%)
0%	Similar	Yes	Low	39.1% (17.3%, 58.3%)	-0.9 (-1.1, -0.7)	31 (29.2, 32.9)	95.3% (94.5%, 96.1%)	0% (0%, 0%)
0%	Similar	Yes	Medium	39.1% (17.4%, 55.8%)	-0.9 (-1.2, -0.7)	33.7 (31.8, 35.5)	95.2% (94.4%, 96%)	0% (0%, 0%)
0%	Similar	Yes	High	36.4% (15.2%, 53.3%)	-3.6 (-3.8, -3.4)	42 (39.7, 44.4)	89% (87.8%, 90.2%)	0% (0%, 0.1%)
0%	Similar	Yes	Very High	32.9% (13.4%, 49.4%)	-7.1 (-7.3, -6.9)	76.9 (73.5, 80.2)	69.6% (67.8%, 71.4%)	0% (0%, 0.1%)

	Inferior	No	Very Low	39.1%<br>(0.9%, 62.6%)	-0.9<br>(-1.2, -0.5)	67.2<br>(63, 71.3)	95.4%<br>(94.6%, 96.3%)	3.3%<br>(2.6%, 4%)
			Low	38.3%<br>(15.7%, 54.4%)	-1.7<br>(-2, -1.5)	32.8<br>(30.8, 34.8)	94.7%<br>(93.8%, 95.6%)	0%<br>(0%, 0%)
			Medium	38%<br>(15.1%, 55.9%)	-2<br>(-2.2, -1.8)	36.3<br>(34.1, 38.5)	93.7%<br>(92.7%, 94.6%)	0.1%<br>(0%, 0.2%)
			High	35.3%<br>(17.8%, 50.6%)	-4.7<br>(-4.9, -4.5)	48.1<br>(45.5, 50.6)	84.2%<br>(82.8%, 85.7%)	0%<br>(0%, 0%)
			Very High	32.5%<br>(16.9%, 47.6%)	-7.5<br>(-7.7, -7.3)	76.8<br>(73.7, 79.9)	60.5%<br>(58.6%, 62.4%)	0%<br>(0%, 0%)
		Yes	Very Low	39.5%<br>(0.9%, 62.6%)	-0.5<br>(-0.8, -0.2)	65<br>(61, 69)	95.5%<br>(94.7%, 96.3%)	2.9%<br>(2.2%, 3.5%)
			Low	39.5%<br>(18.4%, 55%)	-0.5<br>(-0.7, -0.3)	28.5<br>(26.8, 30.1)	95.8%<br>(95%, 96.5%)	0%<br>(0%, 0%)
			Medium	39.5%<br>(16%, 56.4%)	-0.5<br>(-0.7, -0.3)	31.4<br>(29.5, 33.2)	94.9%<br>(94.1%, 95.8%)	0%<br>(0%, 0%)
			High	38.3%<br>(18.2%, 54.9%)	-1.7<br>(-1.9, -1.5)	27<br>(25.3, 28.6)	93.8%<br>(92.9%, 94.7%)	0%<br>(0%, 0%)
			Very High	36.6%<br>(22.2%, 50.2%)	-3.4<br>(-3.6, -3.3)	30.5<br>(28.9, 32.1)	88%<br>(86.7%, 89.2%)	0%<br>(0%, 0%)
20%	Superior	Yes	Very Low	38.4%<br>(3.8%, 64.7%)	-1.6<br>(-1.9, -1.3)	72.4<br>(67.8, 76.9)	95%<br>(94.1%, 95.9%)	4%<br>(3.2%, 4.7%)
			Low	36.9%<br>(14.6%, 53.1%)	-3.1<br>(-3.4, -2.9)	43.2<br>(40.7, 45.7)	91.7%<br>(90.6%, 92.8%)	0.2%<br>(0%, 0.4%)
			Medium	35.7%<br>(12.6%, 56.1%)	-4.3<br>(-4.6, -4.1)	55.2<br>(52.1, 58.2)	88.6%<br>(87.3%, 89.8%)	0.1%<br>(0%, 0.2%)
			High	30.8%<br>(5.9%, 46.8%)	-9.2<br>(-9.4, -9)	117.5<br>(112.5, 122.4)	59.9%<br>(58%, 61.8%)	0.6%<br>(0.3%, 0.9%)
			Very High	26.1%<br>(5.1%, 44.7%)	-13.9<br>(-14.1, -13.7)	224.6<br>(218.1, 231.1)	22%<br>(20.3%, 23.6%)	1.4%<br>(0.9%, 1.8%)
	Similar	No	Very Low	37.9%<br>(-1.7%, 65.3%)	-2.1<br>(-2.4, -1.8)	77.2<br>(72.2, 82.2)	93.9%<br>(92.9%, 94.8%)	5.2%<br>(4.3%, 6.1%)
			Low	34.6%<br>(12%, 52.7%)	-5.4<br>(-5.6, -5.2)	64.9<br>(61.5, 68.3)	83.9%<br>(82.5%, 85.4%)	0.3%<br>(0.1%, 0.5%)
			Medium	33.3%<br>(8%, 51.6%)	-6.7<br>(-7, -6.5)	81.8<br>(77.7, 85.8)	80.6%<br>(79.1%, 82.2%)	0.4%<br>(0.2%, 0.7%)
			High	26.1%<br>(2.5%, 43.4%)	-13.9<br>(-14.2, -13.7)	229.4<br>(222.2, 236.7)	27%<br>(25.3%, 28.8%)	3.1%<br>(2.4%, 3.8%)

			Very High	20.3% (1.7%, 39.8%)	-19.7 (-19.9, -19.5)	418.9 (410, 427.8)	1.9% (1.3%, 2.4%)	9.4% (8.2%, 10.5%)
		Yes	Very Low	39.1% (4.9%, 66.9%)	-0.9 (-1.2, -0.6)	69.2 (64.7, 73.6)	94.9% (94.1%, 95.8%)	3.6% (2.9%, 4.4%)
			Low	38.5% (17.1%, 54.8%)	-1.5 (-1.7, -1.3)	34 (32.1, 35.9)	94.6% (93.8%, 95.5%)	0% (0%, 0%)
			Medium	37.9% (11.1%, 55.8%)	-2.1 (-2.4, -1.9)	39.1 (36.6, 41.5)	93.4% (92.4%, 94.4%)	0.1% (0%, 0.3%)
			High	35.1% (17.1%, 54%)	-4.9 (-5.1, -4.7)	51.8 (49.1, 54.5)	84% (82.6%, 85.5%)	0% (0%, 0%)
			Very High	31.6% (11.4%, 46.6%)	-8.4 (-8.6, -8.2)	94.8 (91, 98.6)	58.8% (56.9%, 60.8%)	0.2% (0%, 0.3%)
	Inferior	No	Very Low	39.1% (5.4%, 66.5%)	-0.9 (-1.2, -0.5)	68.8 (64.5, 73.1)	95.1% (94.2%, 95.9%)	2.9% (2.2%, 3.5%)
			Low	38.4% (17.7%, 57%)	-1.6 (-1.8, -1.4)	34 (32, 36.1)	94.3% (93.4%, 95.2%)	0% (0%, 0%)
			Medium	37.9% (14.9%, 59.1%)	-2.1 (-2.3, -1.9)	35.3 (33.2, 37.4)	94.3% (93.4%, 95.2%)	0% (0%, 0.1%)
			High	35.6% (16.3%, 51.6%)	-4.4 (-4.6, -4.2)	44.4 (42, 46.8)	85.1% (83.7%, 86.5%)	0% (0%, 0%)
			Very High	33.1% (16.8%, 47.2%)	-6.9 (-7.1, -6.7)	68.2 (65.3, 71)	65.7% (63.8%, 67.5%)	0% (0%, 0%)
		Yes	Very Low	39.5% (3.6%, 66.5%)	-0.5 (-0.8, -0.2)	68.3 (64.1, 72.6)	95.1% (94.3%, 96%)	2.6% (2%, 3.3%)
			Low	39.3% (20.2%, 56.6%)	-0.7 (-0.9, -0.4)	31.2 (29.3, 33)	95.2% (94.3%, 96%)	0% (0%, 0%)
			Medium	39% (17.6%, 60.4%)	-1 (-1.2, -0.7)	32.1 (30.2, 34)	95.2% (94.4%, 96.1%)	0% (0%, 0%)
			High	38.1% (19%, 54.7%)	-1.9 (-2.1, -1.7)	26.4 (24.8, 28.1)	94.1% (93.2%, 95%)	0% (0%, 0%)
			Very High	36.4% (17.5%, 50.6%)	-3.6 (-3.8, -3.4)	32.5 (30.7, 34.3)	86.4% (85.1%, 87.8%)	0% (0%, 0%)

Table AA. Mean estimate, bias, mean squared error (MSE), coverage, and false negative rate for vaccine efficacy (VE) estimates against any *Shigella* diarrhea from realistically sized trials for all 50 simulation scenarios when using symptom-based reporting and a crude recurrent outcome regression model. Hybrid immunity was either not possible (no) or possible such that the first post-vaccination infection conferred additional immunity (yes). Bias was calculated as the average VE estimate for a set of simulations minus the true VE (60%). MSE was calculated as the average of the square of the estimate minus the true parameter. Coverage represents the percent of 95% confidence intervals (CIs) that contained the true VE (60%), and the false negative rate indicates the percent of 95% CIs that incorrectly indicated a non-significant effect.

VE against infection	Strength of infection-acquired immunity	Hybrid immunity	Force of infection	Mean VE estimate (range)	Bias (95% CI)	MSE (95% CI)	Coverage (95% CI)	False negatives (95% CI)
0%	Superior	Yes	Very Low	33.1% (-7.5%, 57.2%)	-6.9 (-7.2, -6.5)	119.7 (112.9, 126.6)	88.8% (87.6%, 90.1%)	11.2% (10%, 12.5%)
0%	Superior	Yes	Low	31.2% (6.9%, 49%)	-8.8 (-9.1, -8.6)	114.7 (109.8, 119.6)	67.3% (65.4%, 69.1%)	0.7% (0.4%, 1%)
0%	Superior	Yes	Medium	30.1% (6.4%, 51.4%)	-9.9 (-10.2, -9.7)	139.2 (133.4, 145.1)	62.7% (60.8%, 64.6%)	2.3% (1.7%, 2.9%)
0%	Superior	Yes	High	25% (-1%, 47.8%)	-15 (-15.2, -14.7)	263.2 (255.2, 271.2)	23% (21.4%, 24.6%)	5.1% (4.3%, 6%)
0%	Superior	Yes	Very High	20.6% (-0.8%, 38.8%)	-19.4 (-19.7, -19.2)	411.5 (402.2, 420.7)	3.6% (2.9%, 4.4%)	10.7% (9.5%, 11.9%)
0%	Similar	No	Very Low	38% (4.3%, 61.9%)	-2 (-2.4, -1.7)	71.9 (67.6, 76.3)	94.6% (93.7%, 95.5%)	4.3% (3.5%, 5.1%)
0%	Similar	No	Low	34.5% (13.4%, 53.8%)	-5.5 (-5.8, -5.3)	65.4 (62, 68.8)	83.9% (82.4%, 85.3%)	0.3% (0.1%, 0.5%)
0%	Similar	No	Medium	33.5% (1.3%, 51.7%)	-6.5 (-6.7, -6.3)	78.7 (74.7, 82.7)	82.4% (80.9%, 83.9%)	0.3% (0.1%, 0.5%)
0%	Similar	No	High	26% (1.6%, 46.8%)	-14 (-14.3, -13.8)	226.9 (220.4, 233.4)	20.5% (18.9%, 22.1%)	1.3% (0.8%, 1.7%)
0%	Similar	No	Very High	20.2% (-5.2%, 34.7%)	-19.8 (-19.9, -19.6)	412.1 (404.6, 419.7)	0.2% (0%, 0.3%)	2.8% (2.1%, 3.4%)
0%	Similar	Yes	Very Low	39.5% (7.6%, 62%)	-0.5 (-0.9, -0.2)	65.2 (61.4, 69.1)	96.1% (95.3%, 96.8%)	2.9% (2.3%, 3.6%)
0%	Similar	Yes	Low	39.1% (17.3%, 58.3%)	-0.9 (-1.1, -0.7)	31 (29.1, 32.8)	95.3% (94.4%, 96.1%)	0% (0%, 0%)
0%	Similar	Yes	Medium	39.1% (17.5%, 55.8%)	-0.9 (-1.1, -0.7)	33.6 (31.8, 35.5)	95.2% (94.4%, 96.1%)	0% (0%, 0%)
0%	Similar	Yes	High	36.4% (15.2%, 53.4%)	-3.6 (-3.8, -3.4)	41.9 (39.5, 44.2)	89.1% (87.9%, 90.3%)	0% (0%, 0.1%)
0%	Similar	Yes	Very High	32.9% (13.4%, 49.4%)	-7.1 (-7.3, -6.9)	76.4 (73, 79.7)	69.7% (67.9%, 71.5%)	0% (0%, 0.1%)

	Inferior	No	Very Low	39.2% (0.9%, 62.6%)	-0.8 (-1.2, -0.5)	67.1 (63, 71.3)	95.4% (94.6%, 96.3%)	3.3% (2.6%, 4%)
			Low	38.3% (15.7%, 54.4%)	-1.7 (-1.9, -1.5)	32.7 (30.8, 34.7)	94.7% (93.8%, 95.6%)	0% (0%, 0%)
			Medium	38% (15.1%, 55.9%)	-2 (-2.2, -1.8)	36.2 (34, 38.5)	93.7% (92.7%, 94.6%)	0.1% (0%, 0.2%)
			High	35.3% (17.8%, 50.6%)	-4.7 (-4.9, -4.5)	47.8 (45.3, 50.4)	84.5% (83.1%, 85.9%)	0% (0%, 0%)
			Very High	32.5% (16.9%, 47.6%)	-7.5 (-7.6, -7.3)	76.1 (73.1, 79.2)	60.8% (58.9%, 62.7%)	0% (0%, 0%)
		Yes	Very Low	39.5% (0.9%, 62.6%)	-0.5 (-0.8, -0.2)	64.9 (60.9, 68.9)	95.5% (94.7%, 96.3%)	2.9% (2.2%, 3.5%)
		Yes	Low	39.5% (18.4%, 55%)	-0.5 (-0.7, -0.3)	28.4 (26.8, 30.1)	95.8% (95%, 96.6%)	0% (0%, 0%)
			Medium	39.5% (16.1%, 56.5%)	-0.5 (-0.7, -0.3)	31.3 (29.5, 33.2)	94.9% (94.1%, 95.8%)	0% (0%, 0%)
			High	38.3% (18.2%, 54.9%)	-1.7 (-1.9, -1.5)	26.8 (25.2, 28.5)	93.8% (92.9%, 94.8%)	0% (0%, 0%)
			Very High	36.6% (22.3%, 50.2%)	-3.4 (-3.6, -3.2)	30.2 (28.6, 31.8)	88.5% (87.2%, 89.7%)	0% (0%, 0%)
20%	Superior	Yes	Very Low	38.4% (3.8%, 64.7%)	-1.6 (-1.9, -1.3)	72.4 (67.8, 76.9)	95% (94.1%, 95.9%)	4% (3.2%, 4.7%)
			Low	36.8% (14.6%, 53.1%)	-3.2 (-3.4, -2.9)	43.2 (40.7, 45.8)	91.7% (90.6%, 92.8%)	0.2% (0%, 0.4%)
			Medium	35.6% (12.6%, 56.1%)	-4.4 (-4.6, -4.1)	55.2 (52.2, 58.3)	88.6% (87.4%, 89.9%)	0.1% (0%, 0.2%)
			High	30.8% (5.9%, 46.8%)	-9.2 (-9.4, -9)	117.8 (112.8, 122.7)	59.7% (57.8%, 61.6%)	0.6% (0.3%, 0.9%)
			Very High	26% (5.1%, 44.7%)	-14 (-14.2, -13.7)	225.3 (218.8, 231.8)	21.8% (20.2%, 23.5%)	1.4% (0.9%, 1.8%)
	Similar	No	Very Low	37.9% (-1.7%, 65.3%)	-2.1 (-2.4, -1.8)	77.2 (72.3, 82.2)	93.9% (92.9%, 94.8%)	5.2% (4.3%, 6.1%)
			Low	34.6% (12%, 52.7%)	-5.4 (-5.6, -5.2)	64.9 (61.5, 68.4)	83.9% (82.4%, 85.3%)	0.3% (0.1%, 0.5%)
			Medium	33.3% (8%, 51.5%)	-6.7 (-7, -6.5)	81.9 (77.8, 85.9)	80.6% (79%, 82.2%)	0.4% (0.2%, 0.7%)
			High	26.1% (2.5%, 43.4%)	-13.9 (-14.2, -13.7)	229.7 (222.4, 236.9)	27% (25.3%, 28.8%)	3.2% (2.5%, 3.8%)

			Very High	20.3% (1.7%, 39.8%)	-19.7 (-19.9, -19.5)	419.3 (410.4, 428.2)	1.9% (1.3%, 2.4%)	9.4% (8.2%, 10.5%)
		Yes	Very Low	39.1% (4.9%, 66.9%)	-0.9 (-1.2, -0.6)	69.2 (64.7, 73.6)	94.9% (94%, 95.7%)	3.6% (2.9%, 4.4%)
			Low	38.5% (17.1%, 54.8%)	-1.5 (-1.7, -1.3)	34 (32.1, 35.9)	94.6% (93.8%, 95.5%)	0% (0%, 0%)
			Medium	37.8% (11.1%, 55.8%)	-2.2 (-2.4, -1.9)	39.1 (36.7, 41.6)	93.4% (92.4%, 94.4%)	0.1% (0%, 0.3%)
			High	35.1% (17.1%, 54%)	-4.9 (-5.1, -4.7)	51.9 (49.2, 54.7)	84% (82.6%, 85.5%)	0% (0%, 0%)
			Very High	31.6% (11.3%, 46.6%)	-8.4 (-8.6, -8.2)	95.1 (91.4, 98.9)	58.8% (56.9%, 60.7%)	0.2% (0%, 0.3%)
	Inferior	No	Very Low	39.1% (5.4%, 66.5%)	-0.9 (-1.2, -0.5)	68.8 (64.5, 73.1)	95.1% (94.2%, 95.9%)	2.9% (2.2%, 3.5%)
			Low	38.4% (17.7%, 57%)	-1.6 (-1.8, -1.4)	34.1 (32, 36.1)	94.3% (93.4%, 95.2%)	0% (0%, 0%)
			Medium	37.9% (14.8%, 59.1%)	-2.1 (-2.3, -1.9)	35.3 (33.2, 37.5)	94.3% (93.4%, 95.2%)	0% (0%, 0.1%)
			High	35.6% (16.3%, 51.6%)	-4.4 (-4.6, -4.2)	44.6 (42.2, 47)	85% (83.6%, 86.4%)	0% (0%, 0%)
			Very High	33.1% (16.8%, 47.1%)	-6.9 (-7.1, -6.8)	68.8 (65.9, 71.6)	65.3% (63.5%, 67.2%)	0% (0%, 0%)
		Yes	Very Low	39.5% (3.6%, 66.5%)	-0.5 (-0.8, -0.2)	68.3 (64.1, 72.6)	95.1% (94.3%, 96%)	2.6% (2%, 3.3%)
			Low	39.3% (20.2%, 56.6%)	-0.7 (-0.9, -0.4)	31.2 (29.4, 33)	95.2% (94.3%, 96%)	0% (0%, 0%)
			Medium	39% (17.6%, 60.4%)	-1 (-1.2, -0.7)	32.2 (30.3, 34.1)	95.2% (94.4%, 96.1%)	0% (0%, 0%)
			High	38.1% (18.9%, 54.7%)	-1.9 (-2.1, -1.7)	26.5 (24.9, 28.2)	94.1% (93.2%, 95%)	0% (0%, 0%)
			Very High	36.4% (17.5%, 50.6%)	-3.6 (-3.8, -3.5)	32.8 (31, 34.6)	86.1% (84.8%, 87.5%)	0% (0%, 0%)

Table BB. Number of participants in realistically sized trials for all 50 simulation scenarios based on sample size calculations. For each scenario, the number of participants was calculated by taking the median of the sample size calculations across the 500 simulations runs for large trials. Each run's sample size calculation used data from the first 400 participants to represent a smaller pilot study.

VE against infection	Strength of infection-acquired immunity	Hybrid immunity	Force of infection	Number of participants
0%	Superior	Yes	Very Low	1,748
			Low	4,080
			Medium	3,060
			High	1,358
			Very High	6,118
	Similar	No	Very Low	1,748
			Low	4,080
			Medium	3,060
			High	1,358
			Very High	6,118
		Yes	Very Low	1,748
			Low	4,080
			Medium	3,060
			High	1,358
			Very High	6,118
	Inferior	No	Very Low	1,748
			Low	4,080
			Medium	3,060
			High	1,358
			Very High	6,118
		Yes	Very Low	1,748
		Yes	Low	4,080
			Medium	3,060
			High	1,358
			Very High	6,118
20%	Superior	Yes	Very Low	1,748
			Low	4,080
			Medium	3,060
			High	1,358
			Very High	6,118
	Similar	No	Very Low	1,748
			Low	4,068
			Medium	3,060
			High	1,358
			Very High	6,118
		Yes	Very Low	1,748
			Low	4,080
			Medium	3,060
			High	1,358
			Very High	6,118
	Inferior	No	Very Low	1,748
			Low	4,080
			Medium	3,060
			High	1,358

			Very High	6,118
		Yes	Very Low	1,747
			Low	4,080
			Medium	3,060
			High	1,358
			Very High	6,118
